# Population-level measles RT–PCR cycle threshold values and their relationships with disease severity, transmission, and incidence metrics: a time series analysis of the 2025–2026 outbreak in Mexico

**DOI:** 10.3389/fpubh.2026.1967103

**Published:** 2026-10-08

**Authors:** Judith Carolina De Arcos-Jiménez, Ernestina Quintero-Salgado, Aurora Karina Robles-Martínez, José Luis Carrillo-Quintanar, Violeta Cassandra Vera-Cuevas, Pedro Martínez-Ayala, Christian Mendoza-Mújica, Gerardo F. Gómez-Abundis, Mauricio Alfredo Ambriz-Alarcón, Brian Rafael Rubio-Mora, Jaime Briseno-Ramirez

**Affiliations:** 1 Laboratorio Estatal de Salud Pública de Jalisco, Zapopan, Mexico; 2 Centro Universitario de Tlajomulco, Universidad de Guadalajara, Tlajomulco de Zúñiga, Mexico; 3 Centro de Investigaciones Biológicas del Noroeste, La Paz, Mexico; 4 Antiguo Hospital Civil de Guadalajara “Fray Antonio Alcalde”, Guadalajara, Mexico; 5 OPD Servicios de Salud Jalisco, Guadalajara, Mexico; 6 Banner – University Medical Center Phoenix, Phoenix, AZ, United States; 7 University of Arizona College of Medicine – Phoenix, Phoenix, AZ, United States; 8 Hospital Civil de Oriente, Tonalá, Mexico

**Keywords:** cycle threshold, disease outbreaks, effective reproduction number, measles, Mexico, population surveillance, real-time polymerase chain reaction, viral load

## Abstract

**Objectives:**

To determine whether the weekly median real-time reverse-transcription polymerase chain reaction (RT–PCR) quantification cycle (Cq) value of notified measles cases precedes the incidence, transmission, and severity metrics of an outbreak. We focused particularly on the effective reproduction number (*R*_*t*_). We also assessed whether any association persisted after adjustment for a linear time trend, specimen composition, and the outcome's own past.

**Methods:**

We conducted a retrospective secondary analysis of 10,910 Cq determinations and 11,735 confirmed cases recorded in a single Mexican national case-level surveillance extract for Chihuahua and Jalisco. Twelve reported weekly metrics of incidence, transmission, and severity were examined by cross-correlation, four nested lagged regression models (M1–M4), formal Toda–Yamamoto Granger tests, and a secondary calibrated-lag autoregressive model. Moving-block bootstrap intervals were the primary method for regression inference. Autocorrelation-robust and classical methods were used in sensitivity analyses. Individual Cq determinations were analyzed separately against collection timing, severity, and vaccination history.

**Results:**

Selected-lag associations were negative across all reported metric families, at leads of 1–9 weeks. The strongest negative correlation between Cq and DGE-derived *R*_*t*_ occurred at +8 weeks in the second Chihuahua wave (*r* = −0.745). In Jalisco, it occurred at +7 weeks (*r* = −0.464). Only Jalisco supported the fully adjusted model 4 (M4). There, bootstrap intervals excluded zero for both the laboratory-positive-case and final surveillance-confirmed-case *R*_*t*_ estimates but not for the two severity proportions. Excluding up to four endpoint weeks preserved the direction of both *R*_*t*_ estimates. At the individual level, Cq increased with zero, one, and two documented vaccine doses (0.761 cycles per dose, 95% CI 0.589 to 0.932) and was 1.02 cycles lower in hospitalized cases.

**Conclusions:**

Aggregate Cq showed the most consistent retrospective leading association with *R*_*t*_. The retrospective associations occurred at data-calibrated lags of 7–8 weeks during the second Chihuahua wave and in Jalisco. Conversely, leak-free forecasting supported incremental gain in one analytic unit at a median 2-week horizon. The operational predictive horizon of aggregate Cq therefore remains unvalidated. If prospectively validated, weekly aggregate Cq could complement conventional measles surveillance using laboratory information that is already routinely collected.

## 1 Introduction

Timely recognition of epidemic acceleration is central to outbreak control. However, routine surveillance indicators—notified cases, hospitalizations and deaths—describe events that have already occurred. Each is separated from the transmission event by the incubation period and by delays in care, testing and notification. The current measles resurgence in the Americas makes this limitation urgent. After two decades of sustained elimination, the region lost its verification as free of endemic transmission in November 2025. By epidemiological week 29 of 2026, 47,026 confirmed cases and 48 deaths had been reported across 17 countries and territories. Mexico contributed 12,233 cases, the second-largest national total (1). The Pan American Health Organization (PAHO) figures illustrate the scale of an epidemic associated in Mexico with subnational immunity gaps and declining vaccination coverage (2, 3).

In this setting, delays from symptom onset to laboratory confirmation can make case data intrinsically retrospective. Nowcasting is therefore needed to estimate the present epidemic state (4). A signal available earlier, without a new specimen or field procedure, could therefore strengthen routine surveillance.

One candidate is the quantification cycle (Cq), a value generated by real-time RT–PCR and inversely related to the amount of target nucleic acid in a specimen. A single Cq value depends strongly on specimen timing and is not a stand-alone clinical measure. Across many specimens collected in the same week, however, its distribution may summarize whether infections are being sampled earlier or later in their course. This population-level premise was established for Severe Acute Respiratory Syndrome Coronavirus 2 (SARS-CoV-2). During epidemic growth, a larger share of specimens may come from recently infected people with higher measured viral burden and lower Cq. During decline, later resolving infections may shift the distribution upward. Cross-sectional viral-load distributions were shown to contain information about epidemic trajectory (5). Subsequent work related population-level Cq to transmission in Hong Kong. Other studies found that Cq-derived growth estimates slightly preceded incidence-derived estimates in Madagascar and tracked transmission and outcome metrics across pandemic years in western Mexico (6–8). National influenza data provide evidence beyond SARS-CoV-2 (9). These studies support an association with epidemic phase, not an already validated operational warning system.

Population-level evidence of this kind has not previously been established for measles. The biological prerequisites are supported only at the individual level. Validated measles RT–PCR assays show a log-linear relationship between Cq and target copy number (10). RNA detection in throat specimens changes sharply with time from rash onset (11), although low-level RNA may persist after acute disease (12). A recent outbreak investigation also reported mean measles Ct values explicitly by days from symptom and rash onset (13). These individual and small-outbreak observations support timing as a biological prerequisite, but they do not establish the population-level weekly signal tested here.

Transfer from other viruses cannot be assumed. Measles has a different and more sharply defined shedding window. Its transmissibility is very high, although reported basic reproduction numbers vary substantially across settings (14). Its case definition is anchored on rash onset. In Mexico, laboratory testing of suspected cases is mandatory rather than demand-driven. A narrow sampling window could sharpen the relation between Cq and epidemic phase, while vaccination, specimen timing and between-laboratory variation could weaken it. These competing features make a measles-specific population analysis necessary.

We therefore analyzed the two largest and most sustained outbreaks of Mexico's 2025–2026 epidemic, in Chihuahua and Jalisco. We asked whether weekly median Cq preceded incidence, transmission, and severity metrics, and at what data-calibrated lag. We then assessed whether any association persisted after accounting for time trend, specimen composition and the outcome's own past. The analyses used cross-correlation, covariate-adjusted lagged models, formal Toda–Yamamoto Granger tests in both directions and a secondary calibrated-lag autoregressive model. By testing a routinely recorded laboratory by-product, the study evaluates whether existing data could, after prospective validation, complement conventional measles surveillance and support earlier public-health assessment.

## 2 Materials and methods

Reporting follows the STROBE statement for observational studies (15) and its RECORD extension for routinely collected health data (16), adapted to a primary weekly time-series analysis and a secondary individual-level analysis. Supplementary Tables S7, S8 contain the completed checklists and identify the location of each item.

### 2.1 Setting

The National Epidemiological Surveillance System (SINAVE) operates Mexico's surveillance of febrile exanthematous illness (*enfermedad febril exantemática*, EFE). The system requires every case meeting the syndromic definition to be notified, investigated, and laboratory-tested. Each federal entity operates a State Public Health Laboratory that performs the molecular diagnosis. The General Directorate of Epidemiology (DGE) assigns each case's final classification (confirmed, discarded, or under study) on combined epidemiological and laboratory grounds (17). The study covers Chihuahua, in the north, and Jalisco, in the west. We restricted the analysis *a priori* to these two states because they had the country's largest and most sustained outbreaks and could support a weekly time-series design. Together, they contributed 11,735 confirmed cases within the analytic windows. A weekly median Cq was available for 76 of the 81 weeks spanned.

This restriction was important because measles incidence is spatially heterogeneous. Transmission propagates hierarchically from large population centers to smaller ones (18), while communities below the critical size for sustained transmission can show sporadic clusters separated by weeks without notifications (19, 20). Such gaps yield neither a weekly median Cq nor a reproduction-number estimate and would make the planned weekly comparisons unstable.

Population denominators were derived from officially reported case counts and incidence rates for each state and year (approximately 3.97 million inhabitants for Chihuahua and 8.79–8.98 million for Jalisco). This preserved consistency with the published rates used to construct the analytic series. As an external check rather than a replacement denominator, we compared these values with the published mid-year projections of Mexico's National Population Council (CONAPO) (21). Agreement was within 2%: the CONAPO projections were 4.04 and 4.09 million for Chihuahua and 8.90 and 8.98 million for Jalisco in 2025 and 2026, respectively.

### 2.2 Data sources and access

The study used a de-identified national EFE/SINAVE case-level surveillance extract obtained through Mexico's transparency and access-to-information framework on 1 August 2026. For each notified case, the source record included the laboratory result together with the epidemiological, clinical, vaccination, and final-classification information used in the analyses. The source variables and their analytic derivations are reported in Supplementary Table S0a.

The regional PAHO total cited in the Introduction is a surveillance snapshot through epidemiological week 29 of 2026. The frozen analytic extract spans February 2025 through late July 2026. Its national descriptive total therefore differs in both observation period and reporting cut-off.

The reduced DGE open EFE data set (22) and the official epidemic report were consulted only to verify aggregate counts and coding and to derive the population denominators. They did not supply any individual-level field analyzed here. Individual-level and time-series models were restricted to Chihuahua and Jalisco. Records from all federal entities in the same frozen extract were used only for the national descriptive context.

### 2.3 Population and eligibility criteria

We retrospectively reviewed the EFE surveillance records for Chihuahua and Jalisco contained in the extract obtained on 1 August 2026. Two nested national definitions govern who enters the system. Syndromic EFE surveillance defines a suspected case as a febrile illness with a rash of more than 3 days' duration in a person under 15 years of age, with the age criterion waived during outbreaks (23). A probable measles case is any person, of any age, with fever and a maculopapular rash plus at least one of cough, coryza, conjunctivitis, or lymphadenopathy (17). Because both states were in a declared outbreak throughout the study period, the age criterion did not apply. We selected all individuals notified in the two states from this source. We checked surveillance identifiers for duplicate records. None were found among the 21,390 records from Chihuahua and Jalisco.

The study period was defined by rash onset on or after 1 January 2025. Individuals with earlier onset were excluded. This exclusion appears in the study flowchart, so the selection chain starts from a single population. Two analytic subsets were then derived from the same source records. In the Cq subset, eligibility required a measles RT–PCR performed on a throat swab at the State Public Health Laboratory. This specimen and assay are prescribed by the national laboratory guidelines (24). The numerical Cq value also had to fall within the operational range 0 < Cq < 40 cycles. Non-positive entries and values at or above the assay's 40-cycle amplification limit were excluded. This was a data-cleaning rule, not the criterion by which a case received its final surveillance classification.

In the confirmed-case subset, eligible records had a DGE classification of confirmed (17). Discarded and under-investigation cases were excluded. The confirmed-case subset required the rash-onset week to fall within the corresponding state window. The Cq subset additionally required the specimen-collection week to fall within that window, so every weekly median and every person-level comparison refer to complete Monday–Sunday weeks. The state-specific windows are defined in Section 2.6.

Definitions and derivation rules for classification, Cq, hospitalization, complications, vaccination, and the adjustment variables are provided in Supplementary Table S0a.

### 2.4 Laboratory diagnosis and comparability of Cq values

The cycle threshold (Ct) of a real-time RT–PCR reaction is termed the quantification cycle (Cq) under the Minimum Information for Publication of Quantitative Real-Time PCR Experiments (MIQE) nomenclature (25). We use Cq throughout this article. It is inversely related to the amount of target nucleic acid in the specimen and is used here as a semi-quantitative, relative proxy rather than an absolute copy-number measurement. For measles, validated assays show a log-linear Cq–target-copy relationship within their quantifiable range (10).

Measles diagnosis in both states followed the single national algorithm issued by the Institute of Epidemiological Diagnosis and Reference (InDRE), the national reference laboratory and the standard-setting authority for EFE diagnosis in Mexico (24). Under that algorithm, serum and a throat swab are taken simultaneously from every probable case. The swab is collected within 5 days of rash onset, or within 7 days during an outbreak, and is transported in viral transport medium at 2–8 °C. Every swab is tested by real-time RT–PCR for both measles and rubella at the State Public Health Laboratory. The national network prescribes the same measles target, primer/probe set, human RNase P internal control and thermal profile. Exact assay specifications are given in Supplementary methods. Participating laboratories undergo external performance evaluation, and InDRE retains responsibility for genotyping. We therefore analyzed the state series under a shared assay framework. No study-specific interlaboratory calibration panel was available. Comparisons therefore emphasized within-state temporal variation. Residual differences between laboratories cannot be excluded.

Only determinations performed on throat swabs as part of the measles testing panel and greater than zero but below 40 cycles were retained. Lower values indicate higher measured target abundance. The analysis rested on relative variation over time within the shared assay framework.

### 2.5 Data metrics for time-series analysis

All series were aggregated to epidemiological weeks beginning on Monday, with cases and clinical outcomes dated by rash onset and Cq determinations by specimen collection. The weekly median Cq was computed from all analyzable determinations of the corresponding week and was left undefined when fewer than five determinations were available.

Twelve outcome metrics were reported across three domains. Incidence metrics comprised weekly counts of RT–PCR-positive and DGE-confirmed cases and their rates per 100,000 inhabitants. Transmission was represented by the effective reproduction number from each case series. Severity metrics comprised weekly counts of hospitalized and pneumonia cases, their rates per 100,000, and their proportions of confirmed cases.

Counts and their corresponding rates are deterministic linear transformations within each state-year denominator. The 12 reported metrics therefore represent eight distinct weekly quantities rather than 12 independent outcomes. Proportions normalize for case volume and do not scale directly with the number of cases. Their precision and stability still depend on the weekly denominator.

Two *R*_*t*_ outcomes were retained to represent different operational case series. *R*_*t*_ (RT–PCR cases) was calculated from laboratory-positive case counts. *R*_*t*_ (DGE cases) was calculated from cases assigned a final confirmed classification by the national surveillance process. Concordance between them tests whether an association depends on the input case definition. This comparison is not independent replication because both series come from the same surveillance system and the laboratory-positive subset is nested within the confirmed-case population.

The reproduction number was estimated from daily counts by rash onset using the instantaneous estimator of Cori et al. (26) in EpiEstim. Estimation used 7-day sliding windows and a parametric serial interval with a mean of 11.1 days (SD 2.47). The gamma distribution had been applied to measles incidence dated by rash onset (27) and was derived from household studies of paired cases (28). Daily estimates were then averaged by week.

### 2.6 Statistical analysis

The analyses addressed four progressively narrower questions. First, cross-correlation described whether lower weekly Cq values occurred before higher incidence, transmission, or severity metrics and identified an exploratory lag. Second, nested regressions asked whether the association at that lag persisted after adjustment for specimen composition and secular time. Third, temporal models asked whether lagged Cq added information beyond the outcome's own recent history. These models distinguish precedence from contemporaneous correlation but do not establish causality. Fourth, a rolling out-of-sample analysis tested whether adding Cq reduced prediction error. The individual-level analyses were a separate biological consistency check. The detailed implementation and sensitivity specifications are given in Supplementary methods.

#### 2.6.1 Sex and gender

The surveillance system records sex as a binary administrative field completed at notification. No individual was excluded on the basis of sex, and both sexes are represented throughout. Sex is taken into account in two ways: as the weekly percentage of women among the sampled specimens, a composition covariate in the adjusted specifications, and as an adjustment term in the individual-level models.

#### 2.6.2 Analytic series and notation

The two states experienced sequential outbreaks and were analyzed as separate series. As described with the epidemic curves in Results, Chihuahua was divided at its intervening trough into two sub-waves that share the boundary week, whereas Jalisco was treated as a single wave. The undecomposed Chihuahua series was retained as a sensitivity analysis. Throughout, *t* indexes the weeks of a series. *C*_*t*_ is the weekly median Cq, and *Y*_*t*_ is the outcome metric for the same week. Finally, *k* denotes a lag in weeks and k^*^ the calibrated lag.

The epidemiological week beginning 27 July was incomplete when the extract was received and was excluded. The primary Jalisco window therefore ended with the complete week beginning 20 July. Endpoint sensitivities progressively removed additional terminal weeks and repeated the principal *R*_*t*_ analyses, as detailed in Supplementary methods.

#### 2.6.3 Calibration stage

Cross-correlations were computed as shown in Equation 1:


$$\begin{array}{l} r\left(k\right)=\text{cor}\left(C_{t},Y_{t+k}\right),\;k=-10,\ldots ,+10, \end{array}$$ (1)

over the *n*_*k*_ weeks in which both terms are observed. A positive *k* denotes that Cq precedes the outcome, following the convention of our SARS-CoV-2 report (8). Because a lower Cq corresponds to a higher viral load, the hypothesis predicts a negative coefficient at a positive lag. Coefficients required at least 8 weekly pairs and were compared with the approximate white-noise band $\pm 1.96/\sqrt{n_{k}}$, computed from the pairs available at each lag (29). The calibrated lag was


$$\begin{array}{l} k^{*}=\underset{1\leq k\leq 10}{\mathrm{argmin}}r\left(k\right), \end{array}$$ (2)

Equation 2 selects the lag of the most negative coefficient rather than of the largest in absolute value. Lag zero is excluded because a contemporaneous association is not the hypothesis under test. Severity counts and rates inherited k^*^ from the incidence metrics of the same series, because they were computed from a subset of the confirmed cases indexed by the same rash-onset week. Severity proportions and the reproduction number retained their own calibrated lag. The selection is an exploratory calibration, not a test: the lag was chosen in the same data on which the correlation is reported.

As a sensitivity to serial structure, an autoregressive integrated moving average (ARIMA) filter selected from the Cq series was applied to both Cq and the outcome before the residual cross-correlation was recalculated. Selection, interpolation and lag-search rules are specified in Supplementary methods.

#### 2.6.4 Covariate-adjusted analysis at the data-calibrated lag

At k^*^, four nested specifications were fitted by ordinary least squares, of which the fullest is


$$\begin{array}{l} Y_{t+k^{*}}=\beta_{0}+\beta_{1}C_{t}+\beta_{2}A_{t}+\beta_{3}F_{t}+\beta_{4}s_{t}+\beta_{5}I_{t}+\beta_{6}N_{t}+\varepsilon_{t}, \end{array}$$ (3)

with β_1_ the quantity of interest throughout. The four models add the terms of Equation 3 cumulatively in the order shown: M1 retains *C*_*t*_ alone. M2 adds the weekly age and sex composition of the specimens (*A*_*t*_, *F*_*t*_). M3 adds the linear secular trend *s*_*t*_, the ordinal position of the Cq week within the window assigned before listwise deletion. M4 adds the percentage of Indigenous individuals *I*_*t*_ and the number of determinations behind the weekly median *N*_*t*_. Covariates were measured in the week of the Cq determination rather than in that of the outcome, because they describe the individuals whose viral load was measured. M1 reproduces the cross-correlation by construction and provides no independent evidence. Models M2–M4 assess the robustness of the selected-lag association to measured weekly covariates, but do not constitute independent confirmation.

Moving-block bootstrap percentile intervals were the primary regression inference (30, 31). Resampling was conditional on the data-calibrated lag and therefore does not incorporate uncertainty from lag selection. Classical standard errors and Newey–West heteroskedasticity- and autocorrelation-consistent (HAC) standard errors were sensitivity analyses (32, 33). Specifications were fitted only when at least five observations per predictor, excluding the intercept, were available. Specifications that failed this criterion are reported as not estimable (“n.e.”). In the formal VAR-order analysis, numerical results below that threshold are retained only as descriptive sensitivity results and are excluded from inferential conclusions. We assess the estimability of the secondary calibrated-lag model separately.

#### 2.6.5 Temporal analysis: Toda–Yamamoto Granger causality and a calibrated-lag autoregressive model

Augmented Dickey–Fuller (ADF) (34, 35) and Kwiatkowski–Phillips–Schmidt–Shin (KPSS) (36) tests were used to assess whether level stationarity could be assumed. Granger causality was assessed by the Toda–Yamamoto procedure (37, 38). This procedure estimates a bivariate vector autoregression (VAR) in levels with *p*+*d*_max_ lags and applies the Wald restriction to the first *p* lags only,


$$\begin{array}{lll} Y_{t} & = & \alpha +\overset{p+d_{\max}}{\underset{i=1}{\sum }}\phi_{i}Y_{t-i}+\overset{p+d_{\max}}{\underset{i=1}{\sum }}\gamma_{i}C_{t-i}+u_{t}, \end{array}$$

$$\begin{array}{lll} H_{0}:\gamma_{1} & = & \cdots =\gamma_{p}=0, \end{array}$$ (4)

whose statistic is χ^2^ with *p* degrees of freedom. We set *d*_max_ = 1, selected *p* by the Akaike information criterion (39), and tested both temporal directions. Because the admissible VAR lag order is far smaller than the calibrated lags, a secondary autoregressive model targeted k^*^ directly,


$$\begin{array}{l} Y_{t}=\alpha +\overset{q}{\underset{i=1}{\sum }}a_{i}Y_{t-i}+bC_{t-k^{*}}+\eta_{t},\;H_{0}:b=0, \end{array}$$ (5)

with a single own lag (*q* = 1) in the principal specification. Equation 5 estimates an autoregressive association at a data-calibrated lag. It is not a Toda–Yamamoto or other formal Granger-causality test. First-difference tests, a two-own-lag model and Jalisco models with *d*_max_ = 2 were sensitivity analyses. Test settings, contiguity rules and additional-lag logic are detailed in Supplementary methods.

#### 2.6.6 Individual-level analysis of the Cq

Because the exposure is a weekly median, we examined whether its component individual determinations behave as measles biology predicts. Each determination was related to the onset-to-collection interval, hospitalization, recorded complications and documented vaccine doses. Since collection timing is itself associated with Cq, every contrast was also adjusted for that interval, age, sex, and state. These covariates are collected in **w**_*i*_. For the vaccination gradient,


$$\begin{array}{l} \text{C}{\text{q}}_{i}=\alpha +\tau V_{i}+w_{i}^{⊤}\lambda +\epsilon_{i}, \end{array}$$ (6)

In Equation 6, *V*_*i*_∈{0, 1, 2} is the number of documented doses, entered as a single linear term. Thus, τ is the gradient in Cq cycles per dose. Severity contrasts replace τ*V*_*i*_ with a binary indicator. Each of the five two-way interactions adds a product term. The procedure was repeated within each state, with the state term dropped. Crude comparisons used the Wilcoxon rank-sum test (40, 41) and the Kruskal–Wallis test (42). No group of fewer than 20 determinations was reported separately.

Sensitivities treated dose as a factor, modeled age flexibly, tested dose by state, compared collection timing across dose groups, and added weekly vaccination, timing, and Cq-completeness terms to the Jalisco *R*_*t*_ models. The completeness term was not treated as censoring at the assay limit. Specifications are detailed in Supplementary methods.

The analysis as a whole is exploratory, and no multiplicity correction was applied across its axes, the time-series specifications, or the five interactions. Holm's step-down correction (43) was applied only within each family of pairwise rank-sum contrasts involving more than two groups, namely the three vaccination strata and the four cells of vaccination by hospitalization. These are the adjusted *p*-values reported in Section 3.6 and shown as brackets with the individual-level results. Marginal *p*-values elsewhere are hypothesis-generating.

#### 2.6.7 Secondary analysis: out-of-sample predictive gain

Because temporal precedence does not imply predictive value, incremental gain was evaluated by rolling-origin forecasting, training on weeks 1 to *i* and predicting week *i*+1. Each configuration compared a model containing the outcome's past with the same model plus lagged Cq. Positive skill denotes a proportional reduction in out-of-sample root mean squared error. The *nowcast* framing allowed the previous outcome value, whereas the *lead-time* framing lagged every predictor equally. Comparators, wave definitions, and fixed vs. training-fold lag selection formed a complete 242-configuration grid. This secondary analysis was not pre-specified and had 6–12 folds per configuration. Complete methods and results are in Supplementary Tables S6a, b.

#### 2.6.8 Software

Analyses were performed in R version 4.5.3. Data management used data.table 1.18.2.1, and graphics used ggplot2 4.0.2 and patchwork 1.3.2. The reproduction number was estimated with EpiEstim 2.2.5. HAC inference and Wald tests used sandwich 3.1.1 and lmtest 0.9.40. The augmented Dickey–Fuller (35) and KPSS (36) stationarity tests used tseries 0.10.62. ARIMA prewhitening used forecast 9.0.2. Vector autoregressions were fitted by ordinary least squares, equation by equation, so that the same HAC estimators were applied throughout.

## 3 Results

### 3.1 Descriptive characteristics

The EFE/SINAVE case-level surveillance extract contained 21,390 individuals from Chihuahua and Jalisco notified to the surveillance system, of whom 19,093 had rash onset within the study period. Of these, 18,297 had a measles RT–PCR throat-swab block. Removing 4,943 records without a numeric entry left 13,354 records with a numeric value in the Cq field. Of these, 2,148 had a recorded value of 0 and were excluded from quantitative Cq analysis, leaving 11,206 non-zero numeric entries. Excluding two implausible raw entries (2,016 and 3,078) left 11,204 analyzable Cq values satisfying 0 < *Cq* < 40. In the final-classification field, 12,045 individuals were classified as confirmed measles cases. After restriction to complete state-specific epidemic wave windows, 10,910 Cq determinations and 11,735 confirmed cases entered the analysis (Figure 1).

**Figure 1:**
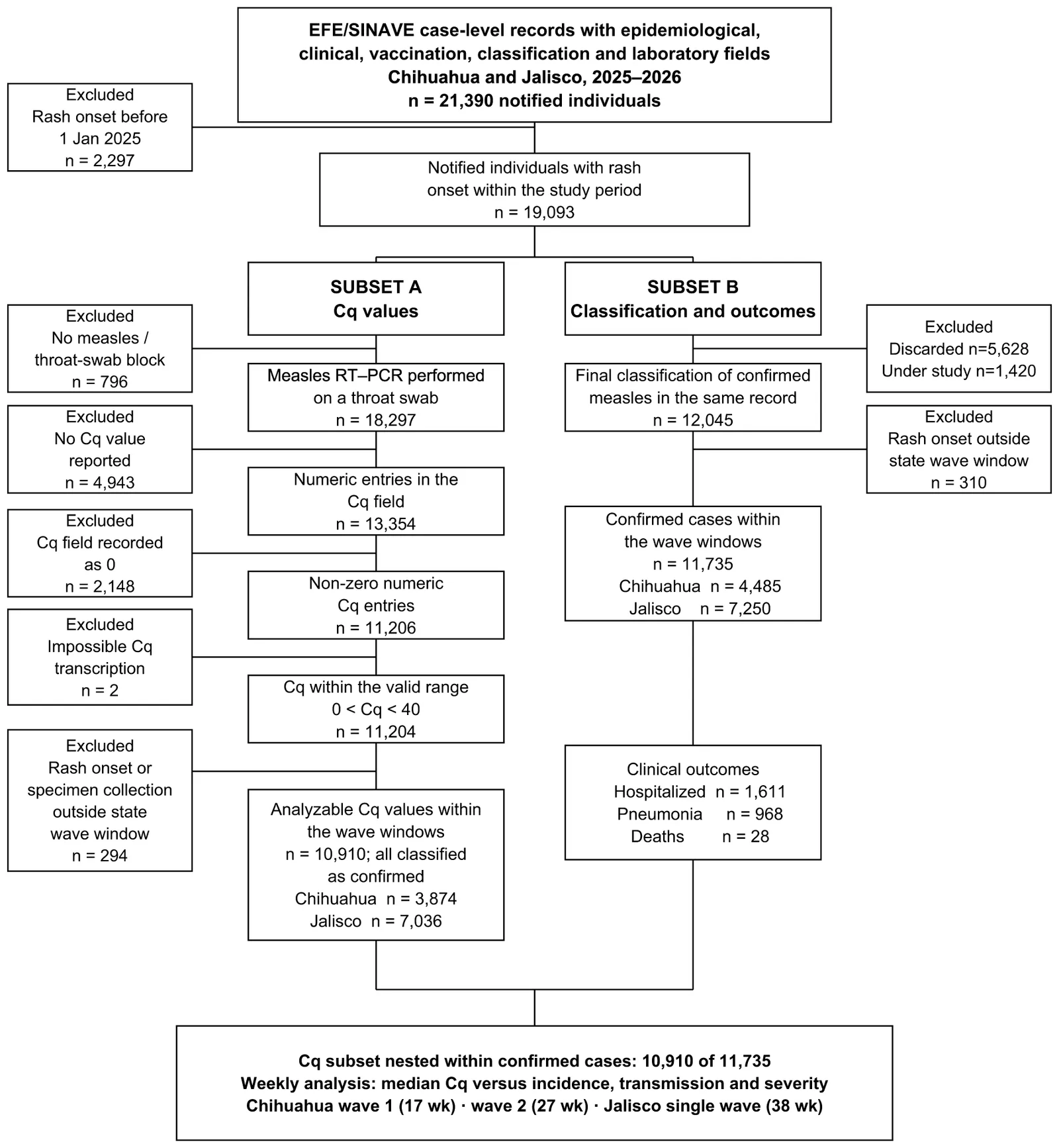
Flowchart of case and test selection from the de-identified EFE/SINAVE case-level surveillance extract. Laboratory, classification, vaccination, and clinical information were obtained from the same case record. The Cq and confirmed-case populations were defined from those records. No linkage to another person-level database was performed. Exclusion boxes appear alongside the step at which each was applied. Entries with the Cq field recorded as 0 were excluded from quantitative Cq analysis. The source extract did not establish a laboratory or data-entry mechanism for that recorded value. Two implausible raw entries (2016 and 3078) were also excluded.

Characteristics of both analytic populations are given in Table 1. Among the 10,910 individuals contributing a Cq determination, the median age was 20 years [interquartile range (IQR) 8–31], and 49.4% were women (*n* = 5, 387). The median interval from rash onset to specimen collection was 1 day (IQR 1–3), and 99.3% of specimens were collected within 7 days. The median Cq value was 24.76 cycles (IQR 21.97–27.94) and was comparable between states. The median was 24.34 in Chihuahua (IQR 21.77–27.66, *n* = 3, 874) and 24.95 in Jalisco (IQR 22.07–28.13, *n* = 7, 036).

**Table 1:** Characteristics of the two analytic populations.

Characteristic	Total	Chihuahua	Jalisco
Cq determinations (*n* = 10,910; Chihuahua 3,874, Jalisco 7,036)			
Age, years, median (IQR)	20 (8–31)	23 (9–32)	18 (8–31)
Women, *n* (%)	5,387 (49.4)	2,005 (51.8)	3,382 (48.1)
Rash onset to collection, days, median (IQR)	1 (1–3)	2 (1–3)	1 (1–2)
Collected within 7 days, %	99.3	98.3	99.8
Cq value, cycles, median (IQR)	24.76 (21.97–27.94)	24.34 (21.77–27.66)	24.95 (22.07–28.13)
Confirmed cases (*n* = 11,735; Chihuahua 4,485, Jalisco 7,250)			
Age, years, median (IQR)	20 (8–31)	23 (9–32)	18 (8–31)
Hospitalized, *n* (%)	1,611 (13.7)	950 (21.2)	661 (9.1)
Pneumonia recorded, n (%)	968 (8.2)	694 (15.5)	274 (3.8)
Deaths, *n* (%)	28 (0.2)	23 (0.5)	5 (0.1)

The Cq subset comprises the 10,910 confirmed cases with an analyzable determination and is nested within the 11,735-case confirmed subset used to derive clinical outcomes. A further 825 confirmed cases lacked an analyzable Cq. Both subsets came from the same EFE/SINAVE records, but each block retains its own denominator and no percentage is computed across blocks. Age was recorded for 10,095 of the 10,910 Cq determinations (3,467 in Chihuahua and 6,628 in Jalisco) and for 10,841 of the 11,735 confirmed cases (4,014 and 6,827, respectively). Age medians and interquartile ranges use the available values. Both subsets are restricted to the state-specific wave windows, as shown in Figure 1.

IQR, interquartile range.

All 10,910 analytic determinations had a laboratory-result date recorded in the same surveillance row. In 20 Chihuahua records, that date preceded specimen collection and was treated as a date-order inconsistency. These records were excluded only from the interval summary. Among the remaining 10,890 determinations, the median collection-to-result interval was 3 days (IQR 2–5), and 94.3% had a result recorded within 7 days. The corresponding medians were 4 days (IQR 3–6) in Chihuahua and 2 days (IQR 2–4) in Jalisco (Supplementary Table S13). These dates describe recorded result generation, not when the information became available to or was used by public-health decision-makers.

Among the 11,735 confirmed cases within the wave windows (Table 1), 1,611 (13.7%) required hospitalization, 968 (8.2%) had pneumonia recorded as a complication, and 28 deaths were registered. The proportion hospitalized differed markedly between states (Chihuahua 21.2% vs. Jalisco 9.1%), consistent with the official case-fatality figures.

### 3.2 Wave structure

The two states experienced sequential, barely overlapping epidemic waves. The Chihuahua wave was multimodal, with four local peaks exceeding 40% of the maximum weekly count (21 April, 5 May, 16 June, and 7 July 2025). These peaks were separated by a trough of 134 cases in the week of 26 May. It was therefore analyzed as two sub-waves: wave 1 (3 February to 26 May 2025, 17 weeks) and wave 2 (26 May to 24 November 2025, 27 weeks). The trough week of 26 May formed the shared boundary of both windows. The unimodal Jalisco wave peaked in the week of 9 February 2026 and was analyzed as a single window (3 November 2025 to 20 July 2026, 38 complete weeks). Figure 2 shows the series of median Cq values, cases, severity outcomes and the effective reproduction number (*R*_*t*_) for both states, together with the national epidemic containing the two outbreaks. The national panel contains 19,125 confirmed cases with an assigned state in the frozen extract. Of these, 11,735 fall within the two state-specific analytic windows.

**Figure 2:**
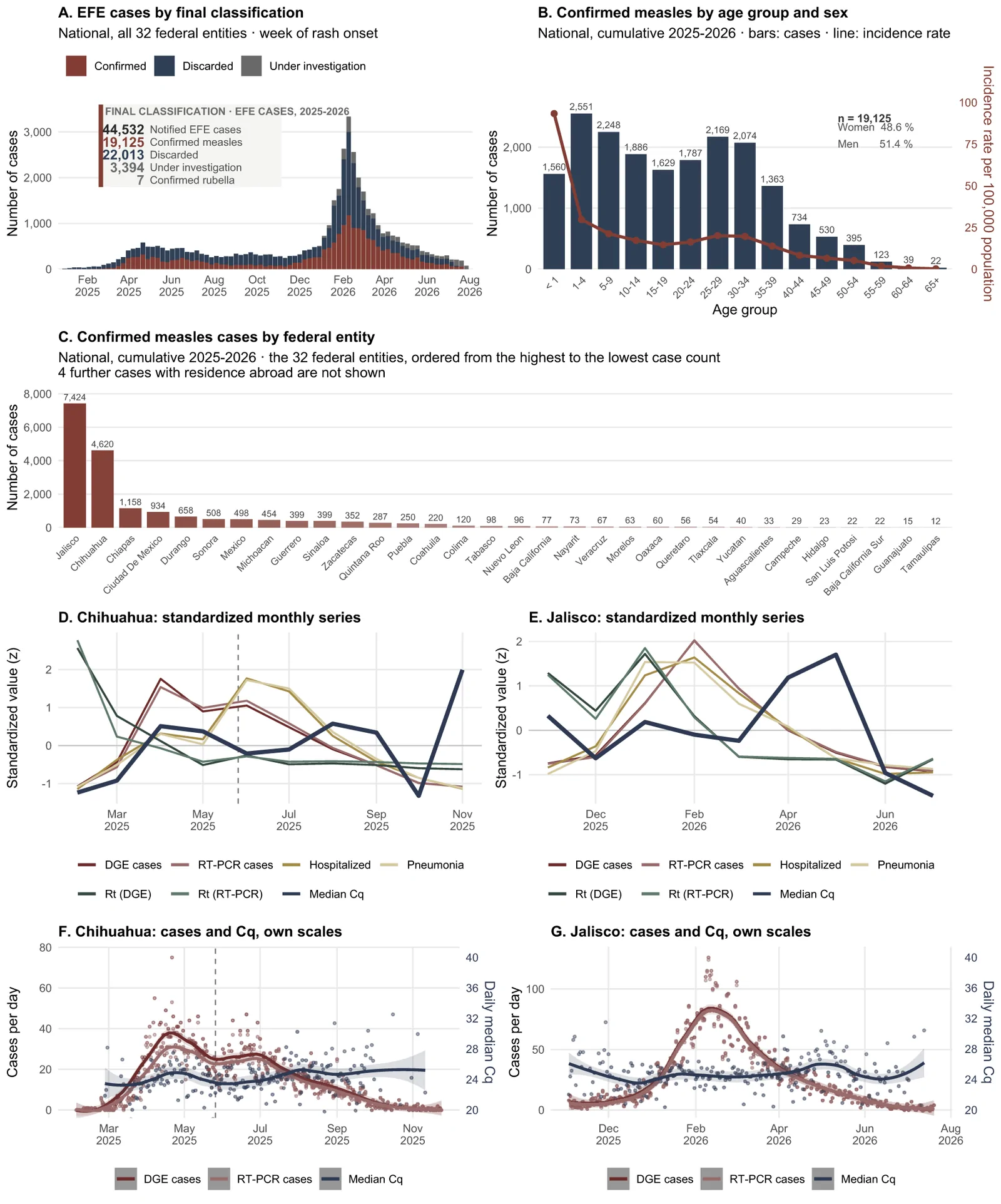
National context and time series of measles Cq values and epidemiological metrics. **(A)** Febrile exanthematous illness (EFE) cases notified in all 32 federal entities, by week of rash onset and final classification. **(B)** Confirmed measles cases by age group, with the incidence rate on the secondary axis and the distribution by sex. **(C)** Confirmed measles cases by federal entity, ordered from highest to lowest count. **(D, E)** Chihuahua and Jalisco: all series aggregated by month and standardized within each state's analytic window, *z* = (*x*−mean)/SD. **(F, G)** Chihuahua and Jalisco: daily case counts on the left axis and the daily median Cq on the right axis, bounded to 20–40 cycles, with locally weighted smooths and 95% confidence bands.

### 3.3 Cross-correlation analysis

Negative cross-correlations between weekly median Cq values and epidemiological metrics were observed across all three analytic series (Figure 3; Supplementary Tables S1a, b). Lower Cq values, corresponding to higher measured viral RNA burden, preceded increases in incidence, transmission and severity metrics. Their magnitude and lead time differed between waves.

**Figure 3:**
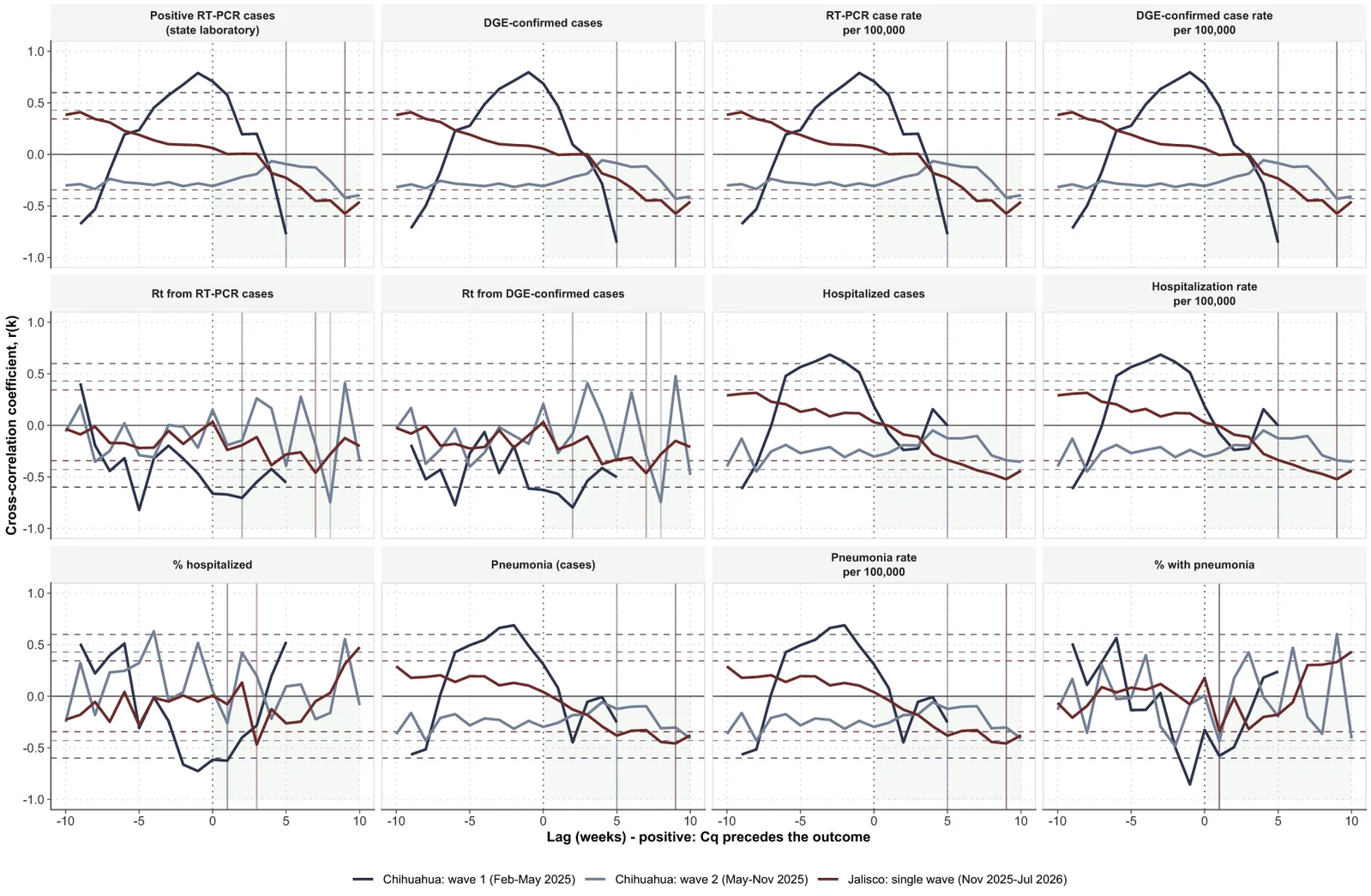
Cross-correlation functions of measles Cq values and epidemiological metrics across the three analytic series. The *x*-axis is the Cq lag *k* in weeks and the *y*-axis the cross-correlation coefficient *r*(*k*). The shaded region is the quadrant predicted by the hypothesis, a negative coefficient at a positive lag. Dashed horizontal lines are $\pm 1.96/\sqrt{n}$ conventional white-noise reference bands for each series, and the vertical line marks the calibrated lag. Complete results are given in Supplementary Tables S1a–c.

Throughout the Results, *R*_*t*_ (RT–PCR cases) denotes the estimate derived from laboratory-positive counts and *R*_*t*_ (DGE cases) the estimate derived from final confirmed-case counts. Their concordance is informative as a robustness check across two operational case definitions, not as independent replication.

In the first Chihuahua wave (Supplementary Table S1a), the strongest negative correlations occurred for DGE-confirmed cases (*r* = −0.855) and RT–PCR-positive cases (*r* = −0.774), both at a lag of +5 weeks. *R*_*t*_ showed a shorter lead time, with *r* = −0.797 at +2 weeks for *R*_*t*_ (DGE cases) and *r* = −0.703 for *R*_*t*_ (RT–PCR cases). Among severity metrics, the proportion hospitalized (*r* = −0.624) and the proportion with pneumonia (*r* = −0.578) peaked at +1 week. Eight of the 12 metrics exceeded the conventional white-noise reference band, but these estimates rest on 8–12 weekly pairs, with correspondingly wide confidence bands.

In the second Chihuahua wave (Supplementary Table S1a), the association with incidence attenuated while that with transmission strengthened. The two *R*_*t*_ outcomes showed the strongest correlations at +8 weeks: *r* = −0.745 for *R*_*t*_ (DGE cases) and *r* = −0.744 for *R*_*t*_ (RT–PCR cases), with *p* < 0.001 for both. Conversely, DGE-confirmed cases reached *r* = −0.431 at +9 weeks (*p* = 0.074). This did not exceed the conventional white-noise reference band of ±0.462. The proportion with pneumonia reached *r* = −0.439 at +1 week (*p* = 0.028). Three of the 12 metrics exceeded the conventional white-noise reference band: the two *R*_*t*_ series and the proportion with pneumonia.

In Jalisco (Supplementary Table S1b), all eight distinct weekly quantities (displayed as 12 metrics after including deterministic rate transforms) showed negative correlations that exceeded the conventional white-noise reference band. DGE-confirmed and RT–PCR-positive cases peaked at a lag of +9 weeks (*r* = −0.574 for both), as did hospitalized cases (*r* = −0.523) and recorded pneumonia cases (*r* = −0.457). *R*_*t*_ (DGE cases) peaked at a shorter lag of +7 weeks (*r* = −0.464). The severity proportions peaked earlier still: +3 weeks for hospitalization (*r* = −0.469) and +1 week for recorded pneumonia (*r* = −0.339).

Severity counts and rates, by design, inherited the incidence lag and therefore do not establish a separate temporal sequence. Among the metrics calibrated independently, severity proportions peaked at +1 to +3 weeks, generally earlier than incidence, while *R*_*t*_ peaked at +2, +8, and +7 weeks across the three series. Thus, the analyses did not show a universal sequence in which transmission preceded incidence and incidence preceded every severity metric.

Prewhitening sharpened the distinction between transmission and epidemic volume (Supplementary Table S1c). In Jalisco the selected Cq model was ARIMA(0, 0, 0), so the residual correlations were unchanged. In the second Chihuahua wave the selected ARIMA(2, 1, 0) filter retained the two *R*_*t*_ associations at +8 weeks (*r* = −0.644 and −0.682). Conversely, the incidence and severity counts no longer exceeded the white-noise reference band at their original +9-week lags. In the short first Chihuahua wave, the selected ARIMA(0, 1, 1) filter attenuated the original correlations, and none exceeded its reference band. When Chihuahua was analyzed as one undecomposed window, the raw *R*_*t*_ correlations were weaker at +8 weeks (*r* = −0.370 and −0.380) and the prewhitened optima shifted to +1 week. Incidence correlations were not distinguishable from the reference band. Complete results are given in Figure 3 and Supplementary Tables S1a–c, S12a.

### 3.4 Lagged regression analysis

Lagged regression models were fitted at the calibrated lag of each metric in the four nested specifications of Equation 3 (Figure 4; Supplementary Tables S2a, b). The number of weekly pairs differs by lag, so the estimability criterion applies unevenly. The first Chihuahua wave supported M1 only. M3 was estimable for only the two severity proportions in the second Chihuahua wave. M4 was estimable only for the four normalized Jalisco metrics. The sign of M1 is descriptive because lag calibration selected the most negative positive-lag correlation.

**Figure 4:**
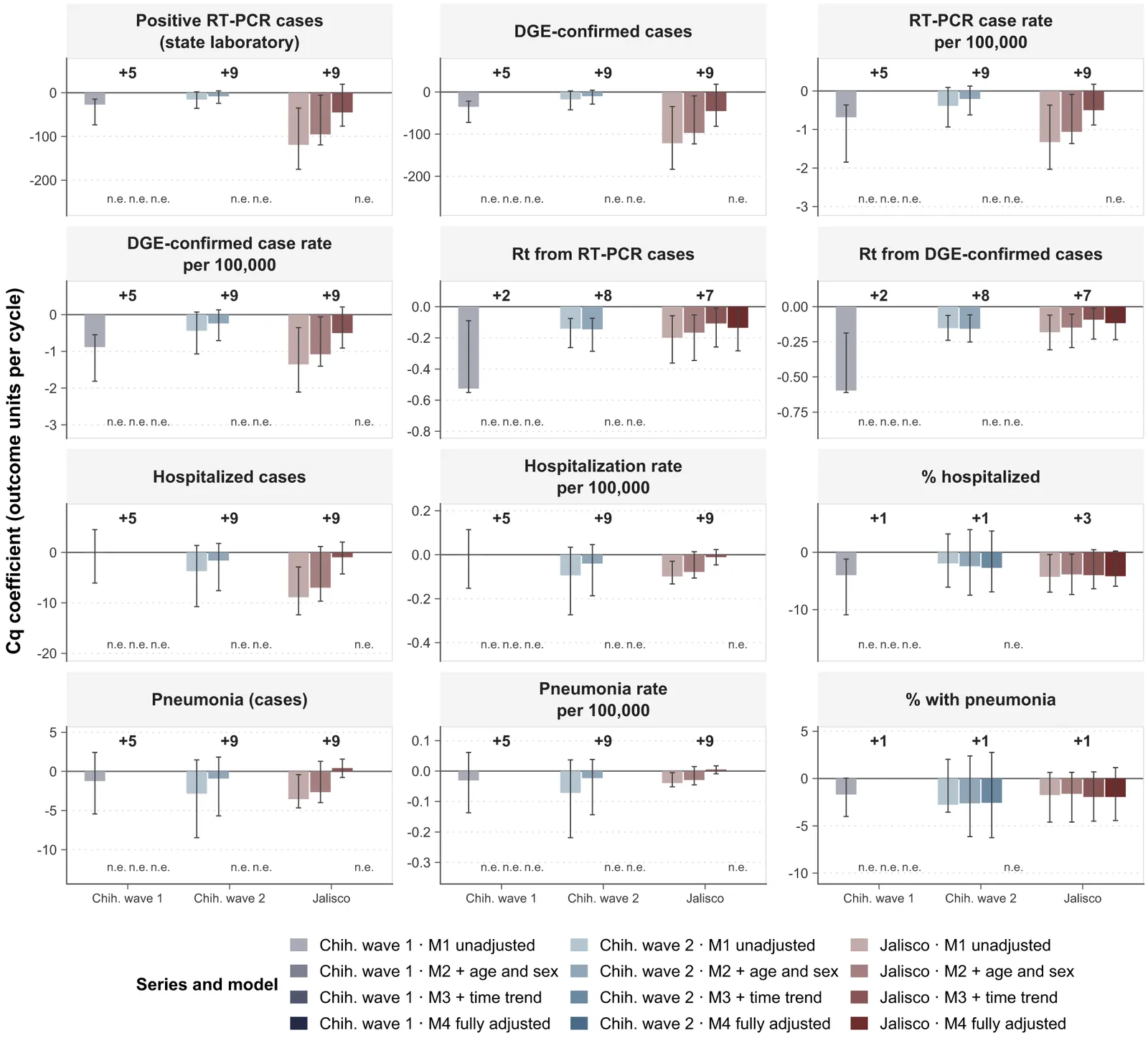
Lagged regression of each epidemiological metric on the weekly median Cq value, in four nested specifications. The calibrated Cq lag in weeks is shown above each group of bars. The *y*-axis is the regression coefficient with moving-block bootstrap 95% confidence intervals. Hue denotes the analytic series and color intensity the degree of adjustment, so attenuation is read left to right within each group. Classical and HAC inference are reported as sensitivities in Supplementary Tables S2a, b.

In Jalisco, the M1 estimate for DGE-confirmed cases was −121.57 cases per Cq cycle at +9 weeks (bootstrap 95% CI −183.60 to −34.52). At the same lag, the estimate for hospitalized cases was −8.89 (bootstrap 95% CI −12.38 to −2.87). The estimate for *R*_*t*_ (DGE cases) was −0.181 at +7 weeks (bootstrap 95% CI −0.306 to −0.060). Adjustment for weekly age and sex composition in M2 attenuated these estimates without changing their direction.

Adding the linear time trend markedly attenuated the associations with epidemic counts, whereas the *R*_*t*_ point estimates remained negative. The DGE case estimate attenuated to −45.09 (bootstrap 95% CI −81.49 to 18.83). The hospitalization estimate attenuated to −0.96 (−4.29 to 2.06). The recorded-pneumonia estimate changed sign to +0.41 (−0.76 to 1.56). Both *R*_*t*_ estimates retained bootstrap intervals below zero: −0.107 for *R*_*t*_ (RT–PCR cases) (−0.258 to −0.009) and −0.091 for *R*_*t*_ (DGE cases) (−0.231 to −0.011). The hospitalization and recorded pneumonia proportions had intervals crossing zero.

In M4, the two *R*_*t*_ estimates again retained bootstrap intervals below zero: −0.135 (−0.283 to −0.021) for *R*_*t*_ (RT–PCR cases) and −0.117 (−0.235 to −0.025) for *R*_*t*_ (DGE cases). The bootstrap intervals for the hospitalization proportion (−4.16, −5.93 to 0.22) and recorded pneumonia proportion (−1.95, −4.45 to 1.15) crossed zero. HAC inference placed the hospitalization proportion below the nominal threshold, but the bootstrap did not. This association is therefore not treated as robust. Count and rate models at their +9-week lag had 29 pairs and could not support the six-predictor M4 under the prespecified rule.

The additional Jalisco composition models supported the same restricted interpretation (Supplementary Table S10). Adding collection interval or Cq completeness separately left both *R*_*t*_ bootstrap intervals below zero. With vaccination composition added, the interval remained below zero for *R*_*t*_ (RT–PCR cases) but crossed zero for *R*_*t*_ (DGE cases). Adding vaccination composition and collection interval together preserved the negative point estimates but widened both intervals across zero. The model adding all three variables was not estimable.

The endpoint analysis likewise produced no sign reversal (Supplementary Table S9): M4 intervals for both *R*_*t*_ series remained below zero after excluding one or two endpoint weeks. After excluding four, M4 was not estimable. The M3 interval then crossed zero for *R*_*t*_ (DGE cases) but not *R*_*t*_ (RT–PCR cases). Complete regression results are given in Figure 4 and Supplementary Tables S2a, b, S9, S10.

### 3.5 Toda–Yamamoto Granger causality and calibrated-lag autoregressive models

Formal Toda–Yamamoto Granger tests and secondary calibrated-lag autoregressive models were fitted to assess temporal ordering after controlling for the outcome's own past. Stationarity tests (Supplementary Table S5) showed stationarity in levels for only four of the 39 series–variable combinations. None belonged to the two series supporting this analysis. After first differencing, 16 of 39 combinations were stationary. Causality was therefore assessed with the Toda–Yamamoto procedure (Equation 4), with *d*_max_ = 1. Because a VAR admits no gaps, each test was fitted to the longest contiguous run of weeks with both series observed, retaining the estimability criterion of at least five observations per parameter.

The first Chihuahua wave (longest contiguous run 12 weeks) admits no VAR of any order and is reported as not estimable throughout. The second Chihuahua wave yielded 4.6 observations per parameter, below the prespecified threshold. Its VAR-order results are therefore shown descriptively and were not used as inferential evidence. The Jalisco wave (38 weeks) met the threshold. Twenty-four of the 36 series–outcome combinations yielded numerical VAR-order results. Only the 12 Jalisco combinations met the prespecified criterion and are used for inference below. The calibrated-lag autoregressive models were assessed separately and met the criterion wherever estimates are reported.

The first of the two analyses was the formal Toda–Yamamoto test (Table 2). This analysis tested whether Cq values at the VAR lag order selected by the Akaike information criterion improved prediction of the outcome beyond its own past. Among the Jalisco models that met the threshold, no combination had a nominal *p* < 0.05 (minimum *p* = 0.090). The descriptive second-Chihuahua-wave results had a minimum *p* = 0.188. With HAC standard errors, only *R*_*t*_ (RT–PCR cases) in Jalisco crossed the nominal threshold (*p* = 0.044). With *d*_max_ = 2, neither Jalisco *R*_*t*_ test was nominally significant (classical *p* = 0.175 and 0.254; Supplementary Table S12b).

**Table 2:** Summary of Toda–Yamamoto Granger tests and calibrated-lag autoregressive association models.

	VAR-order Granger test				Calibrated-lag AR model		
Metric	Lag	*p*-Value	β_Cq_	Adj. *R*^2^	Lag	β_Cq_	*p*-Value
Chihuahua wave 2 (May–Nov 2025)^*a*^							
RT–PCR-positive cases	1	0.746	−0.9967	0.869	9	−1.584	0.854
DGE-confirmed cases	1	0.725	−1.001	0.904	9	−2.86	0.729
RT–PCR case rate^*b*^	1	0.746	−0.02514	0.869	9	−0.03994	0.854
DGE-confirmed case rate^*b*^	1	0.725	−0.02524	0.904	9	−0.07211	0.729
*R*_*t*_ (RT–PCR cases)	1	0.536	−0.01681	0.062	8	−0.1439	**0.013**
*R*_*t*_ (DGE cases)	1	0.318	−0.02292	0.247	8	−0.1189	**0.028**
Hospitalized cases	1	0.671	−0.6031	0.719	9	−2.435	0.570
Hospitalization rate^*b*^	1	0.671	−0.01521	0.719	9	−0.0614	0.570
% hospitalized	1	0.567	−1.203	0.142	1	−2.211	0.197
Pneumonia cases	1	0.772	−0.3449	0.703	9	−0.08266	0.981
Pneumonia rate^*vb*^	1	0.772	−0.008697	0.703	9	−0.002084	0.981
% with pneumonia	1	0.188	−1.79	0.159	1	−2.501	0.064
Jalisco wave (Nov 2025–Jul 2026)							
RT–PCR-positive cases	1	0.708	−4.452	0.851	9	−26.71	0.137
DGE-confirmed cases	1	0.703	−4.547	0.855	9	−27.04	0.134
RT–PCR case rate^*b*^	1	0.701	−0.05080	0.851	9	−0.2975	0.137
DGE-confirmed case rate^*b*^	1	0.696	−0.05188	0.855	9	−0.3013	0.133
*R*_*t*_ (RT–PCR cases)	1	0.090	−0.09733	0.426	7	−0.128	**0.031**
*R*_*t*_ (DGE cases)	1	0.128	−0.07739	0.466	7	−0.09860	0.052
Hospitalized cases	1	0.980	0.03013	0.785	9	−0.8545	0.611
Hospitalization rate^*b*^	1	0.979	0.000344	0.785	9	−0.009466	0.611
% hospitalized	2	0.824	0.07636	0.639	3	−4.411	**0.003**
Pneumonia cases	1	0.716	−0.2194	0.739	9	−0.07787	0.929
Pneumonia rate^*b*^	1	0.716	−0.002444	0.738	9	−0.000848	0.930
% with pneumonia	1	0.187	−0.9657	0.420	1	−1.554	0.072

Two analyses are reported for each combination. The formal *VAR-order* Toda–Yamamoto Granger test uses the lag order selected by the Akaike information criterion. The *calibrated-lag autoregressive model* asks whether the Cq value at the calibrated lag adds information beyond one lag of the outcome. The latter targets the study's data-calibrated lag but is not a formal Granger-causality test. Complete results, including HAC inference, the reverse direction, and the sensitivity analyses, are given in Supplementary Tables S4a, b. Stationarity tests are given in Supplementary Table S5.

^*a*^ The VAR-order results are reported at 4.6 observations per parameter, below the prespecified criterion of five, and are descriptive only. The calibrated-lag autoregressive models in this block met the criterion when assessed separately.

^*b*^ Per 100,000 inhabitants. The first Chihuahua wave, whose longest contiguous run is 12 weeks, admits no VAR of any order and is not included. Nominal *p*-values below 0.05 are shown in bold. A positive lag denotes that the Cq value precedes the outcome.

These results must be interpreted at the horizon tested. The information criterion selected an order of 1–2 weeks in every case, whereas the calibrated lags range from +1 to +9 weeks. The VAR-order results therefore do not establish the absence of an association at longer lags. At the 1- to 2-week horizon selected by AIC, the observed cross-correlations were close to zero for the Jalisco case and hospitalization series (*r* = −0.004 and −0.004 at +1 week). At their calibrated lag of +9 weeks, the corresponding correlations were *r* = −0.574 and −0.523. A VAR spanning 9 weeks would require 19 parameters for the 38 weekly observations in Jalisco and is not estimable.

The second analysis used the secondary calibrated-lag autoregressive model (Supplementary Table S4b). It asked whether Cq measured *k* weeks earlier added information beyond the outcome's recent past (Equation 5). All 24 coefficients were negative, the predicted direction. This concordance is descriptive because lag calibration favored negative associations.

Four coefficients had classical *p* < 0.05. In the second Chihuahua wave at lag +8, the estimates were −0.144 for *R*_*t*_ (RT–PCR cases) (*p* = 0.013) and −0.119 for *R*_*t*_ (DGE cases) (*p* = 0.028). In Jalisco, the estimate for *R*_*t*_ (RT–PCR cases) was −0.128 at +7 (*p* = 0.031). The estimate for the hospitalization proportion was −4.411 at +3 (*p* = 0.003). Only the two *R*_*t*_ (RT–PCR cases) estimates also had HAC *p* < 0.05 (*p* = 0.021 in Chihuahua and *p* = 0.048 in Jalisco). Two associations crossed the nominal threshold only with HAC inference. These were the pneumonia proportion in the second Chihuahua wave (classical *p* = 0.064, HAC *p* = 0.007) and *R*_*t*_ (DGE cases) in Jalisco (classical *p* = 0.052, HAC *p* = 0.027).

When two outcome lags were controlled rather than one, the Chihuahua *R*_*t*_ (RT–PCR cases) estimate remained nominally significant (*p* = 0.014). The Chihuahua *R*_*t*_ (DGE cases) estimate had *p* = 0.032 but HAC *p* = 0.059. No count, rate, or recorded-pneumonia metric had a nominal *p* < 0.05 in either state under this specification. Because the lag was calibrated in the same data, these models assess association at the selected lag. They do not provide an independent Granger-causality test.

The second-Chihuahua-wave *R*_*t*_ correlation at +8 weeks (*r* = −0.745) was not adjusted for a linear time trend in M3 or M4. Only 19 pairs were available, below the prespecified minimum of 20 for M3. It is therefore supported by M1, M2, prewhitening and the calibrated-lag autoregressive model, but not by a trend-adjusted regression and should not be interpreted as such.

The contrast between metric families follows from the autocorrelation of the outcomes (Supplementary Table S4a). In the VAR equations, the adjusted *R*^2^ for case counts, case rates, hospitalizations, and pneumonia counts ranged from 0.70 to 0.90. Thus, the previous week's value accounted for much of their variation, leaving little additional variation for another predictor. The corresponding values for *R*_*t*_ ranged from 0.06 to 0.47. The effective reproduction number, a ratio already normalized by case volume, does not carry the momentum of the epidemic curve, and the Cq signal remains detectable in that metric once the outcome's own history is controlled for.

Two additional analyses were performed, both in Supplementary Table S4a. First, the reverse-direction test for the hospitalization proportion in the second Chihuahua wave yielded a nominal *p* = 0.009. This model did not meet the prespecified estimability criterion and is reported descriptively. It therefore does not establish bidirectional temporal predictability. No other reverse-direction result met the nominal significance threshold. Second, as a sensitivity analysis, conventional Granger tests on the first-differenced series agreed with the Toda–Yamamoto results in 22 of 24 combinations. The two exceptions were the proportion hospitalized (*p* = 0.005) and the proportion with pneumonia (*p* = 0.028) in the second Chihuahua wave.

Three distinct analytical approaches produced a similar pattern: adjustment for a linear time trend (M3), adjustment for the full covariate set (M4), and adjustment for the outcome's own past in the calibrated-lag autoregressive model. These approaches use the same data and calibrated lags and therefore do not constitute independent confirmation. The association between weekly median Cq values and case counts, hospitalizations, and their rates is substantially attributable to a shared epidemic trend and to autocorrelation of the outcomes. The association with the effective reproduction number was the most consistent across the three approaches. Table 2 summarizes the formal Granger tests and the calibrated-lag autoregressive models. Complete results are given in Supplementary Tables S4a, b.

### 3.6 Individual-level Cq determinations

All 10,910 Cq determinations included in the weekly medians were from cases classified as confirmed in their corresponding surveillance records: 3,874 in Chihuahua and 7,036 in Jalisco. The individual analyses used the timing, clinical, and vaccination information recorded for those cases. Figure 5 shows their distribution by timing of collection, severity, and vaccination history.

**Figure 5:**
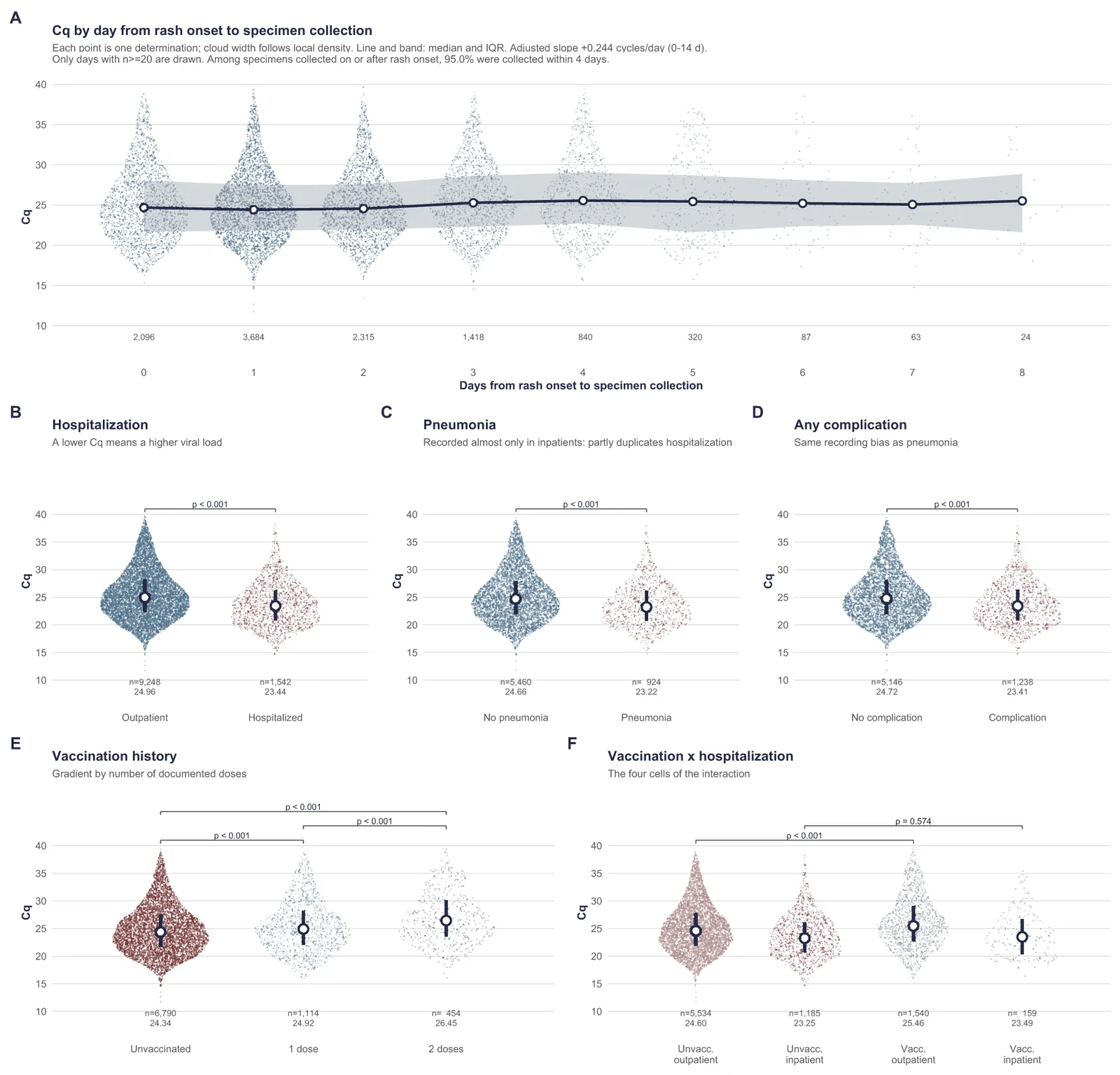
Individual Cq determinations by timing of specimen collection, severity and vaccination history. Each point is one of the 10,910 determinations analyzed. Cloud width is proportional to the local density of Cq values. Open circles mark the median, and vertical bars show the interquartile range. Group sizes and medians are printed beneath each group. **(A)** Interval in days from rash onset to specimen collection; **(B)** hospitalized against ambulatory cases; **(C)** recorded pneumonia against other recorded outcomes; **(D)** any recorded complication against none; **(E)** number of documented vaccine doses; **(F)** the four cells of vaccination status by hospitalization. A lower Cq is consistent with a higher measured viral RNA burden, so a group positioned lower on the axis had a higher measured RNA burden. Brackets are crude Wilcoxon rank-sum comparisons, Holm-adjusted within panels carrying more than one. This analysis is exploratory.

#### 3.6.1 Interval from rash onset to specimen collection

Figure 5A shows the distribution. Eight specimens (0.1%) were collected before rash onset and are reported separately. Among the remainder, Cq rose by 0.244 cycles per day of delay to collection after adjustment for age, sex and state (95% CI 0.190–0.299, *p* < 0.001, *n* = 10, 071). This direction was consistent with declining viral shedding after rash onset. The rise was not linear. The median Cq was 24.68 at rash onset, 24.41 on day 1 and 24.55 on day 2. It then stepped up to 25.28 on day 3 and 25.56 on day 4 before plateauing. Collection was concentrated early: 95.0% of specimens collected on or after rash onset were collected within 4 days. Across this early interval, the median changed by less than one cycle from its value at rash onset. This concentration does not rule out confounding by specimen timing.

#### 3.6.2 Age

The same adjusted model showed that Cq rose with age by 0.096 cycles per year (95% CI 0.091–0.102, *p* < 0.001, *n* = 10, 071). This was close to one cycle per decade. The gradient was similar in the dose-response model reported below (0.093 cycles per year, 95% CI 0.086–0.100, *p* < 0.001, *n* = 7, 576). The positive age gradient was therefore consistent across these two specifications.

#### 3.6.3 Severity

Hospitalized cases (Figure 5B) had lower Cq values than ambulatory cases. Their median was 23.44 (IQR 20.75–26.27, *n* = 1, 542), compared with 24.96 (IQR 22.20–28.26, *n* = 9, 248) in ambulatory cases. The crude difference was −1.52 cycles (*p* < 0.001). Adjustment for the collection interval, age, sex, and state attenuated but did not remove the difference (β = −1.021, 95% CI −1.287 to −0.756, *p* < 0.001). Cases with recorded pneumonia and with any recorded complication showed similar differences. The crude differences were −1.44 and −1.31 cycles, respectively, and the adjusted estimates were β = −1.008 and −0.920.

The pneumonia and complication results in Figures 5C, D are not independent of the hospitalization difference. The complications field was completed for 94.2% of hospitalized cases but only 52.9% of ambulatory cases. Recorded pneumonia therefore largely reflects pneumonia diagnosed during admission. Within hospitalized cases, where that asymmetry cannot operate, the difference between cases with and without pneumonia fell to −0.41 cycles. The median was 23.20 in cases with pneumonia (IQR 20.69–26.13, *n* = 890) and 23.61 in cases without pneumonia (IQR 20.96–26.59, *n* = 562). The difference was no longer significant crudely (*p* = 0.086) or after adjustment (β = −0.399, *p* = 0.103). Recorded pneumonia therefore carries little information about the Cq beyond hospitalization itself.

#### 3.6.4 Vaccination history

Observed Cq medians rose across the ordered zero-, one- and two-dose groups (Figure 5E). The median was 24.34 in unvaccinated cases (IQR 21.60–27.44, *n* = 6, 790). It was 24.92 with one dose (IQR 22.02–28.21, *n* = 1, 114) and 26.45 with two doses (IQR 23.52–30.14, *n* = 454). The Kruskal–Wallis statistic was χ^2^ = 95.8 (2 df, *p* < 0.001). All three pairwise contrasts remained significant after Holm correction (all adjusted *p* < 0.001). Dose was modeled as a linear trend across zero, one, and two doses, with adjustment for the collection interval, age, sex, and state. The gradient was 0.761 cycles per dose (95% CI 0.589–0.932, *p* < 0.001, *n* = 7, 576). The higher Cq values are consistent with a lower measured viral RNA burden in cases infected despite vaccination. They do not measure viable-virus excretion or infectiousness. Median age differed between strata (17, 15, and 26 years, respectively). Age was included in the adjustment, but residual confounding by vaccination cohort remains possible.

#### 3.6.5 Interactions

Five two-way interactions were examined on the adjusted scale (Table 3). Two interactions had nominal *p* < 0.05 in the pooled data: hospitalization by age (β = −0.058, *p* < 0.001) and vaccination by age (*p* = 0.041). These exploratory tests were not adjusted for multiple testing. The hospitalization-by-age estimate indicates that the Cq gap between hospitalized and ambulatory cases widens with age. The remaining three interactions did not meet this nominal threshold. The vaccination-by-collection-interval interaction was not significant (*p* = 0.229), providing no evidence that the association between collection timing and Cq differed by vaccination status. In a separate direct comparison, the adjusted collection interval changed by −0.029 days per dose (robust 95% CI −0.114 to 0.056, *p* = 0.501). There was no detectable systematic timing difference across the dose groups. This null result does not prove that differential timing is absent.

**Table 3:** Two-way interactions in the individual-level analysis of the Cq value.

Interaction	β (95% CI)	*p*-Value	*n*
Hospitalization × age	−0.058 (−0.079 to −0.038)	< 0.001	9,980
Vaccination × age	−0.018 (−0.034 to −0.001)	0.041	7,727
Vaccination × hospitalization	−0.495 (−1.278 to +0.288)	0.215	7,639
Hospitalization × collection day	+0.081 (−0.047 to +0.209)	0.214	9,980
Vaccination × collection day	−0.073 (−0.191 to +0.046)	0.229	7,727
Vaccination	Setting	Median Cq (IQR)	*n*
Unvaccinated	Ambulatory	24.60 (21.83–27.75)	5,534
Unvaccinated	Hospitalized	23.25 (20.72–26.13)	1,185
Vaccinated	Ambulatory	25.46 (22.66–29.12)	1,540
Vaccinated	Hospitalized	23.49 (20.32–26.74)	159

Each row is a separate linear model of the individual Cq on the two terms shown, their product, and the base adjustment set (interval from rash onset to specimen collection, age, sex, and state). The coefficient reported is the product term. A negative interaction coefficient means that the association of one variable with measured Cq becomes more negative as the other increases. The lower panel shows the same vaccination-by-hospitalization contrast expressed as observed medians in the four cells, which the product term does not resolve. This analysis is exploratory and no multiplicity correction was applied across the five interactions. Coefficients are in Cq cycles. For the interactions with age and with collection day, the coefficient is the change in the effect of the binary variable per additional year of age or per additional day to collection. Vaccination denotes a declared history of any measles vaccination. The dose-response gradient across zero, one and two documented doses is reported in the text. The vaccination effect is +0.86 cycles among ambulatory cases and +0.24 among hospitalized ones. The interaction term does not confirm this contrast, which rests on 159 determinations in the smallest cell.

Examined as four cells rather than as a product term, vaccination was associated with a higher Cq among ambulatory cases (24.60, *n* = 5, 534, against 25.46, *n* = 1, 540; +0.86 cycles). There was little difference among hospitalized cases (23.25, *n* = 1, 185, against 23.49, *n* = 159; +0.24 cycles). These two within-setting contrasts are shown in Figure 5F. The interaction term did not reach significance (β = −0.495, *p* = 0.215). The smallest cell contains 159 determinations, so this pattern is reported as a hypothesis.

#### 3.6.6 Sensitivity by state

In the state-specific analyses, the state term was dropped from the adjusted models (Supplementary Figure S1). The directions of the vaccine dose gradient, the hospitalization difference and the collection-interval association were consistent in both states. The raw medians increased across zero, one and two doses in both Chihuahua (23.80, 25.30, and 26.58) and Jalisco (24.67, 24.81, and 26.26). With spline adjustment for age, the formal dose-by-state interaction was significant (*p* = 0.006): the robust dose slope was 1.096 cycles in Chihuahua and 0.552 in Jalisco. Entering dose as a factor yielded adjusted contrasts of +0.673 cycles for one vs. zero doses and +1.602 for two vs. zero (joint *p* < 0.001). Thus, the result did not depend on a linear-dose or linear-age specification (Supplementary Table S11).

The hospitalization difference also held in both states (Chihuahua −1.293 cycles, *p* < 0.001; Jalisco −0.887, *p* < 0.001). The positive slope for the collection interval also held in both states (Chihuahua +0.221, Jalisco +0.258 cycles per day, both *p* < 0.001).

Other findings varied by state. Pneumonia restricted to hospitalized cases is significant in Chihuahua (−0.803 cycles; *p* = 0.015) but null and of opposite sign in Jalisco (+0.210; *p* = 0.56). The hospitalization-by-age interaction is confined to Chihuahua (−0.067, *p* < 0.001; Jalisco −0.016, *p* = 0.36), as is the vaccination-by-age interaction.

These individual-level comparisons are exploratory and were specified without prior hypotheses. No multiplicity correction was applied beyond the within-family Holm adjustment described in Section 2.6. Marginal results are therefore hypothesis-generating.

### 3.7 Out-of-sample predictive gain

Predictive performance depended on the evaluation framing (Section 2.6). The complete grid of 242 estimable configurations is reported in Supplementary Table S6. Under the nowcast framing with leak-free lag selection, only six of 69 configurations achieved positive skill, with a median of −0.280. Aggregate Cq rarely added value beyond a comparator that already included the previous week's outcome. Under the lead-time framing with the fixed calibrated lag, 32 of 54 configurations achieved positive skill. Re-selecting the lag within each training segment reduced this to 20 of 65 and moved the median from +0.080 to −0.036. Apparent gain was therefore sensitive to in-sample lag selection.

One analytic unit showed predictive gain across all variants. The outcome was the reproduction number derived from laboratory-positive cases in Chihuahua across both waves. Adding Cq reduced out-of-sample error by 55% under the nowcast framing with the fixed lag (88% of folds, *p* < 0.001). The reduction was 40% with the lag re-selected inside each fold (86% of folds, *p* < 0.001). Under the lead-time framing with leak-free selection, the reduction was 34% (82% of folds, *p* = 0.001).

Two features limit this finding. First, it concerns a single analytic unit. The Jalisco wave showed positive but never significant leak-free lead-time skill (maximum 20%, *p* = 0.064). Second, lag selection within each fold yielded 2 weeks rather than the eight calibrated in sample. The horizon showing predictive gain was therefore considerably shorter than the lead time estimated by cross-correlation. The complete grid is given in Supplementary Table S6.

## 4 Discussion

We analyzed two sequential measles outbreaks covering 10,910 Cq determinations and 11,735 confirmed cases. The selected-lag correlations between weekly median Cq and the reported epidemiological metric families were negative at leads of 1–9 weeks. Because calibration selected the most negative correlation at a positive lag, this directional concordance is descriptive. After accounting for linear time, specimen composition, and the outcome's own recent past, the most consistent remaining association was with the effective reproduction number rather than with epidemic counts.

This pattern was similar for *R*_*t*_ derived from laboratory-positive cases and *R*_*t*_ derived from final surveillance-confirmed cases. Their agreement shows that the direction did not depend on one operational case definition, but it is not independent confirmation. Both estimates arise from the same system, and one case population is nested within the other.

The three adjusted approaches address different explanations for the observed association. M3 attenuates a shared linear trend. M4 adds weekly specimen-composition covariates. The calibrated-lag autoregressive model controls the epidemic's recent momentum. In Jalisco, the only series supporting M4 had bootstrap intervals that excluded zero for both *R*_*t*_ estimates but not for severity proportions. The direction of the *R*_*t*_ estimates was also preserved in endpoint and composition sensitivities. Associations with case and hospitalization counts and their rates were substantially attenuated or lost. This contrast is consistent with their temporal structure: the adjusted *R*^2^ for the autoregressive equations was 0.70–0.90 for counts and rates, but 0.06–0.47 for *R*_*t*_. Counts already carry much of the preceding week's epidemic momentum, whereas *R*_*t*_ is normalized by case volume. Severity proportions are likewise normalized, but their uncertainty still depends on the weekly denominator. Their adjusted estimates remained negative while the primary bootstrap intervals crossed zero. These converging analyses use the same outbreaks and calibrated lags, thereby establishing robustness, not external validation.

Population-level Cq previously tracked transmission and outcomes across SARS-CoV-2 pandemic years (8). This study examines that premise in a pathogen with a shorter shedding window and much higher transmissibility, using rash-based surveillance and mandatory testing of suspected cases, while adding temporal-precedence and reverse-direction analyses. The contrast with demand-driven pandemic testing matters. When sampling is tied to a standard case definition and a narrow collection window, changes in who seeks testing should play a smaller role. Changes in case finding and specimen quality remain possible. The recurrence of a leading association under these conditions supports a signal of epidemic phase. It cannot, however, separate biology from every surveillance process. The underlying proposition—that cross-sectional viral-load distributions contain information about epidemic phase—was established for SARS-CoV-2 (5). A 16-study review found predominantly negative relations between Cq and epidemic trends (44). National influenza surveillance has also detected an early population-level Cq signal (9). The present results suggest, but do not establish, that this premise transfers to measles.

The proposed population mechanism is compositional rather than a claim that one person's Cq forecasts later cases. During epidemic growth, a weekly sample can include a larger share of recently infected people near the peak of measured RNA, lowering the median. During decline, relatively later infections can shift the distribution upward. Measles surveillance may accentuate this contrast because specimen collection is linked to rash onset, but the same weekly median can also move when age, vaccination, access to testing or collection timing changes. These compositional influences explain the need for adjusted models and individual-level checks. The remaining association is therefore a candidate surveillance signal rather than a direct measure of transmission.

The weekly median is meaningful only if its component measurements retain biological information. Individual Cq values increased across zero, one and two documented vaccine doses, and the association persisted with flexible age and state-specific specifications. The ordered relation was not driven solely by the older age of the two-dose group. It persisted when age was modeled with a spline and when dose was entered as a factor. Residual confounding by vaccination cohort remains possible. The vaccine dose gradient is consistent with lower measured viral RNA burden in breakthrough infections, not with a lower viable-virus excretion, infectiousness or secondary transmission. The direction aligns with higher Cq in nasopharyngeal specimens from vaccinated vs. naive cases (median 32 vs. 19). It also aligns with higher Cq in modified than in typical or prodromal measles and with lower viral burden in cases attributed to waning immunity (45–47). Those reports use different designs, assays and clinical definitions, so their agreement is qualitative. The individual analysis is an observational biological consistency check independent of the weekly lag calculation, not validation of the time-series result or proof of a universal dose response.

The agreement is not universal. A Japanese outbreak found about 100-fold lower genome copy numbers in blood from modified cases but no significant difference in throat swabs or urine (48), a direct counterexample to the specimen used here. Its small case series and the sub-cycle dose effect in this study may explain the contrast. Replication in independent throat-swab series remains necessary.

Other individual-level checks also inform interpretation. The hospitalization gradient was also directionally consistent in both states after adjustment. Collection was concentrated within 4 days of rash onset and did not differ detectably by dose. Specimen timing can nevertheless shift individual values and weekly composition. Residual confounding by timing cannot be excluded. These checks support the plausibility of the exposure while preserving the distinction between measured RNA, disease severity and transmission.

The individual-level analysis also argues against treating pneumonia as an independent severity marker. Recorded pneumonia in this system is largely pneumonia diagnosed during an admission. Among hospitalized cases, the difference in Cq disappears and its sign is inconsistent between the two states. Pneumonia and hospitalization therefore do not represent two independent findings. The clinical literature is compatible with this interpretation: in a multicenter cohort of children hospitalized with measles, pancreatitis and encephalitis were independent predictors of severe outcome. Pneumonia was not among the reported independent predictors (49).

Interpreting the association with severity also requires reference to the broader literature. Evidence on Cq and clinical severity is inconsistent, so the *p*-value alone does not establish how broadly this association applies. A meta-analysis of seven SARS-CoV-2 studies found no significant difference in mean Cq values between hospitalized and non-hospitalized patients (50). A systematic review of respiratory viruses found an association with severity in a minority of studies. For influenza, this occurred in one of three studies for mortality and three of nine for severe disease or intensive care admission (51). The hospitalization difference of about one cycle is therefore an internally consistent observation across two outbreaks of a different pathogen, not a resolution of that literature. The state-stratified analysis is similarly informative. Both age interactions had nominally significant results in the pooled data. In the state-specific analyses, they met the nominal significance threshold only in Chihuahua. Pooling alone would obscure this heterogeneity. The vaccine dose gradient, hospitalization difference and positive collection-interval association were directionally consistent in both states. All of this analysis is exploratory, comprising multiple comparisons without prior hypotheses, and supports rather than confirms the study's premise.

Population differences also constrain transferability. Independent evidence places the two series within a northern-to-western shift of the Mexican epidemic. A scan-statistic analysis of 8,205 cases identified the same sequence (2). An earlier review still found more than 90% of cases in Chihuahua (52). By the end of this study, Jalisco had accumulated a comparable burden, providing a second sustained series under the same national diagnostic framework. Jalisco also had an adult-shifted distribution, with the largest share among ages 20–49 years (53). Conversely, 90.7% of hospitalized patients in West Texas were younger than 18, and 79.7% of cases in Canada were unvaccinated (54, 55). These differences affect specimen composition, clinical presentation, and vaccine history. Because individual Cq increased with age here, the observed lags should not be assumed to transfer to a childhood-predominant outbreak.

The potential operational lead time also requires context. Wastewater surveillance provides a useful scale comparison: published signals have preceded clinical indicators by roughly 4–6 days or 1–2 weeks (56, 57). The 7- to 8-week calibrated *R*_*t*_ lag here is much longer. However, the correlation peaks are broad, and measles has a longer serial interval than COVID-19 (11.1 vs. 5.2 days). The reference outcome is also a smoothed reproduction-number estimate rather than notified cases (27, 28, 58, 59). The calibrated lag should therefore be read as “about 2 months,” not as a precise or validated warning horizon. Wastewater and Cq also occupy different points in the surveillance chain: one aggregates a community specimen before clinical contact, whereas the other summarizes specimens already collected from notified cases. Their lead times are therefore not directly exchangeable. A prospective analysis with the same target, reporting calendar and loss function is required before comparing operational value.

Operational feasibility is a separate consideration. Weekly median Cq uses a routine testing by-product without additional sampling or instrumentation. In this extract, the recorded laboratory-result date followed collection by a median of 3 days, and 94.3% were recorded within 7 days. Aggregation could therefore be technically timely. However, the file does not show when decision-makers could access or use the result. The strongest retrospective *R*_*t*_ associations occurred 7–8 weeks later, but the only leak-free predictive gain occurred in one analytic unit at a median 2-week horizon. The turnaround finding therefore addresses the feasibility of data production, not forecasting performance. A useful prospective system would also need a prespecified aggregation schedule, minimum weekly specimen count, alert threshold and response protocol.

For scale, modeling in Niamey suggested that action within 60 days, together with vaccination expanded to children aged 6 months–15 years, could avert up to 90% of cases (60). Current guidance aligns outbreak immunization within 15 days with the 7-1-7 framework (61). These comparisons do not show that Cq would advance a campaign. They support prospective evaluation of Cq beside the case curve as an ancillary signal, with actual data availability and decision thresholds measured in real time.

A prospective display would need to show the number of analyzable weekly determinations, the median and its uncertainty, Cq-field completeness, and the accompanying case-based indicators. A falling median should prompt assessment of concordant changes rather than automatic action. Predefined rules would also be needed for sparse weeks, laboratory changes and delayed results. This combined view preserves the advantages of routine Cq data without displacing case investigation, vaccination coverage or formal estimates of epidemic growth.

Several limitations bound the inference. First, lag calibration selected the most negative correlation among positive lags in the same data used to report it. This selective inference inflates the estimate (62). The cross-correlation stage is therefore calibration rather than testing, and +9 weeks should be read as roughly 2 months. The primary bootstrap held the selected k^*^ fixed and did not propagate uncertainty from lag calibration. Prewhitening retained the Jalisco correlations and the second-Chihuahua-wave *R*_*t*_ signal but attenuated the other Chihuahua associations. The pattern was therefore not uniform across series. The incomplete week beginning 27 July was excluded. Further endpoint exclusions preserved the *R*_*t*_ direction, but M4 became non-estimable after 4 weeks were removed. Likewise, setting *d*_max_ = 2 removed the nominal HAC result from the Jalisco formal Granger test. These sensitivities restrict the claim to a retrospective *R*_*t*_ association rather than a general leading signal across outcomes.

Second, precedence does not establish predictive value (63). Out-of-sample gain survived every variant in only one unit, at about 2 weeks rather than eight. This mixed result cannot replace a prospective design. The surviving outcome is itself an estimate, reconstructed under an assumed generation interval and a smoothing window (59). Using an estimated dependent variable without propagating its uncertainty can bias coefficients and reduce confidence-interval coverage (64). The findings therefore concern the sign and ordering of the coefficients, while their magnitudes are indicative. Power was also uneven. The first Chihuahua wave supported only the unadjusted model and was descriptive. The second fell below the VAR estimability criterion and was flagged, and its *R*_*t*_ regressions did not support M3 or M4. Only Jalisco supported the full covariate set. One of 24 reverse-direction tests yielded a nominal association between the hospitalization proportion and subsequent Cq in the second Chihuahua wave. That model fell below the prespecified estimability threshold and is reported descriptively. It does not establish bidirectional temporal predictability.

Third, measurement and recording limit the interpretation of Cq and severity. Completeness of the complications field changed from absent to complete and depended on hospital contact. Pneumonia is therefore secondary, whereas hospitalization was recorded for 99.0% of cases. Cq remains a proxy. The national algorithm standardizes the target and thermal profile, but no study-specific calibration established numerical interchangeability between Chihuahua and Jalisco. Primary time-series analyses therefore treated the states as parallel within-state series. Pooled individual models adjusted for state, but residual laboratory differences remain possible. The collection interval was bounded here, but specimen quality was not and can shift Cq measurably (65). Cq measurements may also be non-transferable across assay systems (50). These sources of variation preclude interpreting Cq as an absolute copy number.

Finally, surveillance coverage and population selection limit generalization. Surveillance data exclude cases that are never notified, and measles under-ascertainment has been estimated at two- to almost fivefold (66). This would not necessarily shift the weekly median if notification and testing were independent of Cq and its determinants. Differential ascertainment by severity, vaccination status, or collection timing could bias the median. Under-ascertainment also affects every metric derived from the case curve. Two states and three series form a narrow base selected *a priori* for sustained transmission. The relationship was observed only under that condition and should not be assumed to transfer. Sex was included as a covariate but could not be analyzed in separate weekly series because stratification would leave weeks without a median. This limitation is reported in accordance with SAGER (67). The system records no information on gender.

These limitations define the priorities for further evaluation. Turning precedence into prediction requires a prospective evaluation with a comparator, a held-out period and a loss function fixed in advance. It should be powered around the reproduction number rather than case counts. Replication is needed in outbreaks with a younger age structure, since individual Cq rises with age. Independent throat-specimen series are also needed because the published dose-gradient comparison found no difference (48). Longer series would allow sex-specific weekly medians, as recommended by SAGER (67).

Two developments would extend the use of aggregate Cq. Per-run controls reported alongside routine results would support comparison across laboratories rather than only within one assay. Its added value should also be tested within the nowcasting frameworks already used to correct for reporting delay. Prospective work should also record three timestamps separately: specimen collection, laboratory result generation and availability to the surveillance team. That distinction would establish whether an analytically useful signal is available early enough to influence a defined public-health decision.

## 5 Conclusions

In an analysis of two measles outbreaks, the weekly median RT–PCR Cq of notified cases preceded the epidemiological metrics of the epidemic in the direction predicted by the hypothesis. This temporal ordering is descriptive because lags were calibrated using the same data. After adjustment for a linear time trend, specimen composition and the outcome's own past, the most consistent association was with the effective reproduction number. Associations with case and hospitalization counts were attenuated, and the severity-proportion estimates remained uncertain. Weekly aggregate Cq can be calculated from measurements already recorded during case-level surveillance testing. Its calculation requires no additional specimen, instrument, or field procedure. These results support evaluating it as an ancillary early indicator alongside the case curve, using the reproduction number rather than case or hospitalization counts as the target. Predictive gain with leak-free lag selection was limited to one analytic unit and occurred at a shorter horizon than the retrospective association. Whether aggregate Cq can support operational decisions remains to be determined prospectively.

## Data Availability

The de-identified case-level EFE/SINAVE data analyzed here were obtained through Mexico's national framework for transparency and access to public information. Any investigator can request them through the same procedure. The reduced EFE data set published by the General Directorate of Epidemiology can be downloaded without restriction and was used only for aggregate verification (22). Individual-level records are not redistributed by the authors, because they derive from name-based epidemiological surveillance. The aggregated weekly series and analysis code corresponding to the complete-week analysis reported here are publicly available in Zenodo, version 2.1.0, at https://doi.org/10.5281/zenodo.21915025.
