# Supplementary material for: Population-level measles RT–PCR cycle threshold values and their relationships with disease severity, transmission, and incidence metrics: a time series analysis of the 2025–2026 outbreak in Mexico
Source: Front Public Health. 2026 Oct 8;14:1967103. doi: 10.3389/fpubh.2026.1967103 (PMC13687380; doi:10.3389/fpubh.2026.1967103)
Supplement: Supplementary file 1 [file Data_Sheet_1.pdf]

frontiers

Supplementary Material

Contents

**Figure S1**	Individual Cq determinations stratified by state	2
**Supplementary Methods**	Detailed statistical implementation and sensitivity specifications	2
**Table S0a**	Source variables and analytic derivations	4
**Table S0b**	Outcome codes used throughout the supplementary tables	6
**Table S1a**	Cross-correlation between the weekly median Cq value and each metric, at every lag from -10 to $+10$ weeks. Chihuahua, both waves	7
**Table S1b**	Cross-correlation between the weekly median Cq value and each metric, at every lag from -10 to $+10$ weeks. Jalisco	9
**Table S1c**	Prewhitened cross-correlation sensitivity analysis	10
**Table S2a**	Lagged regression at the calibrated lag under four nested specifications: point estimates and moving-block bootstrap intervals	12
**Table S2b**	Lagged regression, continued: classical and Newey–West HAC sensitivity inference	17
**Table S3**	Calibrated Cq lag for each metric and analytic series, with the rule used to assign it	22
**Table S4a**	Formal Granger causality by the Toda–Yamamoto procedure and vector autoregression, at the VAR lag order selected by AIC	24
**Table S4b**	Calibrated-lag autoregressive association models	26
**Table S5**	Augmented Dickey–Fuller and KPSS stationarity tests, in levels and in first differences	28
**Table S6a**	Out-of-sample evaluation: complete grid of analytic configurations and fold counts	30
**Table S6b**	Out-of-sample evaluation, continued: predictive performance for the same configurations	38
**Table S7**	STROBE checklist, with the location of each item in this article	46
**Table S8**	RECORD checklist for routinely collected health data, with the location of each item in this article	47
**Table S9**	Sensitivity to excluding one, two or four endpoint weeks in Jalisco	49
**Table S10**	Jalisco composition-adjustment sensitivity models	50
**Table S11**	Vaccine-dose model sensitivities and state interaction	51
**Table S12a**	Unsplit Chihuahua cross-correlation sensitivity	52
**Table S12b**	Jalisco Toda–Yamamoto sensitivity with $d_{\max} = 2$	53
**Table S13**	Operational timeliness from specimen collection to recorded Cq result	54

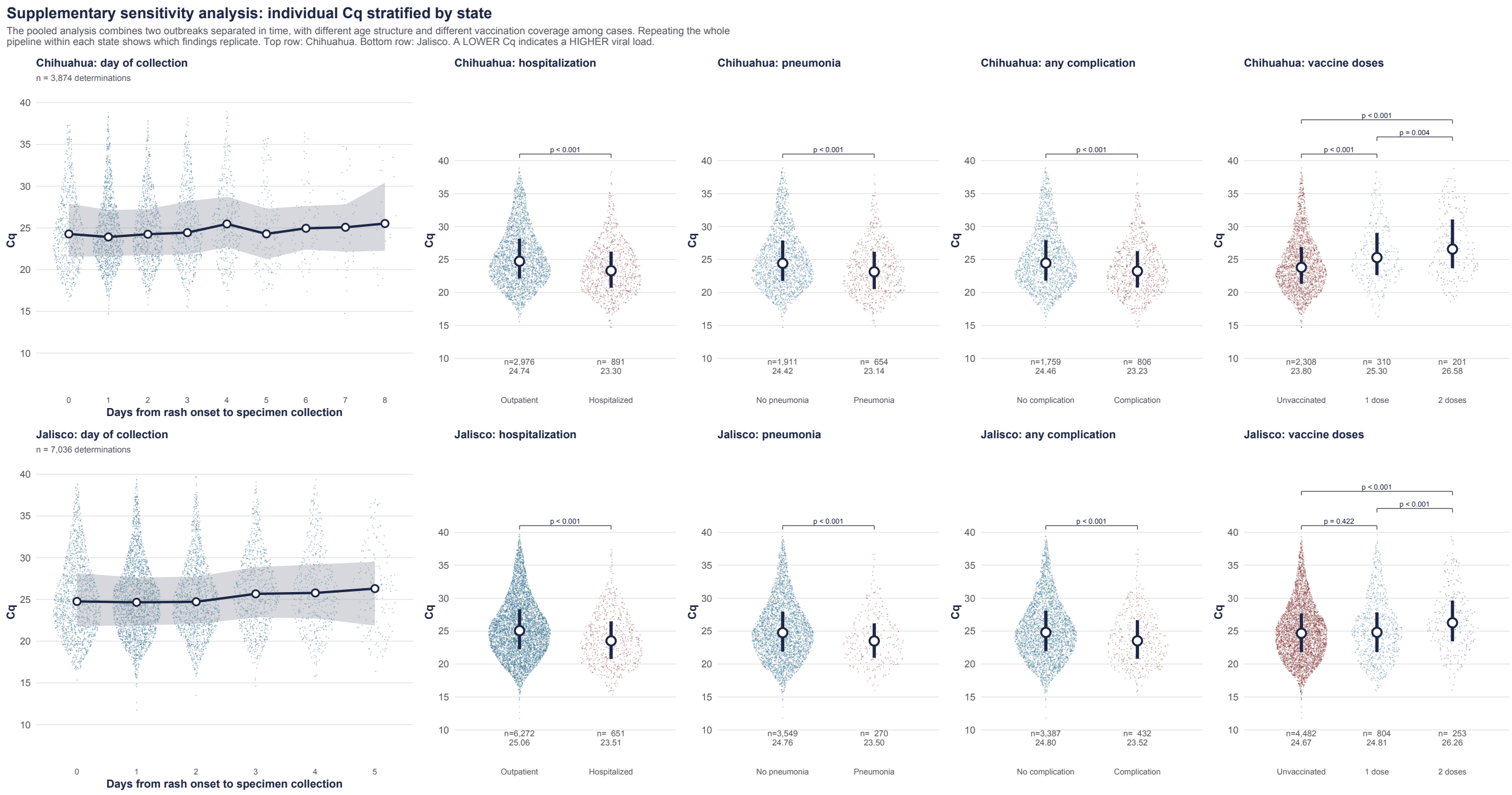

Figure S1. Individual Cq determinations stratified by state.

SUPPLEMENTARY METHODS

Assay framework

The national measles real-time RT–PCR framework specified primers MVN1139-F and MVN1213-R, the FAM-labeled probe MVNP1163-P, and human RNase P as the internal control. Reverse transcription was performed at 48 °C for 30 minutes, followed by 40 amplification cycles. These specifications describe the shared national assay framework under which the state laboratories operated. No study-specific interlaboratory calibration panel was available.

Cross-correlation and prewhitening

The complete cross-correlation function was calculated over lags from -10 to $+10$ weeks. Positive lags were interpreted as Cq preceding the outcome. The calibrated lag used in subsequent association models was the positive lag with the most negative correlation, subject to the minimum-pair rule stated in the main Methods. This was an association-calibration step rather than a test of causality.

For prewhitening, missing weekly Cq medians within an otherwise contiguous series were linearly interpolated only to fit the filter. An autoregressive integrated moving average model was selected with maximum $p = q = 3$, maximum $d = 1$, no seasonal terms, mean or drift, and corrected Akaike information criterion. The selected Cq filter was applied to the outcome before the residual cross-correlation was recalculated. Raw and prewhitened results are both retained because filtering can reduce common-trend correlation but cannot create independent replication.

Lagged regression and time-series inference

The four nested specifications (M1–M4) are defined in the main Methods. Primary confidence intervals were obtained with a moving-block bootstrap of 2,000 replicates using seed 20260803 and a block length

of four weeks. Classical ordinary-least-squares and Newey–West heteroskedasticity- and autocorrelation-consistent inference, with lag $\lfloor 4(n/100)^{2/9} \rfloor$, were retained as sensitivities. A model was declared estimable when it had at least five complete observations per fitted slope parameter, excluding the intercept. Endpoint sensitivities removed one, two or four terminal Jalisco weeks. Composition sensitivities added vaccination composition, the median onset-to-collection interval or the percentage of candidate records without an analyzable Cq value. The unsplit Chihuahua analysis and the Jalisco $d_{\max} = 2$ Toda–Yamamoto analysis used the same definitions as their primary counterparts.

Stationarity, Granger analysis and predictive evaluation

Augmented Dickey–Fuller tests used a constant and lag order selected by the Schwarz criterion. Kwiatkowski–Phillips–Schmidt–Shin tests used a constant and the default short-lag bandwidth. A series was considered stationary only when both tests supported stationarity. Toda–Yamamoto models were fitted in levels with $p + d_{\max}$ lags, where p was selected by Akaike information criterion and $d_{\max} = 1$ in the primary analysis. Wald restrictions were applied only to the first p Cq lags.

The out-of-sample analysis evaluated the complete grid of 242 configurations shown in Tables S6a and S6b, combining analysis unit, outcome, model family, comparator and lag-selection rule. Rolling origins respected temporal order. Lag selection was repeated inside each training fold when the configuration required it. Performance was interpreted as incremental prediction error relative to the comparator, not as validation of a forecasting system or proof of causal direction.

Table S0a. Source variables and analytic derivations.

Information used	Source variable(s)	Analytic definition or derivation
Surveillance record identifier	EFES_ID	Screened for repeated case records. No repeated identifier was found among the 21,390 Chihuahua and Jalisco records.
State	DES_EDO	The analysis was restricted to Chihuahua and Jalisco. State defined the analytic series and entered pooled individual-level models as an adjustment term.
Rash-onset date	FEC_INI_EXANT	The field was converted to a calendar date and used for confirmed-case eligibility, state wave windows, epidemiological week of cases and clinical outcomes, and the onset-to-collection interval.
Specimen-collection date	FEC_TOMA_LESP3	The field was converted to a calendar date and used to assign Cq determinations to epidemiological week and to calculate days from rash onset to collection. Cq determinations required both rash onset and specimen collection to fall within the corresponding complete state-wave window.
Laboratory-result date	FEC_RESUL_LESP3	Converted to a calendar date and compared with specimen collection to describe operational timeliness among the analytic Cq determinations. Intervals with a result date before collection were retained in the completeness count but excluded from interval summaries. This date is a proxy for laboratory-result generation and does not establish when the result became available to, or was used by, decision-makers.
Measles throat-swab laboratory block	DES_PADECI3, DES_TIP_MUESTRA3	Required disease equal to SARAMPION and specimen equal to EXUDADO FARINGEO.
Measles Cq	RESULTADO_CORTE3	The field was converted to numeric. Values greater than zero and below 40 cycles in the measles throat-swab block were considered analyzable. This was a data-cleaning rule, not a case-classification criterion. Of 18,297 eligible measles throat-swab laboratory blocks, 4,943 lacked a numeric Cq value and 2,148 had the Cq field recorded as 0. The source extract does not establish the laboratory, administrative or data-entry mechanism represented by a recorded zero. Of the 11,206 non-zero numeric Cq-field values, two implausible raw entries (2016 and 3078) were excluded, leaving 11,204 analyzable values satisfying $0<\mathrm{Cq}<40$. The weekly predictor was the median of at least five analyzable determinations.
Positive RT-PCR result	DES_RESULTADO3	Laboratory-positive records were identified by POSITIVO in the measles throat-swab block. These records were aggregated by rash-onset week for laboratory-positive counts, rates and the corresponding reproduction-number series.

continues on the next page

Table S0a, continued

Information used	Source variable(s)	Analytic definition or derivation
Final measles classification	CLAS_FINAL_SARAMPION	CONFIRMADO defined the confirmed-case population. DESCARTADO and records still under investigation were excluded from that population.
Hospitalization	HOSP	Code 1 was yes and code 2 was no. Codes 0 and 9 and missing values were treated as unavailable. The field was used for weekly severity measures and individual Cq contrasts.
Hospital-admission date	FEC_HOSP	Converted to a calendar date and used to summarize the interval from rash onset to admission among hospitalized cases with a recorded date.
Pneumonia and any complication	CVE_COMPLIC, DES_COMPLIC	For weekly recorded-pneumonia metrics, records containing the description NEUMONÍA were counted as events. Records without a documented pneumonia code contributed zero. These variables therefore represent recorded pneumonia rather than complete clinical ascertainment of pneumonia. For individual contrasts, code 2 defined pneumonia, and codes 1 and 3 to 6 formed the recorded non-pneumonia comparison. Codes 1 to 5 defined any complication, and code 6 defined none. Unavailable codes were excluded from individual contrasts.
Death	DES_EVOLU	Description DEFUNCIÓN identified deaths reported in the descriptive flowchart.
Measles vaccination and documented doses	CTA_VAC_SAR, CTAS_DOS	Vaccination code 1 was yes and code 2 was no. Other values were unavailable. Dose-response analyses compared unvaccinated cases with cases having one or two documented doses. Other or undocumented dose counts were excluded. Sensitivities modeled dose as a factor, age flexibly with restricted cubic splines, and a dose-by-state interaction.
Age	IDE_EDA_ANO, IDE_EDA_MES, IDE_EDA_DIA	Completed years were used in the two-state weekly and individual analyses. Months and days were used only to place infants in the national descriptive age profile.
Reported sex	IDE_SEX	Code 2 was women and code 1 was men. Other values were unavailable. The field was used to calculate the weekly percentage of women and as an individual-level adjustment term.
Indigenous status	ES_INDIGENA	Code 1 denoted Indigenous status and code 2 non-Indigenous status. The weekly denominator comprised records coded 1 or 2 among analyzable Cq determinations. No other or missing values occurred in this subset. The percentage of code-1 records entered the fullest adjusted time-series model.

Table S0b. Outcome codes used throughout the supplementary tables.

Code	Outcome
casos_dge	DGE-confirmed cases
casos_lab	Positive RT-PCR cases (state laboratory)
hosp_n	Hospitalized cases
hosp_prop	% hospitalized
hosp_tasa	Hospitalization rate per 100,000
neum_n	Pneumonia (cases)
neum_prop	% with pneumonia
neum_tasa	Pneumonia rate per 100,000
rt_dge	Rt from DGE-confirmed cases
rt_lab	Rt from RT-PCR positive cases
tasa_dge	DGE-confirmed case rate per 100,000
tasa_lab	RT-PCR case rate per 100,000

Table S1a. Cross-correlation coefficients between the weekly median Cq value and each epidemiological metric, at every lag from −10 to +10 weeks. Chihuahua, both waves.

Outcome	Cq lag (weeks)																				
	**-10**	**-9**	**-8**	**-7**	**-6**	**-5**	**-4**	**-3**	**-2**	**-1**	**+0**	**+1**	**+2**	**+3**	**+4**	**+5**	**+6**	**+7**	**+8**	**+9**	**+10**
Chih. w1																					
n	7	8	9	10	11	12	12	13	13	13	13	12	11	10	9	8	7	6	5	4	3
WN ref.		.693	.653	.620	.591	.566	.566	.544	.544	.544	.544	.566	.591	.620	.653	.693					
casos_dge		−**.717**	−.499	−.170	.230	.278	.486	**.634**	**.714**	**.796**	**.687**	.467	.094	−.025	−.284	−**.855**					
casos_lab		−.674	−.531	−.154	.193	.236	.453	**.574**	**.677**	**.789**	**.708**	**.574**	.195	.200	−.183	−**.774**					
hosp_n		−.617	−.378	−.006	.479	.566	**.619**	**.685**	**.614**	.513	.186	−.074	−.239	−.226	.156	−.002					
hosp_prop		.509	.222	.395	.511	−.308	−.010	−.240	−**.662**	−**.725**	−**.615**	−**.625**	−.400	−.283	.196	.527					
hosp_tasa		−.617	−.378	−.006	.479	.566	**.619**	**.685**	**.614**	.513	.186	−.074	−.239	−.226	.156	−.002					
neum_n		−.565	−.515	.017	.427	.495	.548	**.662**	**.688**	.490	.310	.081	−.446	−.054	−.007	−.253					
neum_prop		.512	.111	.332	.564	−.134	−.131	.032	−.495	−**.854**	−.326	−**.578**	−.494	−.176	.181	.240					
neum_tasa		−.565	−.515	.017	.427	.495	.548	**.662**	**.688**	.490	.310	.081	−.446	−.054	−.007	−.253					
rt_dge		−.189	−.524	−.429	−**.774**	−.273	−.064	−.462	−.201	−**.613**	−**.626**	−**.664**	−**.797**	−.538	−.415	−.503					
rt_lab		.406	−.194	−.442	−.318	−**.821**	−.322	−.197	−.323	−.466	−**.662**	−**.670**	−**.703**	−.550	−.423	−.555					
tasa_dge		−**.717**	−.499	−.170	.230	.278	.486	**.634**	**.714**	**.796**	**.687**	.467	.094	−.025	−.284	−**.855**					
tasa_lab		−.674	−.531	−.154	.193	.236	.453	**.574**	**.677**	**.789**	**.708**	**.574**	.195	.200	−.183	−**.774**					
Chih. w2																					
n	16	17	18	19	20	21	22	23	24	25	26	25	25	24	23	22	21	20	19	18	17
WN ref.	.490	.475	.462	.450	.438	.428	.418	.409	.400	.392	.384	.392	.392	.400	.409	.418	.428	.438	.450	.462	.475
casos_dge	−.318	−.291	−.329	−.257	−.284	−.295	−.308	−.283	−.316	−.288	−.307	−.267	−.220	−.187	−.057	−.085	−.121	−.116	−.256	−.431	−.408
casos_lab	−.300	−.289	−.336	−.238	−.270	−.281	−.297	−.269	−.308	−.281	−.307	−.264	−.220	−.191	−.065	−.094	−.119	−.126	−.258	−.420	−.397
hosp_n	−.397	−.129	−.448	−.254	−.190	−.270	−.239	−.211	−.308	−.238	−.304	−.268	−.191	−.196	−.049	−.126	−.126	−.103	−.291	−.340	−.353
hosp_prop	−.249	.323	−.182	.231	.246	.323	**.631**	−.037	.041	**.517**	.052	−.261	**.422**	.199	−.217	.096	.115	−.222	−.162	**.555**	−.085
hosp_tasa	−.397	−.129	−.448	−.254	−.190	−.270	−.239	−.211	−.308	−.238	−.304	−.268	−.191	−.196	−.049	−.126	−.126	−.103	−.291	−.340	−.353
neum_n	−.366	−.161	−.429	−.209	−.173	−.282	−.215	−.228	−.316	−.239	−.297	−.257	−.183	−.175	−.058	−.122	−.101	−.096	−.310	−.302	−.408
neum_prop	−.129	.168	−.354	.305	−.028	−.015	.399	−.294	−**.484**	−.077	.009	−**.439**	.174	**.424**	−.006	−.195	**.471**	−.200	−.363	**.604**	−.407
neum_tasa	−.366	−.161	−.429	−.209	−.173	−.282	−.215	−.228	−.316	−.239	−.297	−.257	−.183	−.175	−.058	−.122	−.101	−.096	−.310	−.302	−.408
rt_dge	−.032	.168	−.373	−.234	−.031	−.401	−.265	−.019	−.101	−.178	.206	−.269	−.081	**.408**	.088	−.343	.320	−.288	−**.745**	**.477**	−**.481**
rt_lab	−.060	.196	−.353	−.248	.020	−.290	−.310	.003	−.015	−.216	.151	−.192	−.146	.262	.166	−.394	.277	−.186	−**.745**	.409	−.341
tasa_dge	−.318	−.291	−.329	−.257	−.284	−.295	−.308	−.283	−.316	−.288	−.307	−.267	−.220	−.187	−.057	−.085	−.121	−.116	−.256	−.431	−.408

tasa_lab	−.300	−.289	−.336	−.238	−.270	−.281	−.297	−.269	−.308	−.281	−.307	−.264	−.220	−.191	−.065	−.094	−.119	−.126	−.258	−.420	−.397

Cells are cross-correlation coefficients $r(k)$. Those whose absolute value exceeds the conventional white-noise reference band are shown in **bold**. n is the number of paired weeks, and WN ref. is $\pm 1.96/\sqrt{n}$. Both depend only on the series and lag, not on the outcome. They are therefore reported once per column rather than once per cell. Exact p-values are a deterministic function of r and n and are given in the accompanying data files. Coefficients are shown to three decimal places and without the leading zero here. The data files carry four decimal places. No correction for multiplicity is applied. Outcome codes are defined in Table S0b.

Table S1b. Cross-correlation coefficients between the weekly median Cq value and each epidemiological metric, at every lag from -10 to $+10$ weeks. Jalisco.

**Outcome**	**Cq lag (weeks)**																				
	**-10**	**-9**	**-8**	**-7**	**-6**	**-5**	**-4**	**-3**	**-2**	**-1**	**+0**	**+1**	**+2**	**+3**	**+4**	**+5**	**+6**	**+7**	**+8**	**+9**	**+10**
Jalisco																					
n	28	29	30	31	32	33	34	35	36	37	38	37	36	35	34	33	32	31	30	29	28
WN ref.	.370	.364	.358	.352	.346	.341	.336	.331	.327	.322	.318	.322	.327	.331	.336	.341	.346	.352	.358	.364	.370
casos_dge	**.382**	**.408**	.344	.314	.233	.192	.139	.099	.090	.083	.056	−.004	.001	.001	−.185	−.233	−.325	−**.448**	−**.445**	−**.574**	−**.459**
casos_lab	**.384**	**.409**	.343	.310	.228	.189	.137	.099	.093	.089	.063	.003	.006	.005	−.180	−.227	−.320	−**.451**	−**.446**	−**.575**	−**.461**
hosp_n	.291	.306	.315	.229	.204	.132	.158	.087	.121	.117	.030	−.004	−.089	−.111	−.277	−.336	−**.380**	−**.435**	−**.472**	−**.523**	−**.440**
hosp_prop	−.227	−.179	−.055	−.247	.042	−.281	−.015	−.051	.008	−.051	.007	−.077	.133	−**.469**	−.126	−.262	−.246	−.049	.033	.312	**.476**
hosp_tasa	.290	.306	.315	.229	.204	.131	.159	.086	.120	.116	.027	−.005	−.090	−.112	−.276	−.337	−**.381**	−**.435**	−**.471**	−**.523**	−**.440**
neum_n	.290	.179	.188	.204	.139	.195	.194	.106	.130	.104	.042	−.037	−.128	−.181	−.292	−**.381**	−.334	−.328	−**.443**	−**.457**	−**.379**
neum_prop	−.066	−.207	−.093	.089	.037	.083	.063	.119	.024	−.078	.179	−**.339**	−.019	−.318	−.201	−.179	−.058	.302	.306	.333	**.431**
neum_tasa	.289	.179	.187	.204	.139	.196	.194	.106	.129	.104	.040	−.038	−.129	−.181	−.291	−**.381**	−.335	−.328	−**.443**	−**.457**	−**.379**
rt_dge	−.025	−.080	−.007	−.197	−.180	−.224	−.210	−.047	−.217	−.095	.031	−.232	−.184	−.107	−**.376**	−.332	−.312	−**.464**	−.280	−.151	−.210
rt_lab	−.034	−.088	−.014	−.170	−.173	−.220	−.216	−.053	−.180	−.068	.034	−.238	−.193	−.114	−**.387**	−.281	−.261	−**.460**	−.280	−.124	−.201
tasa_dge	**.383**	**.409**	.344	.314	.233	.192	.139	.099	.089	.083	.055	−.006	−.000	.001	−.185	−.233	−.326	−**.448**	−**.445**	−**.574**	−**.459**
tasa_lab	**.384**	**.410**	.343	.310	.228	.189	.137	.098	.092	.088	.062	.002	.005	.005	−.180	−.227	−.320	−**.451**	−**.446**	−**.575**	−**.461**

Cells are cross-correlation coefficients $r(k)$. Those whose absolute value exceeds the conventional white-noise reference band are shown in **bold**. n is the number of paired weeks, and WN ref. is $\pm 1.96/\sqrt{n}$. Both depend only on the series and lag, not on the outcome. They are therefore reported once per column rather than once per cell. Exact p-values are a deterministic function of r and n and are given in the accompanying data files. Coefficients are shown to three decimal places and without the leading zero here. The data files carry four decimal places. No correction for multiplicity is applied. Outcome codes are defined in Table S0b.

Table S1c. Prewhitened cross-correlation sensitivity analysis.

Series	Outcome	Raw lag	Raw r	ARIMA	Cq interp.	PW r	PW p	PW n	WN ref.	PW best lag	PW min. r
Chih. w1	casos_lab	5	−0.774	ARIMA(0,1,1)	0.071	−0.526	0.145	9	0.653	5	−0.526
Chih. w1	casos_dge	5	−0.855	ARIMA(0,1,1)	0.071	−0.531	0.141	9	0.653	5	−0.531
Chih. w1	tasa_lab	5	−0.774	ARIMA(0,1,1)	0.071	−0.526	0.145	9	0.653	5	−0.526
Chih. w1	tasa_dge	5	−0.855	ARIMA(0,1,1)	0.071	−0.531	0.141	9	0.653	5	−0.531
Chih. w1	rt_lab	2	−0.703	ARIMA(0,1,1)	0.071	−0.203	0.527	12	0.566	1	−0.285
Chih. w1	rt_dge	2	−0.797	ARIMA(0,1,1)	0.071	−0.445	0.147	12	0.566	2	−0.445
Chih. w1	hosp_n	5	−0.002	ARIMA(0,1,1)	0.071	0.162	0.678	9	0.653	1	−0.307
Chih. w1	hosp_tasa	5	−0.002	ARIMA(0,1,1)	0.071	0.162	0.678	9	0.653	1	−0.307
Chih. w1	hosp_prop	1	−0.624	ARIMA(0,1,1)	0.071	−0.05	0.872	13	0.544	1	−0.05
Chih. w1	neum_n	5	−0.253	ARIMA(0,1,1)	0.071	0.02	0.958	9	0.653	2	−0.585
Chih. w1	neum_tasa	5	−0.253	ARIMA(0,1,1)	0.071	0.02	0.958	9	0.653	2	−0.585
Chih. w1	neum_prop	1	−0.578	ARIMA(0,1,1)	0.071	0.061	0.842	13	0.544	2	−0.198
Chih. w2	casos_lab	9	−0.42	ARIMA(2,1,0)	0.037	−0.143	0.571	18	0.462	10	−0.336
Chih. w2	casos_dge	9	−0.431	ARIMA(2,1,0)	0.037	−0.154	0.542	18	0.462	10	−0.421
Chih. w2	tasa_lab	9	−0.42	ARIMA(2,1,0)	0.037	−0.143	0.571	18	0.462	10	−0.336
Chih. w2	tasa_dge	9	−0.431	ARIMA(2,1,0)	0.037	−0.154	0.542	18	0.462	10	−0.421
Chih. w2	rt_lab	8	−0.745	ARIMA(2,1,0)	0.037	−0.644	0.003	19	0.45	8	−0.644
Chih. w2	rt_dge	8	−0.745	ARIMA(2,1,0)	0.037	−0.682	0.001	19	0.45	8	−0.682
Chih. w2	hosp_n	9	−0.339	ARIMA(2,1,0)	0.037	0.073	0.773	18	0.462	5	−0.336
Chih. w2	hosp_tasa	9	−0.339	ARIMA(2,1,0)	0.037	0.073	0.773	18	0.462	5	−0.336
Chih. w2	hosp_prop	1	−0.261	ARIMA(2,1,0)	0.037	−0.115	0.576	26	0.384	8	−0.453
Chih. w2	neum_n	9	−0.302	ARIMA(2,1,0)	0.037	0.111	0.66	18	0.462	10	−0.421
Chih. w2	neum_tasa	9	−0.302	ARIMA(2,1,0)	0.037	0.111	0.66	18	0.462	10	−0.421
Chih. w2	neum_prop	1	−0.439	ARIMA(2,1,0)	0.037	0.005	0.982	26	0.384	8	−0.348
Jalisco	casos_lab	9	−0.575	ARIMA(0,0,0)	0	−0.575	0.001	29	0.364	9	−0.575
Jalisco	casos_dge	9	−0.574	ARIMA(0,0,0)	0	−0.574	0.001	29	0.364	9	−0.574
Jalisco	tasa_lab	9	−0.575	ARIMA(0,0,0)	0	−0.575	0.001	29	0.364	9	−0.575
Jalisco	tasa_dge	9	−0.574	ARIMA(0,0,0)	0	−0.574	0.001	29	0.364	9	−0.574
Jalisco	rt_lab	7	−0.46	ARIMA(0,0,0)	0	−0.46	0.009	31	0.352	7	−0.46
Jalisco	rt_dge	7	−0.464	ARIMA(0,0,0)	0	−0.464	0.009	31	0.352	7	−0.464
Jalisco	hosp_n	9	−0.523	ARIMA(0,0,0)	0	−0.523	0.004	29	0.364	9	−0.523

continues on the next page

Table S1c, continued

**Series**	**Outcome**	**Raw lag**	**Raw r**	**ARIMA**	**Cq interp.**	**PW r**	**PW p**	**PW n**	**WN ref.**	**PW best lag**	**PW min. r**
Jalisco	hosp_tasa	9	−0.523	ARIMA(0,0,0)	0	−0.523	0.004	29	0.364	9	−0.523
Jalisco	hosp_prop	3	−0.469	ARIMA(0,0,0)	0	−0.469	0.004	35	0.331	3	−0.469
Jalisco	neum_n	9	−0.457	ARIMA(0,0,0)	0	−0.457	0.013	29	0.364	9	−0.457
Jalisco	neum_tasa	9	−0.457	ARIMA(0,0,0)	0	−0.457	0.013	29	0.364	9	−0.457
Jalisco	neum_prop	1	−0.339	ARIMA(0,0,0)	0	−0.339	0.04	37	0.322	1	−0.339

Series: Chih. w1, Chihuahua wave 1 (February–May 2025); Chih. w2, Chihuahua wave 2 (May–November 2025); Jalisco, Jalisco single wave (November 2025–July 2026). Outcome codes are defined in Table S0b. An ARIMA model was selected by corrected AIC for the centered weekly Cq series. The same fitted filter was then applied to each outcome before residual cross-correlation. PW r is the prewhitened correlation evaluated at the raw data-calibrated lag. PW best lag and PW min. r describe the most negative prewhitened correlation over positive lags. Missing Cq weeks were linearly interpolated only to fit and apply the prewhitening filter. Cq interp. reports that fraction. The white-noise reference is descriptive and no multiplicity correction was applied.

Table S2a. Lagged regression of each epidemiological metric on the weekly median Cq value at the calibrated lag, under four nested specifications. The table reports point estimates and primary moving-block bootstrap confidence intervals.

Series	Outcome	Model	Lag	N	Est.	Beta	Boot low	Boot high	R^2
Chih. w1	casos_dge	M1	5	8	Yes	−35.049	−71.8547	−21.7315	0.7314
Chih. w1	casos_dge	M2	5	8	No				
Chih. w1	casos_dge	M3	5	8	No				
Chih. w1	casos_dge	M4	5	8	No				
Chih. w1	casos_lab	M1	5	8	Yes	−26.9255	−73.2451	−14.2802	0.5991
Chih. w1	casos_lab	M2	5	8	No				
Chih. w1	casos_lab	M3	5	8	No				
Chih. w1	casos_lab	M4	5	8	No				
Chih. w1	hosp_n	M1	5	8	Yes	−0.0093	−6.0717	4.5137	0
Chih. w1	hosp_n	M2	5	8	No				
Chih. w1	hosp_n	M3	5	8	No				
Chih. w1	hosp_n	M4	5	8	No				
Chih. w1	hosp_prop	M1	1	12	Yes	−3.9639	−10.9209	−1.1898	0.3899
Chih. w1	hosp_prop	M2	1	12	No				
Chih. w1	hosp_prop	M3	1	12	No				
Chih. w1	hosp_prop	M4	1	12	No				
Chih. w1	hosp_tasa	M1	5	8	Yes	−0.0002	−0.1531	0.1138	0
Chih. w1	hosp_tasa	M2	5	8	No				
Chih. w1	hosp_tasa	M3	5	8	No				
Chih. w1	hosp_tasa	M4	5	8	No				
Chih. w1	neum_n	M1	5	8	Yes	−1.224	−5.4317	2.4226	0.0639
Chih. w1	neum_n	M2	5	8	No				
Chih. w1	neum_n	M3	5	8	No				
Chih. w1	neum_n	M4	5	8	No				
Chih. w1	neum_prop	M1	1	12	Yes	−1.6847	−4.006	0.0454	0.3338
Chih. w1	neum_prop	M2	1	12	No				
Chih. w1	neum_prop	M3	1	12	No				
Chih. w1	neum_prop	M4	1	12	No				

continues on the next page

Table S2a, continued

Series	Outcome	Model	Lag	N	Est.	Beta	Boot low	Boot high	R^2
Chih. w1	neum_tasa	M1	5	8	Yes	−0.0309	−0.137	0.0611	0.0639
Chih. w1	neum_tasa	M2	5	8	No				
Chih. w1	neum_tasa	M3	5	8	No				
Chih. w1	neum_tasa	M4	5	8	No				
Chih. w1	rt_dge	M1	2	11	Yes	−0.5968	−0.6108	−0.1868	0.6352
Chih. w1	rt_dge	M2	2	11	No				
Chih. w1	rt_dge	M3	2	11	No				
Chih. w1	rt_dge	M4	2	11	No				
Chih. w1	rt_lab	M1	2	11	Yes	−0.5247	−0.5508	−0.0897	0.4948
Chih. w1	rt_lab	M2	2	11	No				
Chih. w1	rt_lab	M3	2	11	No				
Chih. w1	rt_lab	M4	2	11	No				
Chih. w1	tasa_dge	M1	5	8	Yes	−0.8839	−1.8121	−0.548	0.7314
Chih. w1	tasa_dge	M2	5	8	No				
Chih. w1	tasa_dge	M3	5	8	No				
Chih. w1	tasa_dge	M4	5	8	No				
Chih. w1	tasa_lab	M1	5	8	Yes	−0.679	−1.8472	−0.3601	0.5991
Chih. w1	tasa_lab	M2	5	8	No				
Chih. w1	tasa_lab	M3	5	8	No				
Chih. w1	tasa_lab	M4	5	8	No				
Chih. w2	casos_dge	M1	9	18	Yes	−17.3468	−42.0852	2.6345	0.1856
Chih. w2	casos_dge	M2	9	18	Yes	−9.5201	−28.5951	4.5786	0.6409
Chih. w2	casos_dge	M3	9	18	No				
Chih. w2	casos_dge	M4	9	18	No				
Chih. w2	casos_lab	M1	9	18	Yes	−15.1266	−35.6613	2.4479	0.1764
Chih. w2	casos_lab	M2	9	18	Yes	−8.0516	−24.2579	4.3558	0.6282
Chih. w2	casos_lab	M3	9	18	No				
Chih. w2	casos_lab	M4	9	18	No				
Chih. w2	hosp_n	M1	9	18	Yes	−3.7177	−10.7576	1.376	0.1152
Chih. w2	hosp_n	M2	9	18	Yes	−1.5787	−7.5856	1.7962	0.5424
Chih. w2	hosp_n	M3	9	18	No				
Chih. w2	hosp_n	M4	9	18	No				
Chih. w2	hosp_prop	M1	1	25	Yes	−1.9452	−6.0785	3.1996	0.0682

continues on the next page

Table S2a, continued

Series	Outcome	Model	Lag	N	Est.	Beta	Boot low	Boot high	R^2
Chih. w2	hosp_prop	M2	1	25	Yes	−2.412	−7.492	3.9514	0.1665
Chih. w2	hosp_prop	M3	1	25	Yes	−2.6922	−6.8971	3.7167	0.2308
Chih. w2	hosp_prop	M4	1	25	No				
Chih. w2	hosp_tasa	M1	9	18	Yes	−0.0938	−0.2735	0.034	0.1152
Chih. w2	hosp_tasa	M2	9	18	Yes	−0.0398	−0.1869	0.046	0.5424
Chih. w2	hosp_tasa	M3	9	18	No				
Chih. w2	hosp_tasa	M4	9	18	No				
Chih. w2	neum_n	M1	9	18	Yes	−2.8332	−8.4527	1.4665	0.0915
Chih. w2	neum_n	M2	9	18	Yes	−0.9092	−5.6774	1.8219	0.5461
Chih. w2	neum_n	M3	9	18	No				
Chih. w2	neum_n	M4	9	18	No				
Chih. w2	neum_prop	M1	1	25	Yes	−2.7659	−3.5643	2.0393	0.1923
Chih. w2	neum_prop	M2	1	25	Yes	−2.611	−6.1381	2.3915	0.3426
Chih. w2	neum_prop	M3	1	25	Yes	−2.5621	−6.2461	2.761	0.3453
Chih. w2	neum_prop	M4	1	25	No				
Chih. w2	neum_tasa	M1	9	18	Yes	−0.0715	−0.2185	0.0369	0.0915
Chih. w2	neum_tasa	M2	9	18	Yes	−0.0229	−0.1433	0.0382	0.5461
Chih. w2	neum_tasa	M3	9	18	No				
Chih. w2	neum_tasa	M4	9	18	No				
Chih. w2	rt_dge	M1	8	19	Yes	−0.1516	−0.239	−0.0617	0.5544
Chih. w2	rt_dge	M2	8	19	Yes	−0.1563	−0.2514	−0.0575	0.5949
Chih. w2	rt_dge	M3	8	19	No				
Chih. w2	rt_dge	M4	8	19	No				
Chih. w2	rt_lab	M1	8	19	Yes	−0.1406	−0.2624	−0.0757	0.5543
Chih. w2	rt_lab	M2	8	19	Yes	−0.1447	−0.2864	−0.0735	0.5778
Chih. w2	rt_lab	M3	8	19	No				
Chih. w2	rt_lab	M4	8	19	No				
Chih. w2	tasa_dge	M1	9	18	Yes	−0.4375	−1.0686	0.0684	0.1856
Chih. w2	tasa_dge	M2	9	18	Yes	−0.2401	−0.7085	0.1272	0.6409
Chih. w2	tasa_dge	M3	9	18	No				
Chih. w2	tasa_dge	M4	9	18	No				
Chih. w2	tasa_lab	M1	9	18	Yes	−0.3815	−0.9319	0.0925	0.1764
Chih. w2	tasa_lab	M2	9	18	Yes	−0.2031	−0.6206	0.1292	0.6282

continues on the next page

Table S2a, continued

Series	Outcome	Model	Lag	N	Est.	Beta	Boot low	Boot high	R^2
Chih. w2	tasa_lab	M3	9	18	No				
Chih. w2	tasa_lab	M4	9	18	No				
Jalisco	casos_dge	M1	9	29	Yes	−121.5724	−183.5991	−34.5223	0.3295
Jalisco	casos_dge	M2	9	29	Yes	−96.9576	−123.1014	−8.9813	0.6424
Jalisco	casos_dge	M3	9	29	Yes	−45.0929	−81.487	18.827	0.7977
Jalisco	casos_dge	M4	9	29	No				
Jalisco	casos_lab	M1	9	29	Yes	−118.9839	−175.0842	−34.9455	0.3301
Jalisco	casos_lab	M2	9	29	Yes	−94.9523	−119.3094	−5.4016	0.6423
Jalisco	casos_lab	M3	9	29	Yes	−44.734	−76.4651	19.8261	0.7945
Jalisco	casos_lab	M4	9	29	No				
Jalisco	hosp_n	M1	9	29	Yes	−8.8855	−12.3819	−2.8707	0.2732
Jalisco	hosp_n	M2	9	29	Yes	−6.9962	−9.681	1.1686	0.5589
Jalisco	hosp_n	M3	9	29	Yes	−0.9591	−4.2896	2.0564	0.8854
Jalisco	hosp_n	M4	9	29	No				
Jalisco	hosp_prop	M1	3	35	Yes	−4.2576	−6.9538	−0.3963	0.22
Jalisco	hosp_prop	M2	3	35	Yes	−3.843	−7.3561	−0.3057	0.2338
Jalisco	hosp_prop	M3	3	35	Yes	−3.9656	−6.3587	0.447	0.3312
Jalisco	hosp_prop	M4	3	35	Yes	−4.1617	−5.9263	0.2205	0.4185
Jalisco	hosp_tasa	M1	9	29	Yes	−0.0989	−0.1328	−0.03	0.2732
Jalisco	hosp_tasa	M2	9	29	Yes	−0.0779	−0.1062	0.0135	0.5589
Jalisco	hosp_tasa	M3	9	29	Yes	−0.0107	−0.0463	0.0231	0.8854
Jalisco	hosp_tasa	M4	9	29	No				
Jalisco	neum_n	M1	9	29	Yes	−3.5308	−4.6457	−0.4126	0.2089
Jalisco	neum_n	M2	9	29	Yes	−2.6494	−3.9825	1.291	0.4786
Jalisco	neum_n	M3	9	29	Yes	0.4089	−0.7635	1.5597	0.8843
Jalisco	neum_n	M4	9	29	No				
Jalisco	neum_prop	M1	1	37	Yes	−1.7444	−4.6072	0.6382	0.1148
Jalisco	neum_prop	M2	1	37	Yes	−1.6085	−4.596	0.656	0.1484
Jalisco	neum_prop	M3	1	37	Yes	−1.9415	−4.4971	0.706	0.2822
Jalisco	neum_prop	M4	1	37	Yes	−1.9507	−4.4474	1.1488	0.2991
Jalisco	neum_tasa	M1	9	29	Yes	−0.0393	−0.0516	−0.0051	0.2089
Jalisco	neum_tasa	M2	9	29	Yes	−0.0295	−0.0454	0.0144	0.4786
Jalisco	neum_tasa	M3	9	29	Yes	0.0046	−0.0087	0.017	0.8843

continues on the next page

Table S2a, continued

Series	Outcome	Model	Lag	N	Est.	Beta	Boot low	Boot high	R^2
Jalisco	neum_tasa	M4	9	29	No				
Jalisco	rt_dge	M1	7	31	Yes	−0.1813	−0.3062	−0.0603	0.2151
Jalisco	rt_dge	M2	7	31	Yes	−0.1482	−0.2905	−0.0541	0.3934
Jalisco	rt_dge	M3	7	31	Yes	−0.0914	−0.2308	−0.0112	0.6351
Jalisco	rt_dge	M4	7	31	Yes	−0.117	−0.2346	−0.0249	0.7718
Jalisco	rt_lab	M1	7	31	Yes	−0.1994	−0.3622	−0.0576	0.2114
Jalisco	rt_lab	M2	7	31	Yes	−0.1661	−0.3454	−0.0534	0.3677
Jalisco	rt_lab	M3	7	31	Yes	−0.1069	−0.2583	−0.0092	0.5819
Jalisco	rt_lab	M4	7	31	Yes	−0.135	−0.2828	−0.021	0.7159
Jalisco	tasa_dge	M1	9	29	Yes	−1.3536	−2.109	−0.3531	0.3295
Jalisco	tasa_dge	M2	9	29	Yes	−1.0795	−1.4026	−0.0637	0.6424
Jalisco	tasa_dge	M3	9	29	Yes	−0.5021	−0.9094	0.2076	0.7977
Jalisco	tasa_dge	M4	9	29	No				
Jalisco	tasa_lab	M1	9	29	Yes	−1.3247	−2.0342	−0.3651	0.3301
Jalisco	tasa_lab	M2	9	29	Yes	−1.0572	−1.3651	−0.0886	0.6423
Jalisco	tasa_lab	M3	9	29	Yes	−0.4981	−0.8833	0.1788	0.7945
Jalisco	tasa_lab	M4	9	29	No				

Series: Chih. w1, Chihuahua wave 1 (February–May 2025); Chih. w2, Chihuahua wave 2 (May–November 2025); Jalisco, Jalisco single wave (November 2025–July 2026). Outcome codes are defined in Table S0b. Models: M1, unadjusted; M2, adjusted for median age and the proportion of women; M3, M2 plus a linear time trend; M4, M3 plus the Indigenous proportion and the number of Cq determinations of the week. Coefficients are expressed in units of the outcome per additional Cq cycle. A negative coefficient indicates that a lower Cq value, corresponding to a higher viral RNA burden, precedes a higher value of the metric. The primary confidence intervals use moving blocks of four weeks and 2,000 replicates with seed 20260803. Specifications that do not reach five observations per predictor, excluding the intercept, are marked as not estimable and left blank.

Table S2b. Lagged regression, continued from Table S2a: classical and Newey–West HAC sensitivity inference for the same models.

Series	Outcome	Model	Lag	N	Est.	SE	p	CI low	CI high	HAC SE	HAC p	HAC low	HAC high
Chih. w1	casos_dge	M1	5	8	Yes	8.6719	0.007	−56.2683	−13.8296	7.8588	0.004	−54.2787	−15.8192
Chih. w1	casos_dge	M2	5	8	No								
Chih. w1	casos_dge	M3	5	8	No								
Chih. w1	casos_dge	M4	5	8	No								
Chih. w1	casos_lab	M1	5	8	Yes	8.9913	0.024	−48.9265	−4.9244	8.2985	0.018	−47.2311	−6.6198
Chih. w1	casos_lab	M2	5	8	No								
Chih. w1	casos_lab	M3	5	8	No								
Chih. w1	casos_lab	M4	5	8	No								
Chih. w1	hosp_n	M1	5	8	Yes	2.2483	0.997	−5.5106	5.4921	2.1274	0.997	−5.2148	5.1963
Chih. w1	hosp_n	M2	5	8	No								
Chih. w1	hosp_n	M3	5	8	No								
Chih. w1	hosp_n	M4	5	8	No								
Chih. w1	hosp_prop	M1	1	12	Yes	1.5679	0.03	−7.4573	−0.4705	1.313	0.013	−6.8894	−1.0385
Chih. w1	hosp_prop	M2	1	12	No								
Chih. w1	hosp_prop	M3	1	12	No								
Chih. w1	hosp_prop	M4	1	12	No								
Chih. w1	hosp_tasa	M1	5	8	Yes	0.0567	0.997	−0.139	0.1385	0.0537	0.997	−0.1315	0.131
Chih. w1	hosp_tasa	M2	5	8	No								
Chih. w1	hosp_tasa	M3	5	8	No								
Chih. w1	hosp_tasa	M4	5	8	No								
Chih. w1	neum_n	M1	5	8	Yes	1.9129	0.546	−5.9048	3.4567	1.9847	0.56	−6.0805	3.6325
Chih. w1	neum_n	M2	5	8	No								
Chih. w1	neum_n	M3	5	8	No								
Chih. w1	neum_n	M4	5	8	No								
Chih. w1	neum_prop	M1	1	12	Yes	0.7526	0.049	−3.3616	−0.0078	0.6014	0.019	−3.0247	−0.3447
Chih. w1	neum_prop	M2	1	12	No								
Chih. w1	neum_prop	M3	1	12	No								
Chih. w1	neum_prop	M4	1	12	No								
Chih. w1	neum_tasa	M1	5	8	Yes	0.0482	0.546	−0.1489	0.0872	0.0501	0.56	−0.1533	0.0916
Chih. w1	neum_tasa	M2	5	8	No								

continues on the next page

Table S2b, continued

Series	Outcome	Model	Lag	N	Est.	SE	p	CI low	CI high	HAC SE	HAC p	HAC low	HAC high
Chih. w1	neum_tasa	M3	5	8	No								
Chih. w1	neum_tasa	M4	5	8	No								
Chih. w1	rt_dge	M1	2	11	Yes	0.1507	0.003	−0.9378	−0.2558	0.0854	<0.001	−0.7899	−0.4037
Chih. w1	rt_dge	M2	2	11	No								
Chih. w1	rt_dge	M3	2	11	No								
Chih. w1	rt_dge	M4	2	11	No								
Chih. w1	rt_lab	M1	2	11	Yes	0.1767	0.016	−0.9245	−0.1249	0.0696	<0.001	−0.6822	−0.3672
Chih. w1	rt_lab	M2	2	11	No								
Chih. w1	rt_lab	M3	2	11	No								
Chih. w1	rt_lab	M4	2	11	No								
Chih. w1	tasa_dge	M1	5	8	Yes	0.2187	0.007	−1.419	−0.3488	0.1982	0.004	−1.3689	−0.3989
Chih. w1	tasa_dge	M2	5	8	No								
Chih. w1	tasa_dge	M3	5	8	No								
Chih. w1	tasa_dge	M4	5	8	No								
Chih. w1	tasa_lab	M1	5	8	Yes	0.2268	0.024	−1.2339	−0.1242	0.2093	0.018	−1.1911	−0.1669
Chih. w1	tasa_lab	M2	5	8	No								
Chih. w1	tasa_lab	M3	5	8	No								
Chih. w1	tasa_lab	M4	5	8	No								
Chih. w2	casos_dge	M1	9	18	Yes	9.0846	0.074	−36.6053	1.9117	10.6683	0.123	−39.9625	5.2689
Chih. w2	casos_dge	M2	9	18	Yes	6.8214	0.185	−24.1506	5.1103	5.7231	0.118	−21.795	2.7547
Chih. w2	casos_dge	M3	9	18	No								
Chih. w2	casos_dge	M4	9	18	No								
Chih. w2	casos_lab	M1	9	18	Yes	8.1716	0.083	−32.4496	2.1964	9.2049	0.12	−34.6402	4.387
Chih. w2	casos_lab	M2	9	18	Yes	6.2089	0.216	−21.3684	5.2652	4.7955	0.115	−18.3369	2.2337
Chih. w2	casos_lab	M3	9	18	No								
Chih. w2	casos_lab	M4	9	18	No								
Chih. w2	hosp_n	M1	9	18	Yes	2.5752	0.168	−9.1769	1.7416	2.8142	0.205	−9.6835	2.2482
Chih. w2	hosp_n	M2	9	18	Yes	2.0945	0.463	−6.0709	2.9134	1.512	0.314	−4.8217	1.6642
Chih. w2	hosp_n	M3	9	18	No								
Chih. w2	hosp_n	M4	9	18	No								
Chih. w2	hosp_prop	M1	1	25	Yes	1.4998	0.207	−5.0477	1.1572	1.4186	0.184	−4.8797	0.9893
Chih. w2	hosp_prop	M2	1	25	Yes	1.6913	0.169	−5.9293	1.1053	1.4031	0.1	−5.3299	0.5059
Chih. w2	hosp_prop	M3	1	25	Yes	1.6789	0.124	−6.1943	0.81	1.3264	0.056	−5.459	0.0747

continues on the next page

Table S2b, continued

Series	Outcome	Model	Lag	N	Est.	SE	p	CI low	CI high	HAC SE	HAC p	HAC low	HAC high
Chih. w2	hosp_prop	M4	1	25	No								
Chih. w2	hosp_tasa	M1	9	18	Yes	0.0649	0.168	−0.2314	0.0439	0.071	0.205	−0.2442	0.0567
Chih. w2	hosp_tasa	M2	9	18	Yes	0.0528	0.463	−0.1531	0.0735	0.0381	0.314	−0.1216	0.042
Chih. w2	hosp_tasa	M3	9	18	No								
Chih. w2	hosp_tasa	M4	9	18	No								
Chih. w2	neum_n	M1	9	18	Yes	2.2325	0.223	−7.5659	1.8995	2.2715	0.23	−7.6486	1.9822
Chih. w2	neum_n	M2	9	18	Yes	1.7845	0.618	−4.7366	2.9181	1.0052	0.381	−3.0651	1.2467
Chih. w2	neum_n	M3	9	18	No								
Chih. w2	neum_n	M4	9	18	No								
Chih. w2	neum_prop	M1	1	25	Yes	1.1818	0.028	−5.2107	−0.3212	0.8551	0.004	−4.5349	−0.9969
Chih. w2	neum_prop	M2	1	25	Yes	1.2713	0.053	−5.2549	0.0329	1.1347	0.032	−4.9707	−0.2513
Chih. w2	neum_prop	M3	1	25	Yes	1.311	0.065	−5.2967	0.1726	1.346	0.071	−5.3698	0.2456
Chih. w2	neum_prop	M4	1	25	No								
Chih. w2	neum_tasa	M1	9	18	Yes	0.0563	0.223	−0.1908	0.0479	0.0573	0.23	−0.1929	0.05
Chih. w2	neum_tasa	M2	9	18	Yes	0.045	0.618	−0.1195	0.0736	0.0253	0.381	−0.0773	0.0314
Chih. w2	neum_tasa	M3	9	18	No								
Chih. w2	neum_tasa	M4	9	18	No								
Chih. w2	rt_dge	M1	8	19	Yes	0.033	<0.001	−0.2212	−0.0821	0.0348	<0.001	−0.225	−0.0783
Chih. w2	rt_dge	M2	8	19	Yes	0.0349	<0.001	−0.2306	−0.082	0.0401	0.001	−0.2418	−0.0707
Chih. w2	rt_dge	M3	8	19	No								
Chih. w2	rt_dge	M4	8	19	No								
Chih. w2	rt_lab	M1	8	19	Yes	0.0306	<0.001	−0.2051	−0.0761	0.0298	<0.001	−0.2034	−0.0778
Chih. w2	rt_lab	M2	8	19	Yes	0.033	<0.001	−0.215	−0.0744	0.0347	<0.001	−0.2187	−0.0706
Chih. w2	rt_lab	M3	8	19	No								
Chih. w2	rt_lab	M4	8	19	No								
Chih. w2	tasa_dge	M1	9	18	Yes	0.2291	0.074	−0.9231	0.0482	0.269	0.123	−1.0078	0.1329
Chih. w2	tasa_dge	M2	9	18	Yes	0.172	0.185	−0.6091	0.1289	0.1443	0.118	−0.5496	0.0695
Chih. w2	tasa_dge	M3	9	18	No								
Chih. w2	tasa_dge	M4	9	18	No								
Chih. w2	tasa_lab	M1	9	18	Yes	0.2061	0.083	−0.8183	0.0554	0.2321	0.12	−0.8736	0.1106
Chih. w2	tasa_lab	M2	9	18	Yes	0.1566	0.216	−0.5389	0.1328	0.1209	0.115	−0.4624	0.0563
Chih. w2	tasa_lab	M3	9	18	No								
Chih. w2	tasa_lab	M4	9	18	No								

continues on the next page

Table S2b, continued

Series	Outcome	Model	Lag	N	Est.	SE	p	CI low	CI high	HAC SE	HAC p	HAC low	HAC high
Jalisco	casos_dge	M1	9	29	Yes	33.3756	0.001	−190.0535	−53.0913	27.164	<0.001	−177.3084	−65.8364
Jalisco	casos_dge	M2	9	29	Yes	25.9878	<0.001	−150.4805	−43.4346	16.4178	<0.001	−130.7708	−63.1444
Jalisco	casos_dge	M3	9	29	Yes	23.3257	0.065	−93.2348	3.0489	23.6663	0.069	−93.9377	3.7518
Jalisco	casos_dge	M4	9	29	No								
Jalisco	casos_lab	M1	9	29	Yes	32.6234	0.001	−185.9216	−52.0461	27.1242	<0.001	−174.6382	−63.3295
Jalisco	casos_lab	M2	9	29	Yes	25.4173	<0.001	−147.3002	−42.6044	16.1483	<0.001	−128.2104	−61.6942
Jalisco	casos_lab	M3	9	29	Yes	22.9872	0.063	−92.1773	2.7093	23.0755	0.064	−92.3594	2.8915
Jalisco	casos_lab	M4	9	29	No								
Jalisco	hosp_n	M1	9	29	Yes	2.7892	0.004	−14.6086	−3.1625	1.2934	<0.001	−11.5395	−6.2316
Jalisco	hosp_n	M2	9	29	Yes	2.3168	0.006	−11.7678	−2.2246	1.8162	<0.001	−10.7367	−3.2556
Jalisco	hosp_n	M3	9	29	Yes	1.4091	0.503	−3.8674	1.9491	1.2016	0.433	−3.4391	1.5208
Jalisco	hosp_n	M4	9	29	No								
Jalisco	hosp_prop	M1	3	35	Yes	1.3954	0.004	−7.0965	−1.4188	2.3795	0.083	−9.0989	0.5836
Jalisco	hosp_prop	M2	3	35	Yes	1.5386	0.018	−6.9809	−0.7051	2.1398	0.082	−8.2072	0.5212
Jalisco	hosp_prop	M3	3	35	Yes	1.4623	0.011	−6.9521	−0.9792	1.6908	0.026	−7.4186	−0.5126
Jalisco	hosp_prop	M4	3	35	Yes	1.4152	0.006	−7.0605	−1.2628	1.5639	0.013	−7.3653	−0.9581
Jalisco	hosp_tasa	M1	9	29	Yes	0.0311	0.004	−0.1626	−0.0352	0.0144	<0.001	−0.1285	−0.0694
Jalisco	hosp_tasa	M2	9	29	Yes	0.0258	0.006	−0.131	−0.0248	0.0202	<0.001	−0.1195	−0.0362
Jalisco	hosp_tasa	M3	9	29	Yes	0.0157	0.503	−0.0431	0.0217	0.0134	0.433	−0.0383	0.0169
Jalisco	hosp_tasa	M4	9	29	No								
Jalisco	neum_n	M1	9	29	Yes	1.3224	0.013	−6.2441	−0.8174	0.6253	<0.001	−4.8137	−2.2478
Jalisco	neum_n	M2	9	29	Yes	1.1447	0.029	−5.007	−0.2917	0.9619	0.011	−4.6305	−0.6682
Jalisco	neum_n	M3	9	29	Yes	0.6435	0.531	−0.9192	1.7369	0.4795	0.402	−0.5809	1.3986
Jalisco	neum_n	M4	9	29	No								
Jalisco	neum_prop	M1	1	37	Yes	0.8187	0.04	−3.4064	−0.0824	1.4691	0.243	−4.7267	1.2379
Jalisco	neum_prop	M2	1	37	Yes	0.8656	0.072	−3.3697	0.1526	1.3915	0.256	−4.4396	1.2226
Jalisco	neum_prop	M3	1	37	Yes	0.8185	0.024	−3.6087	−0.2742	1.4139	0.179	−4.8215	0.9386
Jalisco	neum_prop	M4	1	37	Yes	0.8435	0.028	−3.6733	−0.2281	1.5523	0.219	−5.121	1.2196
Jalisco	neum_tasa	M1	9	29	Yes	0.0147	0.013	−0.0695	−0.0091	0.007	<0.001	−0.0536	−0.025
Jalisco	neum_tasa	M2	9	29	Yes	0.0127	0.029	−0.0557	−0.0032	0.0107	0.011	−0.0516	−0.0074
Jalisco	neum_tasa	M3	9	29	Yes	0.0072	0.531	−0.0102	0.0193	0.0053	0.402	−0.0065	0.0156
Jalisco	neum_tasa	M4	9	29	No								
Jalisco	rt_dge	M1	7	31	Yes	0.0643	0.009	−0.3129	−0.0498	0.0558	0.003	−0.2955	−0.0671

continues on the next page

Table S2b, continued

Series	Outcome	Model	Lag	N	Est.	SE	p	CI low	CI high	HAC SE	HAC p	HAC low	HAC high
Jalisco	rt_dge	M2	7	31	Yes	0.0604	0.021	−0.272	−0.0243	0.0427	0.002	−0.2357	−0.0606
Jalisco	rt_dge	M3	7	31	Yes	0.0496	0.077	−0.1935	0.0106	0.0339	0.012	−0.1612	−0.0217
Jalisco	rt_dge	M4	7	31	Yes	0.0415	0.009	−0.2026	−0.0315	0.0359	0.003	−0.1911	−0.043
Jalisco	rt_lab	M1	7	31	Yes	0.0715	0.009	−0.3457	−0.0532	0.0658	0.005	−0.3339	−0.0649
Jalisco	rt_lab	M2	7	31	Yes	0.0683	0.022	−0.3063	−0.0258	0.0533	0.004	−0.2755	−0.0567
Jalisco	rt_lab	M3	7	31	Yes	0.0589	0.081	−0.228	0.0142	0.0478	0.034	−0.2051	−0.0086
Jalisco	rt_lab	M4	7	31	Yes	0.0513	0.015	−0.2409	−0.0291	0.0487	0.011	−0.2354	−0.0346
Jalisco	tasa_dge	M1	9	29	Yes	0.3716	0.001	−2.116	−0.5911	0.3024	<0.001	−1.9741	−0.733
Jalisco	tasa_dge	M2	9	29	Yes	0.2893	<0.001	−1.6754	−0.4836	0.1828	<0.001	−1.456	−0.703
Jalisco	tasa_dge	M3	9	29	Yes	0.2597	0.065	−1.0381	0.0339	0.2635	0.069	−1.0459	0.0418
Jalisco	tasa_dge	M4	9	29	No								
Jalisco	tasa_lab	M1	9	29	Yes	0.3632	0.001	−2.07	−0.5795	0.302	<0.001	−1.9444	−0.7051
Jalisco	tasa_lab	M2	9	29	Yes	0.283	<0.001	−1.64	−0.4744	0.1798	<0.001	−1.4275	−0.6869
Jalisco	tasa_lab	M3	9	29	Yes	0.2559	0.063	−1.0263	0.0302	0.2569	0.064	−1.0283	0.0322
Jalisco	tasa_lab	M4	9	29	No								

Series: Chih. w1, Chihuahua wave 1 (February–May 2025); Chih. w2, Chihuahua wave 2 (May–November 2025); Jalisco, Jalisco single wave (November 2025–July 2026). Outcome codes are defined in Table S0b. Models: M1, unadjusted; M2, adjusted for median age and the proportion of women; M3, M2 plus a linear time trend; M4, M3 plus the Indigenous proportion and the number of Cq determinations of the week. Classical model-based inference and Newey–West HAC inference with lag 3 are reported as sensitivities to the moving-block bootstrap intervals in Table S2a. Rows appear in the same order as in Table S2a.

Table S3. Calibrated Cq lag for each epidemiological metric and analytic series, with the cross-correlation coefficient at that lag and the rule used to assign it.

Series	Outcome	Lag	r	p	N	WN ref.	Exceeds	Rule
Chih. w1	casos_dge	5	−0.8552	0.007	8	0.693	Yes	own
Chih. w1	casos_lab	5	−0.774	0.024	8	0.693	Yes	own
Chih. w1	hosp_n	5	−0.0017	0.997	8	0.693	No	inherited from incidence
Chih. w1	hosp_prop	1	−0.6245	0.03	12	0.5658	Yes	own
Chih. w1	hosp_tasa	5	−0.0017	0.997	8	0.693	No	inherited from incidence
Chih. w1	neum_n	5	−0.2527	0.546	8	0.693	No	inherited from incidence
Chih. w1	neum_prop	1	−0.5778	0.049	12	0.5658	Yes	own
Chih. w1	neum_tasa	5	−0.2527	0.546	8	0.693	No	inherited from incidence
Chih. w1	rt_dge	2	−0.797	0.003	11	0.591	Yes	own
Chih. w1	rt_lab	2	−0.7034	0.016	11	0.591	Yes	own
Chih. w1	tasa_dge	5	−0.8552	0.007	8	0.693	Yes	own
Chih. w1	tasa_lab	5	−0.774	0.024	8	0.693	Yes	own
Chih. w2	casos_dge	9	−0.4308	0.074	18	0.462	No	own
Chih. w2	casos_lab	9	−0.42	0.083	18	0.462	No	own
Chih. w2	hosp_n	9	−0.3395	0.168	18	0.462	No	inherited from incidence
Chih. w2	hosp_prop	1	−0.2611	0.207	25	0.392	No	own
Chih. w2	hosp_tasa	9	−0.3395	0.168	18	0.462	No	inherited from incidence
Chih. w2	neum_n	9	−0.3024	0.223	18	0.462	No	inherited from incidence
Chih. w2	neum_prop	1	−0.4386	0.028	25	0.392	Yes	own
Chih. w2	neum_tasa	9	−0.3024	0.223	18	0.462	No	inherited from incidence
Chih. w2	rt_dge	8	−0.7446	<0.001	19	0.4497	Yes	own
Chih. w2	rt_lab	8	−0.7445	<0.001	19	0.4497	Yes	own
Chih. w2	tasa_dge	9	−0.4308	0.074	18	0.462	No	own
Chih. w2	tasa_lab	9	−0.42	0.083	18	0.462	No	own
Jalisco	casos_dge	9	−0.574	0.001	29	0.364	Yes	own
Jalisco	casos_lab	9	−0.5745	0.001	29	0.364	Yes	own
Jalisco	hosp_n	9	−0.5227	0.004	29	0.364	Yes	inherited from incidence
Jalisco	hosp_prop	3	−0.4691	0.004	35	0.3313	Yes	own
Jalisco	hosp_tasa	9	−0.5227	0.004	29	0.364	Yes	inherited from incidence

continues on the next page

Table S3, continued

Series	Outcome	Lag	r	p	N	WN ref.	Exceeds	Rule
Jalisco	neum_n	9	−0.457	0.013	29	0.364	Yes	inherited from incidence
Jalisco	neum_prop	1	−0.3389	0.04	37	0.3222	Yes	own
Jalisco	neum_tasa	9	−0.457	0.013	29	0.364	Yes	inherited from incidence
Jalisco	rt_dge	7	−0.4637	0.009	31	0.352	Yes	own
Jalisco	rt_lab	7	−0.4598	0.009	31	0.352	Yes	own
Jalisco	tasa_dge	9	−0.574	0.001	29	0.364	Yes	own
Jalisco	tasa_lab	9	−0.5745	0.001	29	0.364	Yes	own

Series: Chih. w1, Chihuahua wave 1 (February–May 2025); Chih. w2, Chihuahua wave 2 (May–November 2025); Jalisco, Jalisco single wave (November 2025–July 2026). Outcome codes are defined in Table S0b. The calibrated lag is the positive lag with the most negative cross-correlation. Severity counts inherit the lag calibrated for incidence within the same series. This is because hospitalized and pneumonia cases are a subset of the confirmed cases indexed by the same rash-onset week.

Table S4a. Formal Granger causality by the Toda–Yamamoto procedure and vector autoregression, at the VAR lag order selected by the Akaike information criterion. The calibrated-lag specification is reported separately in Table S4b.

Series	Outcome	Weeks	Est.	p	$p{+}d$	TY χ^2	TY p	TY p HAC	Rev. p	Gr. p diff	β L1	p L1	R^2 adj
Chih. w1	casos_lab	12	not estimable										
Chih. w1	casos_dge	12	not estimable										
Chih. w1	tasa_lab	12	not estimable										
Chih. w1	tasa_dge	12	not estimable										
Chih. w1	rt_lab	12	not estimable										
Chih. w1	rt_dge	12	not estimable										
Chih. w1	hosp_n	12	not estimable										
Chih. w1	hosp_tasa	12	not estimable										
Chih. w1	hosp_prop	12	not estimable										
Chih. w1	neum_n	12	not estimable										
Chih. w1	neum_tasa	12	not estimable										
Chih. w1	neum_prop	12	not estimable										
Chih. w2	casos_lab	25	below threshold; descriptive	1	2	0.105	0.746	0.221	0.895	0.606	−0.9967	0.75	0.869
Chih. w2	casos_dge	25	below threshold; descriptive	1	2	0.123	0.725	0.216	0.912	0.487	−1.001	0.729	0.904
Chih. w2	tasa_lab	25	below threshold; descriptive	1	2	0.105	0.746	0.221	0.895	0.606	−0.02514	0.75	0.869
Chih. w2	tasa_dge	25	below threshold; descriptive	1	2	0.123	0.725	0.216	0.912	0.487	−0.02524	0.729	0.904
Chih. w2	rt_lab	25	below threshold; descriptive	1	2	0.384	0.536	0.457	0.345	0.836	−0.01681	0.543	0.062
Chih. w2	rt_dge	25	below threshold; descriptive	1	2	0.996	0.318	0.226	0.463	0.332	−0.02292	0.332	0.247
Chih. w2	hosp_n	25	below threshold; descriptive	1	2	0.18	0.671	0.317	0.944	0.531	−0.6031	0.676	0.719
Chih. w2	hosp_tasa	25	below threshold; descriptive	1	2	0.18	0.671	0.317	0.944	0.531	−0.01521	0.676	0.719
Chih. w2	hosp_prop	25	below threshold; descriptive	1	2	0.328	0.567	0.625	0.009	0.005	−1.203	0.574	0.142
Chih. w2	neum_n	25	below threshold; descriptive	1	2	0.084	0.772	0.446	0.999	0.592	−0.3449	0.776	0.703
Chih. w2	neum_tasa	25	below threshold; descriptive	1	2	0.084	0.772	0.446	0.999	0.592	−0.008697	0.776	0.703
Chih. w2	neum_prop	25	below threshold; descriptive	1	2	1.731	0.188	0.093	0.953	0.028	−1.79	0.205	0.159
Jalisco	casos_lab	38	estimable	1	2	0.14	0.708	0.219	0.91	0.824	−4.452	0.711	0.851
Jalisco	casos_dge	38	estimable	1	2	0.145	0.703	0.203	0.891	0.81	−4.547	0.706	0.855
Jalisco	tasa_lab	38	estimable	1	2	0.147	0.701	0.214	0.913	0.813	−0.0508	0.704	0.851
Jalisco	tasa_dge	38	estimable	1	2	0.153	0.696	0.198	0.894	0.8	−0.05188	0.699	0.855

continues on the next page

Table S4a, continued

Series	Outcome	Weeks	Est.	p	p+d	TY χ^2	TY p	TY p HAC	Rev. p	Gr. p diff	β L1	p L1	R^2 adj
Jalisco	rt_lab	38	estimable	1	2	2.868	0.09	0.044	0.589	0.307	−0.09733	0.1	0.426
Jalisco	rt_dge	38	estimable	1	2	2.319	0.128	0.067	0.545	0.408	−0.07739	0.138	0.466
Jalisco	hosp_n	38	estimable	1	2	0.001	0.98	0.973	0.881	0.431	0.03013	0.98	0.785
Jalisco	hosp_tasa	38	estimable	1	2	0.001	0.979	0.973	0.875	0.432	0.0003444	0.979	0.785
Jalisco	hosp_prop	38	estimable	2	3	0.386	0.824	0.66	0.945	0.069	0.07636	0.938	0.639
Jalisco	neum_n	38	estimable	1	2	0.132	0.716	0.658	0.886	0.603	−0.2194	0.719	0.739
Jalisco	neum_tasa	38	estimable	1	2	0.132	0.716	0.657	0.894	0.602	−0.002444	0.719	0.738
Jalisco	neum_prop	38	estimable	1	2	1.741	0.187	0.228	0.42	0.547	−0.9657	0.197	0.42

Series: Chih. w1, Chihuahua wave 1 (February–May 2025); Chih. w2, Chihuahua wave 2 (May–November 2025); Jalisco, Jalisco single wave (November 2025–July 2026). Outcome codes are defined in Table S0b. The Wald statistic is applied only to the first p lags of a VAR fitted in levels with $p + d_{\max}$ lags, with $d_{\max} = 1$. *Rev. p* tests the reverse direction, the outcome as the cause of the Cq value. *Gr. p diff* is the classical Granger test on first differences, reported as a sensitivity analysis. Rows marked *below threshold; descriptive* yielded 4.6 observations per predictor, below the prespecified minimum of five. The intercept was not counted as a predictor. These rows are not used for inference.

Table S4b. Calibrated-lag autoregressive association models.

Series	Outcome	Weeks	Cal. lag	n cal	Est. cal	β cal	p cal	p cal HAC	R^2 cal	β cal 2L	p cal 2L
Chih. w1	casos_lab	12	5		not estimable						
Chih. w1	casos_dge	12	5		not estimable						
Chih. w1	tasa_lab	12	5		not estimable						
Chih. w1	tasa_dge	12	5		not estimable						
Chih. w1	rt_lab	12	2		not estimable						
Chih. w1	rt_dge	12	2		not estimable						
Chih. w1	hosp_n	12	5		not estimable						
Chih. w1	hosp_tasa	12	5		not estimable						
Chih. w1	hosp_prop	12	1		not estimable						
Chih. w1	neum_n	12	5		not estimable						
Chih. w1	neum_tasa	12	5		not estimable						
Chih. w1	neum_prop	12	1		not estimable						
Chih. w2	casos_lab	25	9	16	estimable	−1.584	0.854	0.593	0.778	0.09098	0.991
Chih. w2	casos_dge	25	9	16	estimable	−2.86	0.729	0.397	0.835	−2.512	0.746
Chih. w2	tasa_lab	25	9	16	estimable	−0.03994	0.854	0.593	0.778	0.002294	0.991
Chih. w2	tasa_dge	25	9	16	estimable	−0.07211	0.729	0.397	0.835	−0.06334	0.746
Chih. w2	rt_lab	25	8	17	estimable	−0.1439	0.013	0.021	0.279	−0.1458	0.014
Chih. w2	rt_dge	25	8	17	estimable	−0.1189	0.028	0.059	0.257	−0.12	0.032
Chih. w2	hosp_n	25	9	16	estimable	−2.435	0.57	0.337	0.416	−1.725	0.654
Chih. w2	hosp_tasa	25	9	16	estimable	−0.0614	0.57	0.337	0.416	−0.04351	0.654
Chih. w2	hosp_prop	25	1	24	estimable	−2.211	0.197	0.183	−0.01	−2.343	0.315
Chih. w2	neum_n	25	9	16	estimable	−0.08266	0.981	0.978	0.479	−0.3355	0.921
Chih. w2	neum_tasa	25	9	16	estimable	−0.002084	0.981	0.978	0.479	−0.008461	0.921
Chih. w2	neum_prop	25	1	24	estimable	−2.501	0.064	0.007	0.081	−2.569	0.08
Jalisco	casos_lab	38	9	29	estimable	−26.71	0.137	0.145	0.856	−33.81	0.084
Jalisco	casos_dge	38	9	29	estimable	−27.04	0.134	0.132	0.861	−34.47	0.078
Jalisco	tasa_lab	38	9	29	estimable	−0.2975	0.137	0.145	0.856	−0.3765	0.084
Jalisco	tasa_dge	38	9	29	estimable	−0.3013	0.133	0.132	0.861	−0.3839	0.078
Jalisco	rt_lab	38	7	31	estimable	−0.128	0.031	0.048	0.531	−0.1197	0.047
Jalisco	rt_dge	38	7	31	estimable	−0.0986	0.052	0.027	0.583	−0.08561	0.092
Jalisco	hosp_n	38	9	29	estimable	−0.8545	0.611	0.24	0.81	−0.3128	0.857

continues on the next page

Table S4b, continued

Series	Outcome	Weeks	Cal. lag	n cal	Est. cal	β cal	p cal	p cal HAC	R^2 cal	β cal 2L	p cal 2L
Jalisco	hosp_tasa	38	9	29	estimable	−0.009466	0.611	0.242	0.812	−0.003336	0.863
Jalisco	hosp_prop	38	3	35	estimable	−4.411	0.003	0.054	0.202	−2.87	0.004
Jalisco	neum_n	38	9	29	estimable	−0.07787	0.929	0.875	0.743	−0.02353	0.979
Jalisco	neum_tasa	38	9	29	estimable	−0.0008479	0.93	0.877	0.745	−0.0002224	0.982
Jalisco	neum_prop	38	1	37	estimable	−1.554	0.072	0.222	0.092	−1.275	0.07

Series: Chih. w1, Chihuahua wave 1 (February–May 2025); Chih. w2, Chihuahua wave 2 (May–November 2025); Jalisco, Jalisco single wave (November 2025–July 2026). Outcome codes are defined in Table S0b. These models ask whether the Cq value observed at the data-calibrated lag adds information beyond the recent past of the outcome itself. They are autoregressive association models, not Toda–Yamamoto or other formal Granger-causality tests. Estimability was assessed separately from the VAR-order analysis. Every row with an estimate met the prespecified five-observations-per-parameter criterion. The last two columns repeat the model with two own lags of the outcome instead of one. Rows appear in the same order as in Table S4a.

Table S5. Augmented Dickey–Fuller and KPSS stationarity tests for the weekly median Cq value and each epidemiological metric, in levels and in first differences, by analytic series.

Series	Outcome	N	ADF lev	KPSS lev	Stat. lev	ADF diff	KPSS diff	Stat. diff
Chih. w1	casos_dge	12	0.522	0.1	No	0.902	0.074	No
Chih. w1	casos_lab	12	0.987	0.1	No	0.75	0.072	No
Chih. w1	ct_mediana	12	0.99	0.1	No	0.356	0.1	No
Chih. w1	hosp_n	12	0.01	0.1	Yes	0.234	0.1	No
Chih. w1	hosp_prop	12	0.903	0.1	No	0.364	0.047	No
Chih. w1	hosp_tasa	12	0.01	0.1	Yes	0.234	0.1	No
Chih. w1	neum_n	12	0.01	0.1	Yes	0.01	0.1	Yes
Chih. w1	neum_prop	12	0.927	0.091	No	0.427	0.056	No
Chih. w1	neum_tasa	12	0.01	0.1	Yes	0.01	0.1	Yes
Chih. w1	rt_dge	12	0.963	0.051	No	0.196	0.1	No
Chih. w1	rt_lab	12	0.727	0.067	No	0.172	0.1	No
Chih. w1	tasa_dge	12	0.522	0.1	No	0.902	0.074	No
Chih. w1	tasa_lab	12	0.987	0.1	No	0.75	0.072	No
Chih. w2	casos_dge	25	0.1	0.01	No	0.01	0.1	Yes
Chih. w2	casos_lab	25	0.132	0.01	No	0.01	0.1	Yes
Chih. w2	ct_mediana	25	0.979	0.1	No	0.649	0.1	No
Chih. w2	hosp_n	25	0.086	0.01	No	0.01	0.1	Yes
Chih. w2	hosp_prop	25	0.615	0.075	No	0.059	0.1	No
Chih. w2	hosp_tasa	25	0.086	0.01	No	0.01	0.1	Yes
Chih. w2	neum_n	25	0.034	0.01	No	0.01	0.1	Yes
Chih. w2	neum_prop	25	0.545	0.1	No	0.017	0.1	Yes
Chih. w2	neum_tasa	25	0.034	0.01	No	0.01	0.1	Yes
Chih. w2	rt_dge	25	0.18	0.05	No	0.015	0.1	Yes
Chih. w2	rt_lab	25	0.199	0.064	No	0.01	0.1	Yes
Chih. w2	tasa_dge	25	0.1	0.01	No	0.01	0.1	Yes
Chih. w2	tasa_lab	25	0.132	0.01	No	0.01	0.1	Yes
Jalisco	casos_dge	38	0.416	0.1	No	0.531	0.1	No
Jalisco	casos_lab	38	0.411	0.1	No	0.529	0.1	No
Jalisco	ct_mediana	38	0.247	0.1	No	0.021	0.1	Yes

continues on the next page

Table S5, continued

Series	Outcome	N	ADF lev	KPSS lev	Stat. lev	ADF diff	KPSS diff	Stat. diff
Jalisco	hosp_n	38	0.444	0.1	No	0.714	0.1	No
Jalisco	hosp_prop	38	0.99	0.1	No	0.665	0.092	No
Jalisco	hosp_tasa	38	0.442	0.1	No	0.714	0.1	No
Jalisco	neum_n	38	0.475	0.1	No	0.618	0.1	No
Jalisco	neum_prop	38	0.177	0.086	No	0.344	0.1	No
Jalisco	neum_tasa	38	0.472	0.1	No	0.622	0.1	No
Jalisco	rt_dge	38	0.471	0.01	No	0.022	0.1	Yes
Jalisco	rt_lab	38	0.349	0.01	No	0.01	0.1	Yes
Jalisco	tasa_dge	38	0.419	0.1	No	0.53	0.1	No
Jalisco	tasa_lab	38	0.413	0.1	No	0.528	0.1	No

Series: Chih. w1, Chihuahua wave 1 (February–May 2025); Chih. w2, Chihuahua wave 2 (May–November 2025); Jalisco, Jalisco single wave (November 2025–July 2026). Outcome codes are defined in Table S0b. A series is classified as stationary when the augmented Dickey–Fuller test rejects the unit root and the KPSS test does not reject stationarity. These results are the reason the Toda–Yamamoto procedure was used instead of a Granger test in levels.

Table S6a. Out-of-sample evaluation of incremental predictive gain from the aggregate Cq value: analytic configurations and fold counts. Each row is one configuration evaluated by rolling-origin one-step-ahead forecasting. Performance for the same rows is in Table S6b.

Unit	Outcome	Family	Code	Frame	Comp.	Lag sel.	k^*	Folds
Chihuahua wave 2	% hospitalized	Severity	hosp_prop	leadtime	B1	fixed	1	10
Chihuahua wave 2	% hospitalized	Severity	hosp_prop	leadtime	B1	internal	1	7
Chihuahua wave 2	% hospitalized	Severity	hosp_prop	nowcast	B1	fixed	1	10
Chihuahua wave 2	% hospitalized	Severity	hosp_prop	nowcast	B1	internal	1	7
Chihuahua wave 2	% with pneumonia	Severity	neum_prop	leadtime	B1	fixed	1	10
Chihuahua wave 2	% with pneumonia	Severity	neum_prop	nowcast	B1	fixed	1	10
Chihuahua, full period	DGE-confirmed case rate	Incidence	tasa_dge	leadtime	B1	fixed	9	15
Chihuahua, full period	DGE-confirmed case rate	Incidence	tasa_dge	leadtime	B1	internal	9	19
Chihuahua, full period	DGE-confirmed case rate	Incidence	tasa_dge	leadtime	B2	fixed	9	10
Chihuahua, full period	DGE-confirmed case rate	Incidence	tasa_dge	leadtime	B2	internal	9	14
Chihuahua, full period	DGE-confirmed case rate	Incidence	tasa_dge	leadtime	B3	internal	9	9
Chihuahua, full period	DGE-confirmed case rate	Incidence	tasa_dge	nowcast	B1	fixed	9	15
Chihuahua, full period	DGE-confirmed case rate	Incidence	tasa_dge	nowcast	B1	internal	9	19
Chihuahua, full period	DGE-confirmed case rate	Incidence	tasa_dge	nowcast	B2	fixed	9	10
Chihuahua, full period	DGE-confirmed case rate	Incidence	tasa_dge	nowcast	B2	internal	9	14
Chihuahua, full period	DGE-confirmed case rate	Incidence	tasa_dge	nowcast	B3	internal	9	9
Chihuahua, full period	DGE-confirmed cases	Incidence	casos_dge	leadtime	B1	fixed	9	15
Chihuahua, full period	DGE-confirmed cases	Incidence	casos_dge	leadtime	B1	internal	9	19
Chihuahua, full period	DGE-confirmed cases	Incidence	casos_dge	leadtime	B2	fixed	9	10
Chihuahua, full period	DGE-confirmed cases	Incidence	casos_dge	leadtime	B2	internal	9	14
Chihuahua, full period	DGE-confirmed cases	Incidence	casos_dge	leadtime	B3	internal	9	9
Chihuahua, full period	DGE-confirmed cases	Incidence	casos_dge	nowcast	B1	fixed	9	15
Chihuahua, full period	DGE-confirmed cases	Incidence	casos_dge	nowcast	B1	internal	9	19
Chihuahua, full period	DGE-confirmed cases	Incidence	casos_dge	nowcast	B2	fixed	9	10
Chihuahua, full period	DGE-confirmed cases	Incidence	casos_dge	nowcast	B2	internal	9	14
Chihuahua, full period	DGE-confirmed cases	Incidence	casos_dge	nowcast	B3	internal	9	9
Chihuahua, full period	RT-PCR case rate	Incidence	tasa_lab	leadtime	B1	fixed	9	15
Chihuahua, full period	RT-PCR case rate	Incidence	tasa_lab	leadtime	B1	internal	9	19

continues on the next page

Table S6a, continued

**Unit**	**Outcome**	**Family**	**Code**	**Frame**	**Comp.**	**Lag sel.**	k^*	**Folds**
Chihuahua, full period	RT-PCR case rate	Incidence	tasa_lab	leadtime	B2	fixed	9	10
Chihuahua, full period	RT-PCR case rate	Incidence	tasa_lab	leadtime	B2	internal	9	14
Chihuahua, full period	RT-PCR case rate	Incidence	tasa_lab	leadtime	B3	internal	9	9
Chihuahua, full period	RT-PCR case rate	Incidence	tasa_lab	nowcast	B1	fixed	9	15
Chihuahua, full period	RT-PCR case rate	Incidence	tasa_lab	nowcast	B1	internal	9	19
Chihuahua, full period	RT-PCR case rate	Incidence	tasa_lab	nowcast	B2	fixed	9	10
Chihuahua, full period	RT-PCR case rate	Incidence	tasa_lab	nowcast	B2	internal	9	14
Chihuahua, full period	RT-PCR case rate	Incidence	tasa_lab	nowcast	B3	internal	9	9
Chihuahua, full period	RT-PCR-positive cases	Incidence	casos_lab	leadtime	B1	fixed	9	15
Chihuahua, full period	RT-PCR-positive cases	Incidence	casos_lab	leadtime	B1	internal	9	19
Chihuahua, full period	RT-PCR-positive cases	Incidence	casos_lab	leadtime	B2	fixed	9	10
Chihuahua, full period	RT-PCR-positive cases	Incidence	casos_lab	leadtime	B2	internal	9	14
Chihuahua, full period	RT-PCR-positive cases	Incidence	casos_lab	leadtime	B3	internal	9	9
Chihuahua, full period	RT-PCR-positive cases	Incidence	casos_lab	nowcast	B1	fixed	9	15
Chihuahua, full period	RT-PCR-positive cases	Incidence	casos_lab	nowcast	B1	internal	9	19
Chihuahua, full period	RT-PCR-positive cases	Incidence	casos_lab	nowcast	B2	fixed	9	10
Chihuahua, full period	RT-PCR-positive cases	Incidence	casos_lab	nowcast	B2	internal	9	14
Chihuahua, full period	RT-PCR-positive cases	Incidence	casos_lab	nowcast	B3	internal	9	9
Chihuahua, full period	% hospitalized	Severity	hosp_prop	leadtime	B1	fixed	1	22
Chihuahua, full period	% hospitalized	Severity	hosp_prop	leadtime	B1	internal	1	23
Chihuahua, full period	% hospitalized	Severity	hosp_prop	leadtime	B2	fixed	1	17
Chihuahua, full period	% hospitalized	Severity	hosp_prop	leadtime	B2	internal	1	18
Chihuahua, full period	% hospitalized	Severity	hosp_prop	leadtime	B3	fixed	1	12
Chihuahua, full period	% hospitalized	Severity	hosp_prop	leadtime	B3	internal	1	13
Chihuahua, full period	% hospitalized	Severity	hosp_prop	nowcast	B1	fixed	1	22
Chihuahua, full period	% hospitalized	Severity	hosp_prop	nowcast	B1	internal	1	23
Chihuahua, full period	% hospitalized	Severity	hosp_prop	nowcast	B2	fixed	1	17
Chihuahua, full period	% hospitalized	Severity	hosp_prop	nowcast	B2	internal	1	18
Chihuahua, full period	% hospitalized	Severity	hosp_prop	nowcast	B3	fixed	1	12
Chihuahua, full period	% hospitalized	Severity	hosp_prop	nowcast	B3	internal	1	13
Chihuahua, full period	% with pneumonia	Severity	neum_prop	leadtime	B1	fixed	1	22
Chihuahua, full period	% with pneumonia	Severity	neum_prop	leadtime	B1	internal	1	22
Chihuahua, full period	% with pneumonia	Severity	neum_prop	leadtime	B2	fixed	1	17

continues on the next page

Table S6a, continued

Unit	Outcome	Family	Code	Frame	Comp.	Lag sel.	k^*	Folds
Chihuahua, full period	% with pneumonia	Severity	neum_prop	leadtime	B2	internal	1	16
Chihuahua, full period	% with pneumonia	Severity	neum_prop	leadtime	B3	fixed	1	12
Chihuahua, full period	% with pneumonia	Severity	neum_prop	leadtime	B3	internal	1	11
Chihuahua, full period	% with pneumonia	Severity	neum_prop	nowcast	B1	fixed	1	22
Chihuahua, full period	% with pneumonia	Severity	neum_prop	nowcast	B1	internal	1	22
Chihuahua, full period	% with pneumonia	Severity	neum_prop	nowcast	B2	fixed	1	17
Chihuahua, full period	% with pneumonia	Severity	neum_prop	nowcast	B2	internal	1	16
Chihuahua, full period	% with pneumonia	Severity	neum_prop	nowcast	B3	fixed	1	12
Chihuahua, full period	% with pneumonia	Severity	neum_prop	nowcast	B3	internal	1	11
Chihuahua, full period	Hospitalization rate	Severity	hosp_tasa	leadtime	B1	fixed	9	15
Chihuahua, full period	Hospitalization rate	Severity	hosp_tasa	leadtime	B1	internal	9	22
Chihuahua, full period	Hospitalization rate	Severity	hosp_tasa	leadtime	B2	fixed	9	10
Chihuahua, full period	Hospitalization rate	Severity	hosp_tasa	leadtime	B2	internal	9	16
Chihuahua, full period	Hospitalization rate	Severity	hosp_tasa	leadtime	B3	internal	9	11
Chihuahua, full period	Hospitalization rate	Severity	hosp_tasa	nowcast	B1	fixed	9	15
Chihuahua, full period	Hospitalization rate	Severity	hosp_tasa	nowcast	B1	internal	9	22
Chihuahua, full period	Hospitalization rate	Severity	hosp_tasa	nowcast	B2	fixed	9	10
Chihuahua, full period	Hospitalization rate	Severity	hosp_tasa	nowcast	B2	internal	9	16
Chihuahua, full period	Hospitalization rate	Severity	hosp_tasa	nowcast	B3	internal	9	11
Chihuahua, full period	Hospitalized cases	Severity	hosp_n	leadtime	B1	fixed	9	15
Chihuahua, full period	Hospitalized cases	Severity	hosp_n	leadtime	B1	internal	9	22
Chihuahua, full period	Hospitalized cases	Severity	hosp_n	leadtime	B2	fixed	9	10
Chihuahua, full period	Hospitalized cases	Severity	hosp_n	leadtime	B2	internal	9	16
Chihuahua, full period	Hospitalized cases	Severity	hosp_n	leadtime	B3	internal	9	11
Chihuahua, full period	Hospitalized cases	Severity	hosp_n	nowcast	B1	fixed	9	15
Chihuahua, full period	Hospitalized cases	Severity	hosp_n	nowcast	B1	internal	9	22
Chihuahua, full period	Hospitalized cases	Severity	hosp_n	nowcast	B2	fixed	9	10
Chihuahua, full period	Hospitalized cases	Severity	hosp_n	nowcast	B2	internal	9	16
Chihuahua, full period	Hospitalized cases	Severity	hosp_n	nowcast	B3	internal	9	11
Chihuahua, full period	Pneumonia cases	Severity	neum_n	leadtime	B1	fixed	9	15
Chihuahua, full period	Pneumonia cases	Severity	neum_n	leadtime	B1	internal	9	21
Chihuahua, full period	Pneumonia cases	Severity	neum_n	leadtime	B2	fixed	9	10
Chihuahua, full period	Pneumonia cases	Severity	neum_n	leadtime	B2	internal	9	16

continues on the next page

Table S6a, continued

Unit	Outcome	Family	Code	Frame	Comp.	Lag sel.	k^*	Folds
Chihuahua, full period	Pneumonia cases	Severity	neum_n	leadtime	B3	internal	9	11
Chihuahua, full period	Pneumonia cases	Severity	neum_n	nowcast	B1	fixed	9	15
Chihuahua, full period	Pneumonia cases	Severity	neum_n	nowcast	B1	internal	9	21
Chihuahua, full period	Pneumonia cases	Severity	neum_n	nowcast	B2	fixed	9	10
Chihuahua, full period	Pneumonia cases	Severity	neum_n	nowcast	B2	internal	9	16
Chihuahua, full period	Pneumonia cases	Severity	neum_n	nowcast	B3	internal	9	11
Chihuahua, full period	Pneumonia rate	Severity	neum_tasa	leadtime	B1	fixed	9	15
Chihuahua, full period	Pneumonia rate	Severity	neum_tasa	leadtime	B1	internal	9	21
Chihuahua, full period	Pneumonia rate	Severity	neum_tasa	leadtime	B2	fixed	9	10
Chihuahua, full period	Pneumonia rate	Severity	neum_tasa	leadtime	B2	internal	9	16
Chihuahua, full period	Pneumonia rate	Severity	neum_tasa	leadtime	B3	internal	9	11
Chihuahua, full period	Pneumonia rate	Severity	neum_tasa	nowcast	B1	fixed	9	15
Chihuahua, full period	Pneumonia rate	Severity	neum_tasa	nowcast	B1	internal	9	21
Chihuahua, full period	Pneumonia rate	Severity	neum_tasa	nowcast	B2	fixed	9	10
Chihuahua, full period	Pneumonia rate	Severity	neum_tasa	nowcast	B2	internal	9	16
Chihuahua, full period	Pneumonia rate	Severity	neum_tasa	nowcast	B3	internal	9	11
Chihuahua, full period	Rt (DGE cases)	Transmission	rt_dge	leadtime	B1	fixed	8	16
Chihuahua, full period	Rt (DGE cases)	Transmission	rt_dge	leadtime	B1	internal	8	22
Chihuahua, full period	Rt (DGE cases)	Transmission	rt_dge	leadtime	B2	fixed	8	11
Chihuahua, full period	Rt (DGE cases)	Transmission	rt_dge	leadtime	B2	internal	8	17
Chihuahua, full period	Rt (DGE cases)	Transmission	rt_dge	nowcast	B1	fixed	8	16
Chihuahua, full period	Rt (DGE cases)	Transmission	rt_dge	nowcast	B1	internal	8	22
Chihuahua, full period	Rt (DGE cases)	Transmission	rt_dge	nowcast	B2	fixed	8	11
Chihuahua, full period	Rt (DGE cases)	Transmission	rt_dge	nowcast	B2	internal	8	17
Chihuahua, full period	Rt (RT-PCR cases)	Transmission	rt_lab	leadtime	B1	fixed	8	16
Chihuahua, full period	Rt (RT-PCR cases)	Transmission	rt_lab	leadtime	B1	internal	8	22
Chihuahua, full period	Rt (RT-PCR cases)	Transmission	rt_lab	leadtime	B2	fixed	8	11
Chihuahua, full period	Rt (RT-PCR cases)	Transmission	rt_lab	leadtime	B2	internal	8	17
Chihuahua, full period	Rt (RT-PCR cases)	Transmission	rt_lab	nowcast	B1	fixed	8	16
Chihuahua, full period	Rt (RT-PCR cases)	Transmission	rt_lab	nowcast	B1	internal	8	22
Chihuahua, full period	Rt (RT-PCR cases)	Transmission	rt_lab	nowcast	B2	fixed	8	11
Chihuahua, full period	Rt (RT-PCR cases)	Transmission	rt_lab	nowcast	B2	internal	8	17
Jalisco wave	DGE-confirmed case rate	Incidence	tasa_dge	leadtime	B1	fixed	9	14

continues on the next page

Table S6a, continued

Unit	Outcome	Family	Code	Frame	Comp.	Lag sel.	k^*	Folds
Jalisco wave	DGE-confirmed case rate	Incidence	tasa_dge	leadtime	B1	internal	9	16
Jalisco wave	DGE-confirmed case rate	Incidence	tasa_dge	leadtime	B2	fixed	9	8
Jalisco wave	DGE-confirmed case rate	Incidence	tasa_dge	leadtime	B2	internal	9	10
Jalisco wave	DGE-confirmed case rate	Incidence	tasa_dge	nowcast	B1	fixed	9	14
Jalisco wave	DGE-confirmed case rate	Incidence	tasa_dge	nowcast	B1	internal	9	16
Jalisco wave	DGE-confirmed case rate	Incidence	tasa_dge	nowcast	B2	fixed	9	9
Jalisco wave	DGE-confirmed case rate	Incidence	tasa_dge	nowcast	B2	internal	9	10
Jalisco wave	DGE-confirmed case rate	Incidence	tasa_dge	nowcast	B3	internal	9	6
Jalisco wave	DGE-confirmed cases	Incidence	casos_dge	leadtime	B1	fixed	9	14
Jalisco wave	DGE-confirmed cases	Incidence	casos_dge	leadtime	B1	internal	9	16
Jalisco wave	DGE-confirmed cases	Incidence	casos_dge	leadtime	B2	fixed	9	8
Jalisco wave	DGE-confirmed cases	Incidence	casos_dge	leadtime	B2	internal	9	10
Jalisco wave	DGE-confirmed cases	Incidence	casos_dge	nowcast	B1	fixed	9	14
Jalisco wave	DGE-confirmed cases	Incidence	casos_dge	nowcast	B1	internal	9	16
Jalisco wave	DGE-confirmed cases	Incidence	casos_dge	nowcast	B2	fixed	9	9
Jalisco wave	DGE-confirmed cases	Incidence	casos_dge	nowcast	B2	internal	9	10
Jalisco wave	DGE-confirmed cases	Incidence	casos_dge	nowcast	B3	internal	9	6
Jalisco wave	RT-PCR case rate	Incidence	tasa_lab	leadtime	B1	fixed	9	14
Jalisco wave	RT-PCR case rate	Incidence	tasa_lab	leadtime	B1	internal	9	16
Jalisco wave	RT-PCR case rate	Incidence	tasa_lab	leadtime	B2	fixed	9	8
Jalisco wave	RT-PCR case rate	Incidence	tasa_lab	leadtime	B2	internal	9	10
Jalisco wave	RT-PCR case rate	Incidence	tasa_lab	nowcast	B1	fixed	9	14
Jalisco wave	RT-PCR case rate	Incidence	tasa_lab	nowcast	B1	internal	9	16
Jalisco wave	RT-PCR case rate	Incidence	tasa_lab	nowcast	B2	fixed	9	9
Jalisco wave	RT-PCR case rate	Incidence	tasa_lab	nowcast	B2	internal	9	10
Jalisco wave	RT-PCR case rate	Incidence	tasa_lab	nowcast	B3	internal	9	6
Jalisco wave	RT-PCR-positive cases	Incidence	casos_lab	leadtime	B1	fixed	9	14
Jalisco wave	RT-PCR-positive cases	Incidence	casos_lab	leadtime	B1	internal	9	16
Jalisco wave	RT-PCR-positive cases	Incidence	casos_lab	leadtime	B2	fixed	9	8
Jalisco wave	RT-PCR-positive cases	Incidence	casos_lab	leadtime	B2	internal	9	10
Jalisco wave	RT-PCR-positive cases	Incidence	casos_lab	nowcast	B1	fixed	9	14
Jalisco wave	RT-PCR-positive cases	Incidence	casos_lab	nowcast	B1	internal	9	16
Jalisco wave	RT-PCR-positive cases	Incidence	casos_lab	nowcast	B2	fixed	9	9

continues on the next page

Table S6a, continued

Unit	Outcome	Family	Code	Frame	Comp.	Lag sel.	k^*	Folds
Jalisco wave	RT-PCR-positive cases	Incidence	casos_lab	nowcast	B2	internal	9	10
Jalisco wave	RT-PCR-positive cases	Incidence	casos_lab	nowcast	B3	internal	9	6
Jalisco wave	% hospitalized	Severity	hosp_prop	leadtime	B1	fixed	3	20
Jalisco wave	% hospitalized	Severity	hosp_prop	leadtime	B1	internal	3	21
Jalisco wave	% hospitalized	Severity	hosp_prop	leadtime	B2	fixed	3	14
Jalisco wave	% hospitalized	Severity	hosp_prop	leadtime	B2	internal	3	15
Jalisco wave	% hospitalized	Severity	hosp_prop	leadtime	B3	fixed	3	9
Jalisco wave	% hospitalized	Severity	hosp_prop	leadtime	B3	internal	3	10
Jalisco wave	% hospitalized	Severity	hosp_prop	nowcast	B1	fixed	3	20
Jalisco wave	% hospitalized	Severity	hosp_prop	nowcast	B1	internal	3	21
Jalisco wave	% hospitalized	Severity	hosp_prop	nowcast	B2	fixed	3	15
Jalisco wave	% hospitalized	Severity	hosp_prop	nowcast	B2	internal	3	16
Jalisco wave	% hospitalized	Severity	hosp_prop	nowcast	B3	fixed	3	10
Jalisco wave	% hospitalized	Severity	hosp_prop	nowcast	B3	internal	3	11
Jalisco wave	% with pneumonia	Severity	neum_prop	leadtime	B1	fixed	1	22
Jalisco wave	% with pneumonia	Severity	neum_prop	leadtime	B1	internal	1	21
Jalisco wave	% with pneumonia	Severity	neum_prop	leadtime	B2	fixed	1	16
Jalisco wave	% with pneumonia	Severity	neum_prop	leadtime	B2	internal	1	15
Jalisco wave	% with pneumonia	Severity	neum_prop	leadtime	B3	fixed	1	11
Jalisco wave	% with pneumonia	Severity	neum_prop	leadtime	B3	internal	1	9
Jalisco wave	% with pneumonia	Severity	neum_prop	nowcast	B1	fixed	1	22
Jalisco wave	% with pneumonia	Severity	neum_prop	nowcast	B1	internal	1	21
Jalisco wave	% with pneumonia	Severity	neum_prop	nowcast	B2	fixed	1	16
Jalisco wave	% with pneumonia	Severity	neum_prop	nowcast	B2	internal	1	16
Jalisco wave	% with pneumonia	Severity	neum_prop	nowcast	B3	fixed	1	11
Jalisco wave	% with pneumonia	Severity	neum_prop	nowcast	B3	internal	1	11
Jalisco wave	Hospitalization rate	Severity	hosp_tasa	leadtime	B1	fixed	9	14
Jalisco wave	Hospitalization rate	Severity	hosp_tasa	leadtime	B1	internal	9	16
Jalisco wave	Hospitalization rate	Severity	hosp_tasa	leadtime	B2	fixed	9	8
Jalisco wave	Hospitalization rate	Severity	hosp_tasa	leadtime	B2	internal	9	10
Jalisco wave	Hospitalization rate	Severity	hosp_tasa	leadtime	B3	internal	9	7
Jalisco wave	Hospitalization rate	Severity	hosp_tasa	nowcast	B1	fixed	9	14
Jalisco wave	Hospitalization rate	Severity	hosp_tasa	nowcast	B1	internal	9	16

continues on the next page

Table S6a, continued

Unit	Outcome	Family	Code	Frame	Comp.	Lag sel.	k^*	Folds
Jalisco wave	Hospitalization rate	Severity	hosp_tasa	nowcast	B2	fixed	9	9
Jalisco wave	Hospitalization rate	Severity	hosp_tasa	nowcast	B2	internal	9	11
Jalisco wave	Hospitalization rate	Severity	hosp_tasa	nowcast	B3	internal	9	7
Jalisco wave	Hospitalized cases	Severity	hosp_n	leadtime	B1	fixed	9	14
Jalisco wave	Hospitalized cases	Severity	hosp_n	leadtime	B1	internal	9	16
Jalisco wave	Hospitalized cases	Severity	hosp_n	leadtime	B2	fixed	9	8
Jalisco wave	Hospitalized cases	Severity	hosp_n	leadtime	B2	internal	9	10
Jalisco wave	Hospitalized cases	Severity	hosp_n	leadtime	B3	internal	9	7
Jalisco wave	Hospitalized cases	Severity	hosp_n	nowcast	B1	fixed	9	14
Jalisco wave	Hospitalized cases	Severity	hosp_n	nowcast	B1	internal	9	16
Jalisco wave	Hospitalized cases	Severity	hosp_n	nowcast	B2	fixed	9	9
Jalisco wave	Hospitalized cases	Severity	hosp_n	nowcast	B2	internal	9	11
Jalisco wave	Hospitalized cases	Severity	hosp_n	nowcast	B3	internal	9	7
Jalisco wave	Pneumonia cases	Severity	neum_n	leadtime	B1	fixed	9	14
Jalisco wave	Pneumonia cases	Severity	neum_n	leadtime	B1	internal	9	16
Jalisco wave	Pneumonia cases	Severity	neum_n	leadtime	B2	fixed	9	8
Jalisco wave	Pneumonia cases	Severity	neum_n	leadtime	B2	internal	9	11
Jalisco wave	Pneumonia cases	Severity	neum_n	leadtime	B3	internal	9	7
Jalisco wave	Pneumonia cases	Severity	neum_n	nowcast	B1	fixed	9	14
Jalisco wave	Pneumonia cases	Severity	neum_n	nowcast	B1	internal	9	16
Jalisco wave	Pneumonia cases	Severity	neum_n	nowcast	B2	fixed	9	9
Jalisco wave	Pneumonia cases	Severity	neum_n	nowcast	B2	internal	9	11
Jalisco wave	Pneumonia cases	Severity	neum_n	nowcast	B3	internal	9	8
Jalisco wave	Pneumonia rate	Severity	neum_tasa	leadtime	B1	fixed	9	14
Jalisco wave	Pneumonia rate	Severity	neum_tasa	leadtime	B1	internal	9	18
Jalisco wave	Pneumonia rate	Severity	neum_tasa	leadtime	B2	fixed	9	8
Jalisco wave	Pneumonia rate	Severity	neum_tasa	leadtime	B2	internal	9	11
Jalisco wave	Pneumonia rate	Severity	neum_tasa	leadtime	B3	internal	9	7
Jalisco wave	Pneumonia rate	Severity	neum_tasa	nowcast	B1	fixed	9	14
Jalisco wave	Pneumonia rate	Severity	neum_tasa	nowcast	B1	internal	9	18
Jalisco wave	Pneumonia rate	Severity	neum_tasa	nowcast	B2	fixed	9	9
Jalisco wave	Pneumonia rate	Severity	neum_tasa	nowcast	B2	internal	9	11
Jalisco wave	Pneumonia rate	Severity	neum_tasa	nowcast	B3	internal	9	8

continues on the next page

Table S6a, continued

Unit	Outcome	Family	Code	Frame	Comp.	Lag sel.	k^*	Folds
Jalisco wave	Rt (DGE cases)	Transmission	rt_dge	leadtime	B1	fixed	7	16
Jalisco wave	Rt (DGE cases)	Transmission	rt_dge	leadtime	B1	internal	7	19
Jalisco wave	Rt (DGE cases)	Transmission	rt_dge	leadtime	B2	fixed	7	10
Jalisco wave	Rt (DGE cases)	Transmission	rt_dge	leadtime	B2	internal	7	13
Jalisco wave	Rt (DGE cases)	Transmission	rt_dge	nowcast	B1	fixed	7	16
Jalisco wave	Rt (DGE cases)	Transmission	rt_dge	nowcast	B1	internal	7	19
Jalisco wave	Rt (DGE cases)	Transmission	rt_dge	nowcast	B2	fixed	7	11
Jalisco wave	Rt (DGE cases)	Transmission	rt_dge	nowcast	B2	internal	7	13
Jalisco wave	Rt (RT-PCR cases)	Transmission	rt_lab	leadtime	B1	fixed	7	16
Jalisco wave	Rt (RT-PCR cases)	Transmission	rt_lab	leadtime	B1	internal	7	19
Jalisco wave	Rt (RT-PCR cases)	Transmission	rt_lab	leadtime	B2	fixed	7	10
Jalisco wave	Rt (RT-PCR cases)	Transmission	rt_lab	leadtime	B2	internal	7	13
Jalisco wave	Rt (RT-PCR cases)	Transmission	rt_lab	nowcast	B1	fixed	7	16
Jalisco wave	Rt (RT-PCR cases)	Transmission	rt_lab	nowcast	B1	internal	7	19
Jalisco wave	Rt (RT-PCR cases)	Transmission	rt_lab	nowcast	B2	fixed	7	11
Jalisco wave	Rt (RT-PCR cases)	Transmission	rt_lab	nowcast	B2	internal	7	13

Two framings were evaluated. In *nowcast*, the comparator has the outcome of the previous week. In *leadtime*, every predictor, including the outcome's own past, is lagged by k. Comparator and candidate are therefore equally blind. Comparators: B1, the outcome's own past; B2, B1 plus the weekly growth rate; B3, B2 plus the reproduction number. Two lag-selection modes were used. The *fixed* mode reuses the calibrated lag of the main analysis. This lag was selected in sample and therefore leaks information into the evaluation. The *internal* mode re-selects the lag within the training segment of every fold and is the leak-free variant. Configurations yielding fewer than six folds are not reported. The complete grid is given, not a selected subset.

Table S6b. Out-of-sample evaluation, continued from Table S6a: predictive performance for the same analytic configurations. Skill is $1 - \mathrm{RMSE}_{+\mathrm{Cq}}/\mathrm{RMSE}_{\mathrm{base}}$. A positive value means that Cq improved the forecast.

Unit	Outcome	Frame	Comp.	Lag sel.	RMSE base	RMSE +Cq	Skill	Win frac.	p	Lag used
Chihuahua wave 2	% hospitalized	leadtime	B1	fixed	22.261	24.283	−0.091	0.5	1	1
Chihuahua wave 2	% hospitalized	leadtime	B1	internal	29.241	34.956	−0.195	0.429	0.219	3
Chihuahua wave 2	% hospitalized	nowcast	B1	fixed	22.261	24.283	−0.091	0.5	1	1
Chihuahua wave 2	% hospitalized	nowcast	B1	internal	27.482	36.133	−0.315	0.429	0.688	3
Chihuahua wave 2	% with pneumonia	leadtime	B1	fixed	17.275	17.92	−0.037	0.6	0.846	1
Chihuahua wave 2	% with pneumonia	nowcast	B1	fixed	17.275	17.92	−0.037	0.6	0.846	1
Chihuahua, full period	DGE-confirmed case rate	leadtime	B1	fixed	3.142	2.176	0.307	0.867	<0.001	9
Chihuahua, full period	DGE-confirmed case rate	leadtime	B1	internal	2.664	2.726	−0.023	0.421	0.829	5
Chihuahua, full period	DGE-confirmed case rate	leadtime	B2	fixed	3.065	2.291	0.253	0.8	0.01	9
Chihuahua, full period	DGE-confirmed case rate	leadtime	B2	internal	2.307	2.348	−0.018	0.429	0.426	5
Chihuahua, full period	DGE-confirmed case rate	leadtime	B3	internal	1.804	1.509	0.164	0.556	0.82	5
Chihuahua, full period	DGE-confirmed case rate	nowcast	B1	fixed	0.493	0.635	−0.29	0.4	0.359	9
Chihuahua, full period	DGE-confirmed case rate	nowcast	B1	internal	0.593	0.945	−0.594	0.263	0.004	5
Chihuahua, full period	DGE-confirmed case rate	nowcast	B2	fixed	0.441	0.611	−0.386	0.5	0.77	9
Chihuahua, full period	DGE-confirmed case rate	nowcast	B2	internal	0.491	0.693	−0.41	0.357	0.296	5
Chihuahua, full period	DGE-confirmed case rate	nowcast	B3	internal	0.381	0.542	−0.423	0.333	0.301	5
Chihuahua, full period	DGE-confirmed cases	leadtime	B1	fixed	124.577	86.275	0.307	0.867	<0.001	9
Chihuahua, full period	DGE-confirmed cases	leadtime	B1	internal	105.624	108.092	−0.023	0.421	0.829	5
Chihuahua, full period	DGE-confirmed cases	leadtime	B2	fixed	85.726	87.898	−0.025	0.7	0.492	9
Chihuahua, full period	DGE-confirmed cases	leadtime	B2	internal	81.641	81.982	−0.004	0.643	0.426	5
Chihuahua, full period	DGE-confirmed cases	leadtime	B3	internal	65.684	61.924	0.057	0.444	0.652	5
Chihuahua, full period	DGE-confirmed cases	nowcast	B1	fixed	19.529	25.185	−0.29	0.4	0.359	9
Chihuahua, full period	DGE-confirmed cases	nowcast	B1	internal	23.524	37.488	−0.594	0.263	0.004	5
Chihuahua, full period	DGE-confirmed cases	nowcast	B2	fixed	15.032	24.82	−0.651	0.3	0.193	9
Chihuahua, full period	DGE-confirmed cases	nowcast	B2	internal	17.313	26.955	−0.557	0.286	0.035	5
Chihuahua, full period	DGE-confirmed cases	nowcast	B3	internal	9.749	24.273	−1.49	0.111	0.02	5
Chihuahua, full period	RT-PCR case rate	leadtime	B1	fixed	2.717	2.093	0.23	0.867	0.002	9
Chihuahua, full period	RT-PCR case rate	leadtime	B1	internal	2.254	2.456	−0.09	0.368	0.145	5
Chihuahua, full period	RT-PCR case rate	leadtime	B2	fixed	2.787	2.43	0.128	0.8	0.064	9

continues on the next page

Table S6b, continued

Unit	Outcome	Frame	Comp.	Lag sel.	RMSE base	RMSE +Cq	Skill	Win frac.	*p*	Lag used
Chihuahua, full period	RT-PCR case rate	leadtime	B2	internal	2.104	2.42	−0.15	0.286	0.013	5
Chihuahua, full period	RT-PCR case rate	leadtime	B3	internal	1.474	1.198	0.187	0.667	0.359	5
Chihuahua, full period	RT-PCR case rate	nowcast	B1	fixed	0.46	0.607	−0.32	0.467	0.421	9
Chihuahua, full period	RT-PCR case rate	nowcast	B1	internal	0.575	0.985	−0.713	0.105	<0.001	5
Chihuahua, full period	RT-PCR case rate	nowcast	B2	fixed	0.412	0.573	−0.39	0.4	0.625	9
Chihuahua, full period	RT-PCR case rate	nowcast	B2	internal	0.469	0.74	−0.576	0.214	0.013	5
Chihuahua, full period	RT-PCR case rate	nowcast	B3	internal	0.277	0.489	−0.766	0.222	0.027	5
Chihuahua, full period	RT-PCR-positive cases	leadtime	B1	fixed	107.732	82.997	0.23	0.867	0.002	9
Chihuahua, full period	RT-PCR-positive cases	leadtime	B1	internal	89.37	97.401	−0.09	0.368	0.145	5
Chihuahua, full period	RT-PCR-positive cases	leadtime	B2	fixed	90.741	93.947	−0.035	0.7	0.557	9
Chihuahua, full period	RT-PCR-positive cases	leadtime	B2	internal	78.538	82.941	−0.056	0.357	0.326	5
Chihuahua, full period	RT-PCR-positive cases	leadtime	B3	internal	56.822	47.508	0.164	0.667	0.055	5
Chihuahua, full period	RT-PCR-positive cases	nowcast	B1	fixed	18.242	24.081	−0.32	0.467	0.421	9
Chihuahua, full period	RT-PCR-positive cases	nowcast	B1	internal	22.798	39.062	−0.713	0.105	<0.001	5
Chihuahua, full period	RT-PCR-positive cases	nowcast	B2	fixed	15.877	23.171	−0.459	0.2	0.131	9
Chihuahua, full period	RT-PCR-positive cases	nowcast	B2	internal	19.01	28.532	−0.501	0.214	0.017	5
Chihuahua, full period	RT-PCR-positive cases	nowcast	B3	internal	10.336	22.106	−1.139	0.111	0.008	5
Chihuahua, full period	% hospitalized	leadtime	B1	fixed	15.512	17.658	−0.138	0.273	0.019	1
Chihuahua, full period	% hospitalized	leadtime	B1	internal	19.642	22.77	−0.159	0.261	0.004	1
Chihuahua, full period	% hospitalized	leadtime	B2	fixed	18.667	19.456	−0.042	0.235	0.008	1
Chihuahua, full period	% hospitalized	leadtime	B2	internal	22.942	25.325	−0.104	0.222	0.021	1
Chihuahua, full period	% hospitalized	leadtime	B3	fixed	21.469	22.237	−0.036	0.25	0.064	1
Chihuahua, full period	% hospitalized	leadtime	B3	internal	26.616	30.884	−0.16	0.308	0.146	1
Chihuahua, full period	% hospitalized	nowcast	B1	fixed	15.512	17.658	−0.138	0.273	0.019	1
Chihuahua, full period	% hospitalized	nowcast	B1	internal	18.929	23.426	−0.238	0.261	0.012	1
Chihuahua, full period	% hospitalized	nowcast	B2	fixed	18.667	19.456	−0.042	0.235	0.008	1
Chihuahua, full period	% hospitalized	nowcast	B2	internal	22.226	26.478	−0.191	0.222	0.048	1
Chihuahua, full period	% hospitalized	nowcast	B3	fixed	21.469	22.237	−0.036	0.25	0.064	1
Chihuahua, full period	% hospitalized	nowcast	B3	internal	25.179	32.6	−0.295	0.308	0.191	1
Chihuahua, full period	% with pneumonia	leadtime	B1	fixed	12.247	13.092	−0.069	0.364	0.849	1
Chihuahua, full period	% with pneumonia	leadtime	B1	internal	13.041	14.557	−0.116	0.409	0.337	1
Chihuahua, full period	% with pneumonia	leadtime	B2	fixed	13.975	14.374	−0.029	0.471	0.818	1
Chihuahua, full period	% with pneumonia	leadtime	B2	internal	14.908	15.818	−0.061	0.312	0.252	1

continues on the next page

Table S6b, continued

Unit	Outcome	Frame	Comp.	Lag sel.	RMSE base	RMSE +Cq	Skill	Win frac.	p	Lag used
Chihuahua, full period	% with pneumonia	leadtime	B3	fixed	15.523	16.371	−0.055	0.25	0.092	1
Chihuahua, full period	% with pneumonia	leadtime	B3	internal	17.921	20.876	−0.165	0.364	0.365	1
Chihuahua, full period	% with pneumonia	nowcast	B1	fixed	12.247	13.092	−0.069	0.364	0.849	1
Chihuahua, full period	% with pneumonia	nowcast	B1	internal	12.247	13.592	−0.11	0.409	0.187	1
Chihuahua, full period	% with pneumonia	nowcast	B2	fixed	13.975	14.374	−0.029	0.471	0.818	1
Chihuahua, full period	% with pneumonia	nowcast	B2	internal	14.385	15.486	−0.077	0.375	0.252	1
Chihuahua, full period	% with pneumonia	nowcast	B3	fixed	15.523	16.371	−0.055	0.25	0.092	1
Chihuahua, full period	% with pneumonia	nowcast	B3	internal	16.213	19.696	−0.215	0.364	0.32	1
Chihuahua, full period	Hospitalization rate	leadtime	B1	fixed	0.59	0.679	−0.15	0.267	0.151	9
Chihuahua, full period	Hospitalization rate	leadtime	B1	internal	0.33	0.384	−0.163	0.227	0.007	2
Chihuahua, full period	Hospitalization rate	leadtime	B2	fixed	0.63	0.789	−0.252	0.2	0.027	9
Chihuahua, full period	Hospitalization rate	leadtime	B2	internal	0.278	0.338	−0.215	0.25	0.034	2
Chihuahua, full period	Hospitalization rate	leadtime	B3	internal	0.346	0.475	−0.373	0.091	0.005	2
Chihuahua, full period	Hospitalization rate	nowcast	B1	fixed	0.186	0.351	−0.884	0.2	0.005	9
Chihuahua, full period	Hospitalization rate	nowcast	B1	internal	0.299	0.328	−0.095	0.318	0.019	2
Chihuahua, full period	Hospitalization rate	nowcast	B2	fixed	0.161	0.301	−0.876	0.2	0.049	9
Chihuahua, full period	Hospitalization rate	nowcast	B2	internal	0.189	0.231	−0.221	0.25	0.021	2
Chihuahua, full period	Hospitalization rate	nowcast	B3	internal	0.207	0.245	−0.184	0.091	0.014	2
Chihuahua, full period	Hospitalized cases	leadtime	B1	fixed	23.403	26.916	−0.15	0.267	0.151	9
Chihuahua, full period	Hospitalized cases	leadtime	B1	internal	13.085	15.215	−0.163	0.227	0.007	2
Chihuahua, full period	Hospitalized cases	leadtime	B2	fixed	25.086	30.928	−0.233	0.2	0.049	9
Chihuahua, full period	Hospitalized cases	leadtime	B2	internal	10.822	13.458	−0.244	0.312	0.065	2
Chihuahua, full period	Hospitalized cases	leadtime	B3	internal	14.171	19.116	−0.349	0.273	0.024	2
Chihuahua, full period	Hospitalized cases	nowcast	B1	fixed	7.378	13.902	−0.884	0.2	0.005	9
Chihuahua, full period	Hospitalized cases	nowcast	B1	internal	11.867	12.994	−0.095	0.318	0.019	2
Chihuahua, full period	Hospitalized cases	nowcast	B2	fixed	7.05	13.639	−0.935	0.3	0.037	9
Chihuahua, full period	Hospitalized cases	nowcast	B2	internal	7.979	9.752	−0.222	0.25	0.065	2
Chihuahua, full period	Hospitalized cases	nowcast	B3	internal	8.844	10.57	−0.195	0.091	0.007	2
Chihuahua, full period	Pneumonia cases	leadtime	B1	fixed	19.17	21.527	−0.123	0.267	0.208	9
Chihuahua, full period	Pneumonia cases	leadtime	B1	internal	9.872	11.852	−0.201	0.429	0.089	2
Chihuahua, full period	Pneumonia cases	leadtime	B2	fixed	19.488	24.176	−0.241	0.2	0.064	9
Chihuahua, full period	Pneumonia cases	leadtime	B2	internal	9.422	11.596	−0.231	0.375	0.159	2
Chihuahua, full period	Pneumonia cases	leadtime	B3	internal	11.38	17.075	−0.5	0.091	0.003	2

continues on the next page

Table S6b, continued

Unit	Outcome	Frame	Comp.	Lag sel.	RMSE base	RMSE +Cq	Skill	Win frac.	p	Lag used
Chihuahua, full period	Pneumonia cases	nowcast	B1	fixed	6.498	10.551	−0.624	0.267	0.018	9
Chihuahua, full period	Pneumonia cases	nowcast	B1	internal	8.124	8.997	−0.107	0.429	0.128	2
Chihuahua, full period	Pneumonia cases	nowcast	B2	fixed	6.166	11.835	−0.919	0.2	0.064	9
Chihuahua, full period	Pneumonia cases	nowcast	B2	internal	6.952	8.726	−0.255	0.25	0.025	2
Chihuahua, full period	Pneumonia cases	nowcast	B3	internal	7.214	9.62	−0.333	0.273	0.102	2
Chihuahua, full period	Pneumonia rate	leadtime	B1	fixed	0.483	0.543	−0.123	0.267	0.208	9
Chihuahua, full period	Pneumonia rate	leadtime	B1	internal	0.249	0.299	−0.201	0.429	0.089	2
Chihuahua, full period	Pneumonia rate	leadtime	B2	fixed	0.482	0.63	−0.309	0.1	0.027	9
Chihuahua, full period	Pneumonia rate	leadtime	B2	internal	0.26	0.314	−0.208	0.375	0.144	2
Chihuahua, full period	Pneumonia rate	leadtime	B3	internal	0.304	0.44	−0.447	0.091	0.003	2
Chihuahua, full period	Pneumonia rate	nowcast	B1	fixed	0.164	0.266	−0.624	0.267	0.018	9
Chihuahua, full period	Pneumonia rate	nowcast	B1	internal	0.205	0.227	−0.107	0.429	0.128	2
Chihuahua, full period	Pneumonia rate	nowcast	B2	fixed	0.134	0.233	−0.739	0.3	0.322	9
Chihuahua, full period	Pneumonia rate	nowcast	B2	internal	0.158	0.199	−0.26	0.25	0.025	2
Chihuahua, full period	Pneumonia rate	nowcast	B3	internal	0.152	0.208	−0.366	0.273	0.042	2
Chihuahua, full period	Rt (DGE cases)	leadtime	B1	fixed	0.263	0.253	0.038	0.312	0.144	8
Chihuahua, full period	Rt (DGE cases)	leadtime	B1	internal	0.334	0.353	−0.057	0.591	0.899	2
Chihuahua, full period	Rt (DGE cases)	leadtime	B2	fixed	0.27	0.276	−0.021	0.182	0.102	8
Chihuahua, full period	Rt (DGE cases)	leadtime	B2	internal	0.258	0.36	−0.396	0.235	0.013	2
Chihuahua, full period	Rt (DGE cases)	nowcast	B1	fixed	0.312	0.24	0.232	0.875	0.013	8
Chihuahua, full period	Rt (DGE cases)	nowcast	B1	internal	0.32	0.409	−0.28	0.409	0.276	2
Chihuahua, full period	Rt (DGE cases)	nowcast	B2	fixed	0.306	0.268	0.124	0.818	0.054	8
Chihuahua, full period	Rt (DGE cases)	nowcast	B2	internal	0.275	0.351	−0.276	0.412	0.329	2
Chihuahua, full period	Rt (RT-PCR cases)	leadtime	B1	fixed	0.263	0.234	0.109	0.75	0.159	8
Chihuahua, full period	Rt (RT-PCR cases)	leadtime	B1	internal	0.555	0.364	0.344	0.818	0.001	2
Chihuahua, full period	Rt (RT-PCR cases)	leadtime	B2	fixed	0.26	0.254	0.023	0.455	0.7	8
Chihuahua, full period	Rt (RT-PCR cases)	leadtime	B2	internal	0.351	0.326	0.071	0.647	0.459	2
Chihuahua, full period	Rt (RT-PCR cases)	nowcast	B1	fixed	0.494	0.224	0.546	0.875	<0.001	8
Chihuahua, full period	Rt (RT-PCR cases)	nowcast	B1	internal	0.565	0.342	0.395	0.864	<0.001	2
Chihuahua, full period	Rt (RT-PCR cases)	nowcast	B2	fixed	0.385	0.243	0.368	0.727	0.067	8
Chihuahua, full period	Rt (RT-PCR cases)	nowcast	B2	internal	0.366	0.307	0.16	0.647	0.243	2
Jalisco wave	DGE-confirmed case rate	leadtime	B1	fixed	2.775	2.08	0.25	0.714	0.017	9
Jalisco wave	DGE-confirmed case rate	leadtime	B1	internal	2.47	2.141	0.133	0.625	0.093	7

continues on the next page

Table S6b, continued

Unit	Outcome	Frame	Comp.	Lag sel.	RMSE base	RMSE +Cq	Skill	Win frac.	p	Lag used
Jalisco wave	DGE-confirmed case rate	leadtime	B2	fixed	2.691	1.994	0.259	1	0.008	9
Jalisco wave	DGE-confirmed case rate	leadtime	B2	internal	2.174	1.764	0.189	0.8	0.131	7
Jalisco wave	DGE-confirmed case rate	nowcast	B1	fixed	0.346	0.598	−0.725	0.429	0.119	9
Jalisco wave	DGE-confirmed case rate	nowcast	B1	internal	0.367	0.701	−0.909	0.312	0.025	7
Jalisco wave	DGE-confirmed case rate	nowcast	B2	fixed	0.317	0.643	−1.027	0.444	0.359	9
Jalisco wave	DGE-confirmed case rate	nowcast	B2	internal	0.301	0.587	−0.95	0.2	0.105	7
Jalisco wave	DGE-confirmed case rate	nowcast	B3	internal	0.126	0.815	−5.47	0.167	0.094	7
Jalisco wave	DGE-confirmed cases	leadtime	B1	fixed	248.997	186.599	0.251	0.714	0.017	9
Jalisco wave	DGE-confirmed cases	leadtime	B1	internal	221.583	192.067	0.133	0.625	0.093	7
Jalisco wave	DGE-confirmed cases	leadtime	B2	fixed	254.901	187.57	0.264	1	0.008	9
Jalisco wave	DGE-confirmed cases	leadtime	B2	internal	208.168	166.582	0.2	0.9	0.064	7
Jalisco wave	DGE-confirmed cases	nowcast	B1	fixed	31.068	53.793	−0.731	0.429	0.104	9
Jalisco wave	DGE-confirmed cases	nowcast	B1	internal	32.944	63.097	−0.915	0.312	0.021	7
Jalisco wave	DGE-confirmed cases	nowcast	B2	fixed	24.773	64.191	−1.591	0.222	0.02	9
Jalisco wave	DGE-confirmed cases	nowcast	B2	internal	23.554	53.518	−1.272	0.1	0.02	7
Jalisco wave	DGE-confirmed cases	nowcast	B3	internal	11.289	76.25	−5.754	0.333	0.156	7
Jalisco wave	RT-PCR case rate	leadtime	B1	fixed	2.69	2.013	0.252	0.714	0.017	9
Jalisco wave	RT-PCR case rate	leadtime	B1	internal	2.396	2.076	0.133	0.625	0.105	7
Jalisco wave	RT-PCR case rate	leadtime	B2	fixed	2.662	1.962	0.263	1	0.008	9
Jalisco wave	RT-PCR case rate	leadtime	B2	internal	2.15	1.738	0.192	0.8	0.16	7
Jalisco wave	RT-PCR case rate	nowcast	B1	fixed	0.345	0.592	−0.716	0.429	0.104	9
Jalisco wave	RT-PCR case rate	nowcast	B1	internal	0.355	0.713	−1.012	0.312	0.021	7
Jalisco wave	RT-PCR case rate	nowcast	B2	fixed	0.316	0.631	−0.994	0.444	0.359	9
Jalisco wave	RT-PCR case rate	nowcast	B2	internal	0.3	0.615	−1.049	0.3	0.131	7
Jalisco wave	RT-PCR case rate	nowcast	B3	internal	0.123	0.889	−6.247	0.167	0.094	7
Jalisco wave	RT-PCR-positive cases	leadtime	B1	fixed	241.46	180.653	0.252	0.714	0.017	9
Jalisco wave	RT-PCR-positive cases	leadtime	B1	internal	214.936	186.242	0.134	0.625	0.105	7
Jalisco wave	RT-PCR-positive cases	leadtime	B2	fixed	247.484	183.271	0.259	1	0.008	9
Jalisco wave	RT-PCR-positive cases	leadtime	B2	internal	202.167	162.763	0.195	0.9	0.064	7
Jalisco wave	RT-PCR-positive cases	nowcast	B1	fixed	30.949	53.3	−0.722	0.357	0.091	9
Jalisco wave	RT-PCR-positive cases	nowcast	B1	internal	31.811	64.2	−1.018	0.25	0.018	7
Jalisco wave	RT-PCR-positive cases	nowcast	B2	fixed	24.206	62.686	−1.59	0.111	0.012	9
Jalisco wave	RT-PCR-positive cases	nowcast	B2	internal	23.007	55.957	−1.432	0.1	0.027	7

continues on the next page

Table S6b, continued

Unit	Outcome	Frame	Comp.	Lag sel.	RMSE base	RMSE +Cq	Skill	Win frac.	p	Lag used
Jalisco wave	RT-PCR-positive cases	nowcast	B3	internal	11.078	82.978	−6.491	0.167	0.094	7
Jalisco wave	% hospitalized	leadtime	B1	fixed	11.672	10.756	0.078	0.45	0.985	3
Jalisco wave	% hospitalized	leadtime	B1	internal	11.024	10.991	0.003	0.476	0.892	2
Jalisco wave	% hospitalized	leadtime	B2	fixed	13.992	12.86	0.081	0.429	0.761	3
Jalisco wave	% hospitalized	leadtime	B2	internal	13.298	13.348	−0.004	0.533	0.89	2
Jalisco wave	% hospitalized	leadtime	B3	fixed	16.397	14.68	0.105	0.444	0.91	3
Jalisco wave	% hospitalized	leadtime	B3	internal	16.02	15.561	0.029	0.6	0.432	2
Jalisco wave	% hospitalized	nowcast	B1	fixed	11.821	10.932	0.075	0.45	0.784	3
Jalisco wave	% hospitalized	nowcast	B1	internal	11.554	11.652	−0.008	0.619	0.633	2
Jalisco wave	% hospitalized	nowcast	B2	fixed	11.113	9.423	0.152	0.667	0.303	3
Jalisco wave	% hospitalized	nowcast	B2	internal	10.767	10.833	−0.006	0.625	0.433	2
Jalisco wave	% hospitalized	nowcast	B3	fixed	13.284	10.438	0.214	0.6	0.232	3
Jalisco wave	% hospitalized	nowcast	B3	internal	12.734	12.437	0.023	0.636	0.638	2
Jalisco wave	% with pneumonia	leadtime	B1	fixed	6.682	6.756	−0.011	0.409	0.321	1
Jalisco wave	% with pneumonia	leadtime	B1	internal	6.528	6.643	−0.018	0.476	0.203	2
Jalisco wave	% with pneumonia	leadtime	B2	fixed	7.401	7.226	0.024	0.562	0.9	1
Jalisco wave	% with pneumonia	leadtime	B2	internal	7.425	7.422	0	0.533	0.359	2
Jalisco wave	% with pneumonia	leadtime	B3	fixed	9.051	8.808	0.027	0.364	0.52	1
Jalisco wave	% with pneumonia	leadtime	B3	internal	9.371	9.197	0.019	0.556	0.652	2
Jalisco wave	% with pneumonia	nowcast	B1	fixed	6.682	6.756	−0.011	0.409	0.321	1
Jalisco wave	% with pneumonia	nowcast	B1	internal	6.836	6.571	0.039	0.381	0.272	2
Jalisco wave	% with pneumonia	nowcast	B2	fixed	7.401	7.226	0.024	0.562	0.9	1
Jalisco wave	% with pneumonia	nowcast	B2	internal	7.401	6.829	0.077	0.562	0.94	2
Jalisco wave	% with pneumonia	nowcast	B3	fixed	9.051	8.808	0.027	0.364	0.52	1
Jalisco wave	% with pneumonia	nowcast	B3	internal	9.051	8.343	0.078	0.545	0.7	2
Jalisco wave	Hospitalization rate	leadtime	B1	fixed	0.245	0.197	0.197	0.714	0.007	9
Jalisco wave	Hospitalization rate	leadtime	B1	internal	0.206	0.208	−0.008	0.625	0.159	7
Jalisco wave	Hospitalization rate	leadtime	B2	fixed	0.283	0.204	0.28	1	0.008	9
Jalisco wave	Hospitalization rate	leadtime	B2	internal	0.228	0.227	0.002	0.8	0.322	7
Jalisco wave	Hospitalization rate	leadtime	B3	internal	0.132	0.176	−0.331	0.714	0.938	7
Jalisco wave	Hospitalization rate	nowcast	B1	fixed	0.059	0.063	−0.069	0.571	0.67	9
Jalisco wave	Hospitalization rate	nowcast	B1	internal	0.059	0.085	−0.428	0.25	0.025	7
Jalisco wave	Hospitalization rate	nowcast	B2	fixed	0.045	0.04	0.105	0.667	0.82	9

continues on the next page

Table S6b, continued

Unit	Outcome	Frame	Comp.	Lag sel.	RMSE base	RMSE +Cq	Skill	Win frac.	p	Lag used
Jalisco wave	Hospitalization rate	nowcast	B2	internal	0.057	0.065	−0.133	0.364	0.465	7
Jalisco wave	Hospitalization rate	nowcast	B3	internal	0.037	0.062	−0.65	0.429	0.469	7
Jalisco wave	Hospitalized cases	leadtime	B1	fixed	22.013	17.679	0.197	0.714	0.007	9
Jalisco wave	Hospitalized cases	leadtime	B1	internal	18.486	18.626	−0.008	0.625	0.159	7
Jalisco wave	Hospitalized cases	leadtime	B2	fixed	25.028	18.816	0.248	1	0.008	9
Jalisco wave	Hospitalized cases	leadtime	B2	internal	19.9	20.769	−0.044	0.8	0.322	7
Jalisco wave	Hospitalized cases	leadtime	B3	internal	12.427	16.035	−0.29	0.143	0.156	7
Jalisco wave	Hospitalized cases	nowcast	B1	fixed	5.309	5.715	−0.077	0.571	0.67	9
Jalisco wave	Hospitalized cases	nowcast	B1	internal	5.319	7.569	−0.423	0.25	0.034	7
Jalisco wave	Hospitalized cases	nowcast	B2	fixed	4.079	3.814	0.065	0.556	0.652	9
Jalisco wave	Hospitalized cases	nowcast	B2	internal	4.482	4.885	−0.09	0.455	0.831	7
Jalisco wave	Hospitalized cases	nowcast	B3	internal	2.574	5.785	−1.248	0.286	0.078	7
Jalisco wave	Pneumonia cases	leadtime	B1	fixed	9.766	8.206	0.16	0.714	0.009	9
Jalisco wave	Pneumonia cases	leadtime	B1	internal	7.413	7.678	−0.036	0.562	0.464	5
Jalisco wave	Pneumonia cases	leadtime	B2	fixed	10.577	8.653	0.182	0.875	0.039	9
Jalisco wave	Pneumonia cases	leadtime	B2	internal	8.099	8.204	−0.013	0.727	0.465	5
Jalisco wave	Pneumonia cases	leadtime	B3	internal	5.828	7.89	−0.354	0.429	0.578	5
Jalisco wave	Pneumonia cases	nowcast	B1	fixed	2.679	3.349	−0.25	0.143	0.001	9
Jalisco wave	Pneumonia cases	nowcast	B1	internal	2.62	3.322	−0.268	0.562	0.562	5
Jalisco wave	Pneumonia cases	nowcast	B2	fixed	2.207	2.658	−0.205	0.444	1	9
Jalisco wave	Pneumonia cases	nowcast	B2	internal	2.067	2.341	−0.132	0.545	0.898	5
Jalisco wave	Pneumonia cases	nowcast	B3	internal	1.875	2.131	−0.137	0.375	0.742	5
Jalisco wave	Pneumonia rate	leadtime	B1	fixed	0.109	0.091	0.16	0.714	0.009	9
Jalisco wave	Pneumonia rate	leadtime	B1	internal	0.081	0.084	−0.033	0.556	0.393	5
Jalisco wave	Pneumonia rate	leadtime	B2	fixed	0.12	0.09	0.253	1	0.008	9
Jalisco wave	Pneumonia rate	leadtime	B2	internal	0.092	0.092	−0.003	0.727	0.638	5
Jalisco wave	Pneumonia rate	leadtime	B3	internal	0.064	0.088	−0.382	0.429	0.297	5
Jalisco wave	Pneumonia rate	nowcast	B1	fixed	0.03	0.037	−0.243	0.143	0.001	9
Jalisco wave	Pneumonia rate	nowcast	B1	internal	0.035	0.042	−0.18	0.556	0.64	5
Jalisco wave	Pneumonia rate	nowcast	B2	fixed	0.031	0.038	−0.231	0.111	0.027	9
Jalisco wave	Pneumonia rate	nowcast	B2	internal	0.029	0.036	−0.257	0.727	0.765	5
Jalisco wave	Pneumonia rate	nowcast	B3	internal	0.031	0.037	−0.189	0.625	0.945	5
Jalisco wave	Rt (DGE cases)	leadtime	B1	fixed	0.418	0.355	0.152	0.75	0.105	7

continues on the next page

Table S6b, continued

Unit	Outcome	Frame	Comp.	Lag sel.	RMSE base	RMSE +Cq	Skill	Win frac.	p	Lag used
Jalisco wave	Rt (DGE cases)	leadtime	B1	internal	0.402	0.388	0.035	0.632	0.293	4
Jalisco wave	Rt (DGE cases)	leadtime	B2	fixed	0.244	0.293	−0.202	0.5	1	7
Jalisco wave	Rt (DGE cases)	leadtime	B2	internal	0.187	0.38	−1.034	0.385	0.305	4
Jalisco wave	Rt (DGE cases)	nowcast	B1	fixed	0.212	0.224	−0.056	0.562	0.782	7
Jalisco wave	Rt (DGE cases)	nowcast	B1	internal	0.242	0.267	−0.106	0.526	0.798	4
Jalisco wave	Rt (DGE cases)	nowcast	B2	fixed	0.194	0.253	−0.302	0.455	0.52	7
Jalisco wave	Rt (DGE cases)	nowcast	B2	internal	0.19	0.293	−0.541	0.385	0.34	4
Jalisco wave	Rt (RT-PCR cases)	leadtime	B1	fixed	0.422	0.36	0.147	0.75	0.144	7
Jalisco wave	Rt (RT-PCR cases)	leadtime	B1	internal	0.403	0.41	−0.019	0.737	0.312	4
Jalisco wave	Rt (RT-PCR cases)	leadtime	B2	fixed	0.28	0.327	−0.17	0.6	0.846	7
Jalisco wave	Rt (RT-PCR cases)	leadtime	B2	internal	0.221	0.417	−0.887	0.462	0.497	4
Jalisco wave	Rt (RT-PCR cases)	nowcast	B1	fixed	0.221	0.259	−0.173	0.562	0.98	7
Jalisco wave	Rt (RT-PCR cases)	nowcast	B1	internal	0.252	0.301	−0.199	0.579	0.891	4
Jalisco wave	Rt (RT-PCR cases)	nowcast	B2	fixed	0.21	0.298	−0.421	0.455	0.52	7
Jalisco wave	Rt (RT-PCR cases)	nowcast	B2	internal	0.212	0.34	−0.606	0.462	0.588	4

p is from a Wilcoxon signed-rank test on the per-fold difference in absolute error. Win fraction is the proportion of folds in which the model including Cq had the smaller absolute error. Continuous estimates are displayed to three decimal places; the source data files retain full numerical precision. Rows appear in the same order as in Table S6a.

Table S7. STROBE checklist for reports of observational studies, with the location of each item in this article.

Item	Recommendation	Reported in
**1a**	Indicate the study's design with a commonly used term in the title or the abstract	Title ("A Time Series Analysis"); Abstract, Methods
**1b**	Provide in the abstract an informative and balanced summary of what was done and what was found	Abstract, structured under Objectives, Methods, Results and Conclusions
**2**	Explain the scientific background and rationale for the investigation being reported	Introduction
**3**	State specific objectives, including any prespecified hypotheses	Introduction, final paragraph; Abstract, Objectives
**4**	Present key elements of study design early in the paper	Introduction, final paragraph; Materials and Methods, Statistical analysis
**5**	Describe the setting, locations, and relevant dates, including periods of recruitment, exposure, follow-up, and data collection	Materials and Methods, Setting
**6a**	Give the eligibility criteria, and the sources and methods of selection of participants	Materials and Methods, Population and eligibility criteria; Figure 1
**6b**	For matched studies, give matching criteria and number of exposed and unexposed	Not applicable: the study is not matched
**7**	Clearly define all outcomes, exposures, predictors, potential confounders, and effect modifiers. Give diagnostic criteria, if applicable	Materials and Methods, Data metrics for time-series analysis, and Laboratory diagnosis and comparability of Cq values; Tables S0a and S0b
**8**	For each variable of interest, give sources of data and details of methods of assessment. Describe comparability of assessment methods if there is more than one group	Materials and Methods, Data sources and access, and Laboratory diagnosis and comparability of Cq values; Table S0a
**9**	Describe any efforts to address potential sources of bias	Materials and Methods, Statistical analysis (adjustment for the secular trend, specimen composition and the outcome's own past); Discussion, limitations
**10**	Explain how the study size was arrived at	Materials and Methods, Population and eligibility criteria; Figure 1. No target size was set: every record meeting the criteria in the two outbreaks was analyzed
**11**	Explain how quantitative variables were handled in the analyses. If applicable, describe which groupings were chosen and why	Materials and Methods, Data metrics for time-series analysis, and Statistical analysis
**12a**	Describe all statistical methods, including those used to control for confounding	Materials and Methods, Statistical analysis, with the estimating equations
**12b**	Describe any methods used to examine subgroups and interactions	Materials and Methods, Statistical analysis; Results, Individual-level Cq determinations; Table 3
**12c**	Explain how missing data were addressed	Materials and Methods, Population and eligibility criteria; Results, Descriptive characteristics; Figure 1; Table S0a
**12d**	If applicable, describe analytical methods taking account of sampling strategy	Not applicable: the analysis is a complete enumeration of notified cases, not a sample
**12e**	Describe any sensitivity analyses	Results, Cross-correlation analysis, Lagged regression analysis, Toda–Yamamoto Granger causality and calibrated-lag autoregressive models, and Individual-level Cq determinations; Tables S1c, S4b, S5 and S9 to S12b; Figure S1
**13a**	Report numbers of individuals at each stage of the study	Figure 1
**13b**	Give reasons for non-participation at each stage	Figure 1, exclusion boxes
**13c**	Consider use of a flow diagram	Figure 1
**14a**	Give characteristics of study participants and information on exposures and potential confounders	Results, Descriptive characteristics, and Individual-level Cq determinations; Figures 2 and 5
**14b**	Indicate number of participants with missing data for each variable of interest	Results, Descriptive characteristics; Figure 1

continues on the next page

Table S7, continued

Item	Recommendation	Reported in
**15**	Report numbers of outcome events or summary measures	Results, Descriptive characteristics and Wave structure; Figures 1 and 2
**16a**	Give unadjusted estimates and, if applicable, confounder-adjusted estimates and their precision	Results, Lagged regression analysis; Figure 4; Tables S2a and S2b, which report the unadjusted model and three nested adjusted models
**16b**	Report category boundaries when continuous variables were categorized	Results, Individual-level Cq determinations; Table S11. Documented vaccine dose was examined both as a linear term over zero, one and two doses and as a three-level factor. No other continuous variable was categorized
**16c**	If relevant, consider translating estimates of relative risk into absolute risk	Not applicable: no relative risks are estimated
**17**	Report other analyses done, for example analyses of subgroups and interactions, and sensitivity analyses	Results, Cross-correlation analysis, Lagged regression analysis, Toda–Yamamoto Granger causality and calibrated-lag autoregressive models, Individual-level Cq determinations, and Out-of-sample predictive gain; Tables S1c, S4a to S6b and S9 to S12b; Figure S1
**18**	Summarise key results with reference to study objectives	Discussion, opening paragraphs; Conclusions
**19**	Discuss limitations of the study, taking into account sources of potential bias or imprecision. Discuss both direction and magnitude of any potential bias	Discussion, limitations paragraphs
**20**	Give a cautious overall interpretation of results considering objectives, limitations, multiplicity of analyses, results from similar studies, and other relevant evidence	Discussion; Conclusions
**21**	Discuss the generalisability (external validity) of the study results	Discussion, limitations paragraphs and future directions; Conclusions
**22**	Give the source of funding and the role of the funders for the present study and, if applicable, for the original study on which the present article is based	Funding statement

Locations are given as section names rather than page numbers, which change with typesetting. As stated in Materials and Methods, both instruments are followed as written for the primary weekly time-series analysis and the secondary individual-level analysis. The counts of individuals behind each week are given in Figure 1. A reason is provided for each item marked not applicable.

Table S8. RECORD checklist, the extension of STROBE for studies conducted using routinely collected health data, with the location of each item in this article.

Item	Recommendation	Reported in
**1.1**	The type of data used should be specified in the title or abstract. When possible, the name of the databases used should be included	Abstract, Methods, which names the Mexican national surveillance system; Materials and Methods, Data sources and access
**1.2**	If applicable, the geographic region and timeframe within which the study took place should be reported in the title or abstract	Title ("the 2025–2026 Outbreak in Mexico"); Abstract, Methods, which names Chihuahua and Jalisco
**1.3**	If linkage between databases was conducted for the study, this should be clearly stated in the title or abstract	Not applicable. Abstract, Methods, and Materials and Methods, Data sources and access state that all person-level information came from the same EFE/SINAVE case-level surveillance record. No external person-level database was linked for this study
**6.1**	The methods of study population selection, such as codes or algorithms used to identify subjects, should be listed in detail. If this is not possible, an explanation should be provided	Materials and Methods, Population and eligibility criteria, and Laboratory diagnosis and comparability of Cq values; Figure 1; the deposited analysis code

continues on the next page

Table S8, continued

Item	Recommendation	Reported in
**6.2**	Any validation studies of the codes or algorithms used to select the population should be referenced. If validation was conducted for this study and not published elsewhere, detailed methods and results should be provided	Materials and Methods, Laboratory diagnosis and comparability of Cq values, which cites the national diagnostic algorithm and the validated assay
**6.3**	If the study involved linkage of databases, consider use of a flow diagram or other graphical display to demonstrate the data linkage process, including the number of individuals with linked data at each stage	Not applicable. Figure 1 shows two analytic subsets derived from the same case-level surveillance records, not a database-linkage process
**7.1**	A complete list of codes and algorithms used to classify exposures, outcomes, confounders, and effect modifiers should be provided. If these cannot be reported, an explanation should be provided	Materials and Methods, Population and eligibility criteria, and Data metrics for time-series analysis; Tables S0a and S0b; the deposited analysis code, which carries every operational definition
**12.1**	Authors should describe the extent to which the investigators had access to the database population used to create the study population	Materials and Methods, Data sources and access; Ethics statement
**12.2**	Authors should provide information on the data cleaning methods used in the study	Materials and Methods, Population and eligibility criteria, and Laboratory diagnosis and comparability of Cq values; the deposited analysis code
**12.3**	State whether the study included person-level, institutional-level, or other data linkage across two or more databases. The methods of linkage and methods of linkage quality evaluation should be provided	Materials and Methods, Data sources and access. No linkage across databases was conducted. Laboratory, epidemiological, clinical, vaccination and classification information was obtained from the same EFE/SINAVE case-level surveillance record. Linkage-quality evaluation therefore does not apply
**13.1**	Describe in detail the selection of the persons included in the study, including filtering based on data quality, data availability and linkage. The selection of included persons can be described in the text and/or by means of the study flow diagram	Figure 1; Materials and Methods, Population and eligibility criteria
**19.1**	Discuss the implications of using data that were not created or collected to answer the specific research question(s). Include discussion of misclassification bias, unmeasured confounding, missing data, and changing eligibility over time, as they pertain to the study being reported	Discussion, limitations paragraphs, which cover under-ascertainment, drift in the completeness of the complications field, unmeasured specimen quality, and the exposure as a proxy
**22.1**	Authors should provide information on how to access any supplemental information such as the study protocol, raw data, or programming code	Data Availability Statement, which gives the digital object identifier of the deposit holding the aggregated weekly panels and the analysis code

Locations are given as section names rather than page numbers, which change with typesetting. As stated in Materials and Methods, both instruments are followed as written for the primary weekly time-series analysis and the secondary individual-level analysis. The counts of individuals behind each week are given in Figure 1. A reason is provided for each item marked not applicable.

Table S9. Jalisco endpoint sensitivity after excluding one, two or four endpoint weeks.

Cutoff	Exclusion	Wks	Code	Lag	r	model	n	Pred.	Est.	β	Boot low	Boot high	HAC p	Class. p	R^2
2026-07-20	1 week (primary)	38	rt_lab	7	−0.46	M3	31	4	Yes	−0.107	−0.258	−0.009	0.034	0.081	0.582
2026-07-20	1 week (primary)	38	rt_lab	7	−0.46	M4	31	6	Yes	−0.135	−0.283	−0.021	0.011	0.015	0.716
2026-07-20	1 week (primary)	38	rt_lab	7	−0.46	AR cal.	30	2	Yes	−0.126	−0.279	−0.032	0.06	0.038	0.563
2026-07-20	1 week (primary)	38	rt_dge	7	−0.464	M3	31	4	Yes	−0.091	−0.231	−0.011	0.012	0.077	0.635
2026-07-20	1 week (primary)	38	rt_dge	7	−0.464	M4	31	6	Yes	−0.117	−0.235	−0.025	0.003	0.009	0.772
2026-07-20	1 week (primary)	38	rt_dge	7	−0.464	AR cal.	30	2	Yes	−0.1	−0.225	−0.028	0.036	0.055	0.609
2026-07-13	2 weeks	37	rt_lab	7	−0.482	M3	30	4	Yes	−0.098	−0.268	−0.011	0.057	0.138	0.584
2026-07-13	2 weeks	37	rt_lab	7	−0.482	M4	30	6	Yes	−0.134	−0.287	−0.014	0.025	0.026	0.716
2026-07-13	2 weeks	37	rt_lab	7	−0.482	AR cal.	29	2	Yes	−0.126	−0.298	−0.042	0.095	0.052	0.563
2026-07-13	2 weeks	37	rt_dge	7	−0.485	M3	30	4	Yes	−0.082	−0.23	−0.005	0.025	0.141	0.639
2026-07-13	2 weeks	37	rt_dge	7	−0.485	M4	30	6	Yes	−0.114	−0.255	−0.019	0.013	0.02	0.772
2026-07-13	2 weeks	37	rt_dge	7	−0.485	AR cal.	29	2	Yes	−0.096	−0.233	−0.037	0.085	0.087	0.61
2026-06-29	4 weeks	35	rt_lab	7	−0.548	M3	28	4	Yes	−0.092	−0.304	0.002	0.144	0.211	0.607
2026-06-29	4 weeks	35	rt_lab	7	−0.548	M4	28	6	No						
2026-06-29	4 weeks	35	rt_lab	7	−0.548	AR cal.	27	2	Yes	−0.159	−0.352	−0.051	0.04	0.015	0.625
2026-06-29	4 weeks	35	rt_dge	7	−0.556	M3	28	4	Yes	−0.076	−0.248	0	0.115	0.216	0.667
2026-06-29	4 weeks	35	rt_dge	7	−0.556	M4	28	6	No						
2026-06-29	4 weeks	35	rt_dge	7	−0.556	AR cal.	27	2	Yes	−0.127	−0.271	−0.049	0.019	0.022	0.679

The primary analysis ends in the complete week beginning 20 July 2026, excluding the incomplete week beginning 27 July. The additional cutoffs exclude two or four endpoint weeks in total. At the primary cutoff, the M3 and M4 rows are the canonical primary results also reported in Table S2a and Figure 4. These rows are imported rather than re-estimated. Models at the additional cutoffs were refitted where they met five observations per predictor, excluding the intercept. M3 denotes adjustment for age, sex and a linear time trend; M4 is the fully adjusted model; AR cal. is the calibrated-lag autoregressive model. Moving-block bootstrap intervals are primary, with HAC and classical p-values reported as sensitivities.

Table S10. Jalisco composition-adjustment sensitivity models for the two reproduction-number series.

Outcome	Lag	model	n	Pred.	Est.	β	Boot low	Boot high	HAC p	Class. p	R^2
rt_lab	7	M3 reference	31	4	Yes	−0.107	−0.258	−0.009	0.034	0.081	0.582
rt_lab	7	M4 reference	31	6	Yes	−0.135	−0.283	−0.021	0.011	0.015	0.716
rt_lab	7	M3 + weekly unvaccinated proportion	31	5	Yes	−0.117	−0.267	−0.009	0.024	0.036	0.681
rt_lab	7	M3 + median rash-to-collection interval	31	5	Yes	−0.101	−0.293	−0.002	0.106	0.121	0.583
rt_lab	7	M3 + non-analyzable Cq proportion	31	5	Yes	−0.126	−0.27	−0.013	0.009	0.023	0.692
rt_lab	7	M3 + vaccination composition + collection interval	31	6	Yes	−0.12	−0.276	0.012	0.039	0.045	0.681
rt_lab	7	M3 + all three added weekly covariates	31	7	No						
rt_dge	7	M3 reference	31	4	Yes	−0.091	−0.231	−0.011	0.012	0.077	0.635
rt_dge	7	M4 reference	31	6	Yes	−0.117	−0.235	−0.025	0.003	0.009	0.772
rt_dge	7	M3 + weekly unvaccinated proportion	31	5	Yes	−0.099	−0.226	0.002	0.011	0.041	0.701
rt_dge	7	M3 + median rash-to-collection interval	31	5	Yes	−0.082	−0.237	−0.01	0.072	0.136	0.64
rt_dge	7	M3 + non-analyzable Cq proportion	31	5	Yes	−0.107	−0.22	−0.015	0.002	0.023	0.725
rt_dge	7	M3 + vaccination composition + collection interval	31	6	Yes	−0.095	−0.238	0.015	0.027	0.068	0.702
rt_dge	7	M3 + all three added weekly covariates	31	7	No						

The M3 reference and M4 reference rows are the canonical primary results also reported in Table S2a and Figure 4. These rows are imported rather than re-estimated. Each additional sensitivity adds the stated weekly composition variable to the reference M3 specification. Percentage unvaccinated is calculated among analyzable Cq determinations with known vaccination status. The median collection interval is the weekly median number of days from rash onset to specimen collection. Percentage non-analyzable is the weekly fraction of candidate measles throat-swab blocks without a valid $0 < Cq < 40$ value. Joint models are displayed only when they met the prespecified estimability rule. Moving-block bootstrap intervals are primary. HAC and classical p-values are sensitivities.

Table S11. Sensitivity analyses of the individual-level association between documented vaccine dose and Cq.

**Model**	**Term**	**n**	**Estimate**	**Robust low**	**Robust high**	**Robust p**	**Adj. R^2**
Linear dose; linear age	dose	7576	0.761	0.573	0.948	<0.001	0.103
Linear dose; spline age	dose	7576	0.758	0.57	0.945	<0.001	0.11
Dose as factor; spline age	dose_factor1	7576	0.673	0.383	0.963	<0.001	0.11
Dose as factor; spline age	dose_factor2	7576	1.602	1.144	2.06	<0.001	0.11
Dose-by-state interaction; spline age	dose	7576	1.096	0.776	1.415	<0.001	0.111
Dose-by-state interaction; spline age	dose:DES_EDOJALISCO	7576	−0.544	−0.935	−0.153	0.006	0.111
Collection interval by dose; spline age	dose	7576	−0.029	−0.114	0.056	0.501	0.018
Dose as factor; spline age	Joint dose-factor test	7576				<0.001	0.11
Dose-by-state interaction; spline age	Dose slope in Jalisco	7576	0.552	0.324	0.78	<0.001	0.111

All models use heteroskedasticity-robust inference and adjust for the terms stated in the model label. Dose was examined as a linear term, as a factor, with age represented by a restricted cubic spline, and with a dose-by-state interaction. The direct comparison of collection interval by dose evaluates whether later specimen collection differed linearly across the zero-, one- and two-dose groups.

Table S12a. Sensitivity analysis treating the two Chihuahua waves as one undecomposed series.

Outcome	Weeks	Cq weeks	Raw lag	Raw r	Raw p	Raw n	ARIMA	PW best lag	PW r	PW p	PW n
casos_lab	43	38	8	−0.15	0.422	31	ARIMA(3,1,0)	5	−0.168	0.334	35
casos_dge	43	38	8	−0.169	0.365	31	ARIMA(3,1,0)	5	−0.197	0.257	35
tasa_lab	43	38	8	−0.15	0.422	31	ARIMA(3,1,0)	5	−0.168	0.334	35
tasa_dge	43	38	8	−0.169	0.365	31	ARIMA(3,1,0)	5	−0.197	0.257	35
rt_lab	43	38	8	−0.37	0.04	31	ARIMA(3,1,0)	1	−0.383	0.016	39
rt_dge	43	38	8	−0.38	0.035	31	ARIMA(3,1,0)	1	−0.38	0.017	39
hosp_n	43	38	1	−0.234	0.163	37	ARIMA(3,1,0)	1	−0.252	0.122	39
hosp_tasa	43	38	1	−0.234	0.163	37	ARIMA(3,1,0)	1	−0.252	0.122	39
hosp_prop	43	38	1	−0.215	0.201	37	ARIMA(3,1,0)	8	−0.397	0.024	32
neum_n	43	38	1	−0.199	0.237	37	ARIMA(3,1,0)	1	−0.161	0.326	39
neum_tasa	43	38	1	−0.199	0.237	37	ARIMA(3,1,0)	1	−0.161	0.326	39
neum_prop	43	38	1	−0.35	0.034	37	ARIMA(3,1,0)	8	−0.293	0.104	32

The raw and prewhitened cross-correlations were recalculated over the complete Chihuahua study window without splitting it into two waves. ARIMA prewhitening follows the same procedure as Table S1c. No correction for multiplicity was applied.

Table S12b. Jalisco Toda–Yamamoto sensitivity analysis with $d_{\max} = 2$.

**Outcome**	**Weeks**	**Est.**	p	$d_{\max}$	**Lags**	**Obs./par.**	**TY** χ^2	**TY** p	**TY** p **HAC**	**Rev.** p
rt_lab	38	Yes	1	2	3	5	1.837	0.175	0.091	0.651
rt_dge	38	Yes	1	2	3	5	1.3	0.254	0.14	0.728

The VAR was fitted in levels with $p + d_{\max}$ lags, and the Wald restriction was applied to the first p Cq lags. Classical and HAC Wald p-values are shown. This sensitivity addresses the fact that not every series met both stationarity criteria after one difference. It did not provide formal evidence of Cq-to-R_t Granger causality.

Table S13. Operational timeliness from specimen collection to the laboratory-result date recorded in the surveillance file.

**Series**	**Analytic n**	**Date-order errors**	**Intervals**	**Median (IQR), days**	**Within 7 days (%)**
Both states	10910	20	10890	3 (2-—5)	94.3
Chihuahua	3874	20	3854	4 (3-—6)	88.1
Jalisco	7036	0	7036	2 (2-—4)	97.8

All 10,910 analytic Cq determinations had a recorded laboratory-result date. The 20 records in which that date preceded specimen collection were reported as date-order inconsistencies and excluded only from interval summaries. Percentages are calculated among records with non-negative intervals. The recorded result date is an operational proxy for result generation. The source does not establish when the information became accessible to, or was used by, public-health decision-makers.
